# Twist degree of freedom activated by pressure-driven interlayer engineering in spiral WS_2_

**DOI:** 10.1038/s41467-026-77227-3

**Published:** 2026-09-16

**Authors:** Penghong Ci, Muhua Sun, Yabin Chen, Yoonsoo Rho, Yuzhou Zhao, Kaiyao Xin, Zifeng Mai, Shanyu Liang, Zhongjie Wang, Jiangbin Wu, Hao Hong, Zhongming Wei, Costas P. Grigoropoulos, Yue-Yang Liu, Ping-Heng Tan, Junqiao Wu

**Affiliations:** 1 State Key Laboratory of Semiconductor Physics and Chip Technologies, Institute of Semiconductors, Chinese Academy of Sciences, Beijing, China; 2 Key Laboratory of Advanced Materials (MOE), School of Materials Science and Engineering, Tsinghua University, Beijing, China; 3 Department of Materials Science and Engineering, University of California, Berkeley, CA USA; 4 Department of Mechanical Engineering, Ulsan National Institute of Science and Technology (UNIST), Ulsan, Republic of Korea; 5 Department of Mechanical Engineering, University of California, Berkeley, CA USA; 6 School of Physical Science and Technology, ShanghaiTech University, Shanghai, China; 7 State Key Lab for Mesoscopic Physics and Frontiers Science Center for Nano-optoelectronics, School of Physics, Peking University, Beijing, China

**Keywords:** Structural properties, Mechanical engineering, Two-dimensional materials, Two-dimensional materials

## Abstract

Precise twist-angle control in van der Waals (vdW) moiré superlattices enables twistronic tuning of interfacial responses and emergent quantum states, but it remains unclear how atomic registry in three-dimensional stacks is linked to interlayer coupling and whether it can produce an internal twist degree of freedom without external torque. Here, we show that, via interlayer engineering, screw-dislocation-driven multilayer spiral WS_2_ exhibits a reversible pressure-induced rotation of second-harmonic-generation polar pattern that saturates near 1 GPa, corresponding to an effective average interlayer twist of 0.2° per layer. This compression-twist coupling arises from moiré defined hierarchy of stacking energies and the associated atomic reconstruction, which together generate a spontaneous rotational torque at a slipped, metastable interface and couple the twist angle to the interlayer spacing. Our findings establish twist-degree-of-freedom manipulation in vdW crystals, opening pathways to reshape correlation-driven behaviors in moiré-of-moiré architectures and to explore chiral elasticity beyond Cauchy continuum mechanics.

## Abstract (web summary)

Precise twist-angle control in van der Waals (vdW) moiré superlattices enables the observation of emerging physical properties. Here, the authors demonstrate a reversible pressure-controlled rotational response in multilayer spiral WS_2_, corresponding to an effective average interlayer twist of 0.2° per layer.

## Introduction

Moiré superlattices formed by stacking van der Waals (vdW) layers with small rotational offsets or lattice mismatch provide a versatile platform to engineer electronic and optical behavior, hosting moiré inter- and intralayer excitons and trions^1,2^, hybridized electronic states^3,4^, or correlated and topological quantum phases^5–8^. These emergent phenomena are governed by spatially varying atomic registry, which periodically modulates interlayer coupling and local stacking energies and can drive intrinsic lattice reconstruction to reshape the moiré potential depth^9^, band flatness^10,11^, topology^12–15^, and real-space carrier localization^16–18^. Extending to three-dimensional multilayer stacks (3D twistronics) introduces hierarchical interactions among incommensurate moiré-of-moiré superstructures, which can accommodate additional flat bands and manifest exotic behaviors^19,20^, including topological phase transitions^21^, novel resonant states^22,23^, the opto-twistronic Hall effect^24^, and screw-dislocation-enabled helical current pathways^25^. In practice, these moiré and moiré-of-moiré patterns are usually defined during tear-and-stack transfer^26^, producing device-grade but non-reconfigurable stacks with fixed twist angles. It is desired to be able to reconfigure moiré patterns via external fields rather than treating twist as a static structural parameter, thereby providing dynamic access to their quantum states.

Thermal annealing can reconfigure the moiré patterns in these mechanically assembled vdW systems by enabling incommensurate states to overcome interfacial energy barriers and relax into energetically favorable commensurate, reconstructed stackings^14,27–30^. However, the rotation is typically restricted to narrow critical-angle regimes and does not yield fine, continuous, or reversible twist control. Ultrafast optical excitation has been shown to transiently induce twist-untwist torsional motion via modulation of the moiré periodic lattice distortion^31^. Yet the average twist angle elastically relaxes back after excitation and does not define a new static configuration. Beyond thermal and optical fields, direct mechanical actuation enables twist manipulation through controlled interfacial torque, implemented via atomic force microscopy (AFM) tip dragging^32^, scanning probe microscopy (SPM) local-contact rotation^33,34^, or microelectromechanical systems (MEMS) on-chip rotary actuation^35^. However, these torque-based techniques demand elaborate, often cryogenic AFM/SPM or custom MEMS architectures and have not yet been generalized to simultaneously modulate multiple moiré interfaces.

**Fig. 1:**
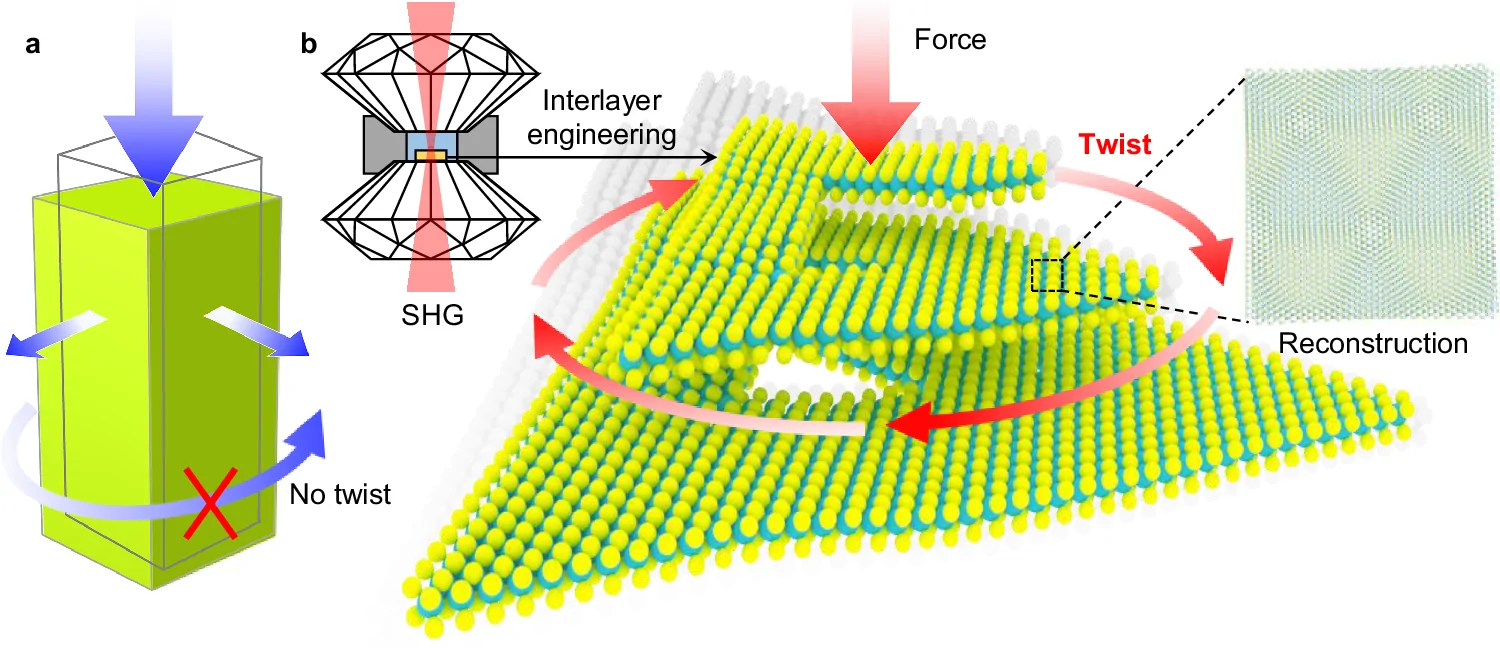
Twist degree of freedom in spiral WS_2_. **a** Conventional Cauchy continuum elasticity: axial compression produces lateral expansion (blue arrows) but no twist (red cross). **b** Force-twist coupling (red arrows) via interlayer engineering in spiral WS_2_, accompanied by atomic reconstruction of the moiré pattern and the resulting spontaneous interlayer torque. SHG denotes second-harmonic generation. The schematic was prepared using Blender.

Theoretical studies predict a direct link between moiré superlattice geometry and interlayer coupling^36,37^, and the large lateral extent of vdW flakes makes them particularly suitable for controlled out-of-plane loading rather than localized torsional actuation^38^. This naturally raises the question of whether one can bypass explicit torque and instead tune interlayer coupling to unlock the twist degree of freedom experimentally. However, realizing such force-twist coupling is nontrivial within Cauchy continuum mechanics, where homogeneous solids are described solely by displacement fields without independent rotational degrees of freedom (Fig. 1a). Accordingly, macroscopic compression-induced torsion has been realized mainly in mechanical metamaterials that embed 3D chiral architectures^39–43^. In these architected media, asymmetric ligaments and chiral unit cells transduce axial forces into global torsion, but the effect is primarily geometric design rather than intrinsic material properties and demands intricate fabrication and numerical optimization^39^, which limits downscaling and integration with atomically thin quantum systems.

Here we show that screw-dislocation-driven vdW spirals provide an intrinsic route to compression-twist coupling, as sketched in Fig. 1b. The spiral morphology and the built-in screw dislocation strain displace the lattice from the 3R ground state into a metastable parallel-oriented stacking that inherently carries spontaneous torque. This effectively preloads the stack with a latent rotational mode, even without an externally applied torsion. Under diamond-anvil-cell compression, enhanced interlayer coupling and moiré superlattice reconstruction rebalance vdW and elastic energies, allowing the latent spontaneous torque to drive interlayer rotation. Polarization-resolved second-harmonic generation (SHG) reveals a reversible rotation of the SHG polar pattern. This rotation corresponds to an effective average interlayer twist of about 0.2° per layer, equivalent to a model-derived top-to-bottom twist exceeding 6° per percent axial strain, comparable to or exceeding values reported for chiral metamaterials^39–42^. These findings identify spiral vdW crystals as a pressure-tunable 3D twistronic platform with addressable rotational degrees of freedom.

## Results

### Structural characterization of spiral WS_2_

**Fig. 2:**
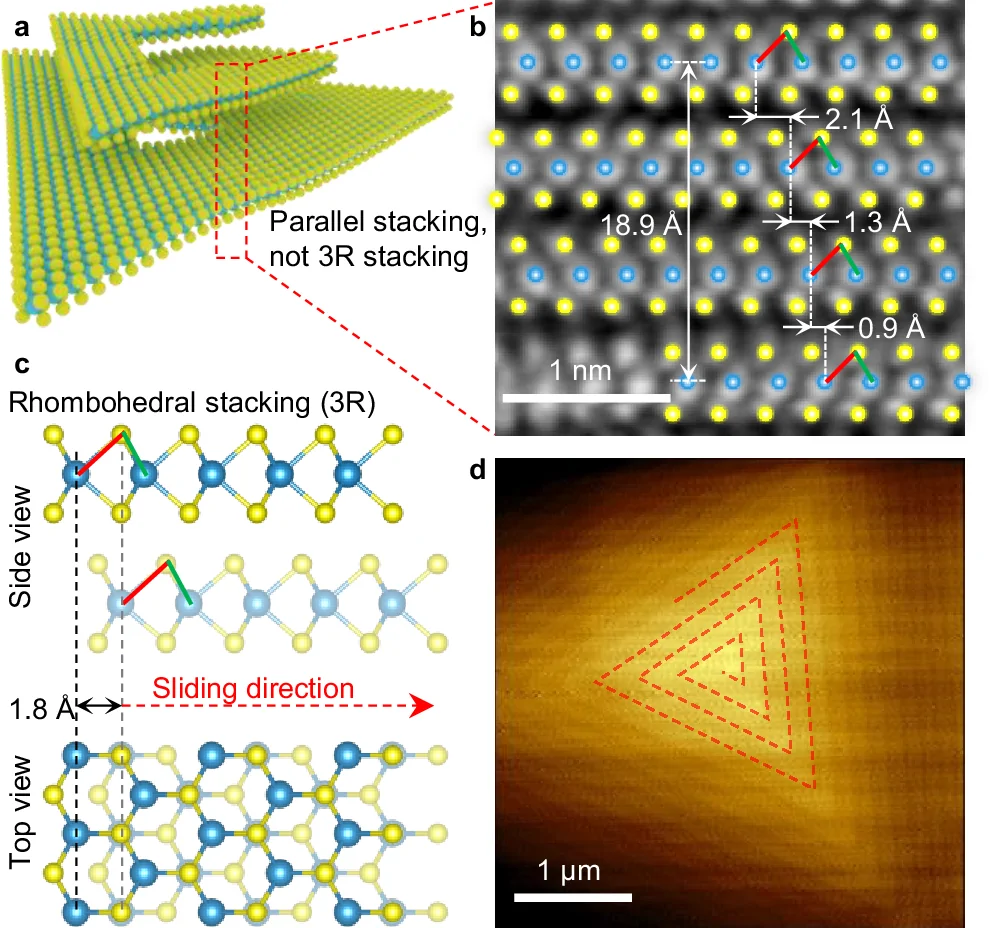
Interlayer stacking and in-plane sliding in screw-dislocation-driven spiral WS_2_. **a** Schematic of a screw-dislocation-driven spiral WS_2_ crystal, where the terraces wind around the dislocation core and adjacent layers are parallel. The schematic was prepared using Blender. **b** Cross-sectional scanning transmission electron microscopy (STEM) image with overlaid side-view atomic model. The sulfur (yellow) and tungsten (cyan) atoms show a parallel in-plane orientation across layers, as indicated by the red and green short lines, and reveal a translation-disordered, sterically expanded stacking motif that deviates from ideal 3R registry. Scale bar, 1 nm. **c** Side and top view models of rhombohedral (3R) WS_2_, illustrating the characteristic layer-to-layer in-plane shift along the armchair direction (1.8 Å). **d** Atomic force microscopy (AFM) image of a triangular spiral WS_2_, showing the dislocation-driven spiral trace (red dashed line). Scale bar, 1 μm.

Screw dislocations are line defects formed when part of a crystal is sheared by one lattice spacing relative to the rest. In layered crystals, such dislocations introduce an out-of-plane displacement that links successive layers and generates a spiral staircase morphology (Fig. 1a, b)^44–47^. In spiral WS_2_, screw-dislocation-controlled growth gives rise to a continuous sequence of spiral terraces, as evidenced by atomic force microscopy (AFM) images showing well defined concentric terraces winding around a central core (Fig. 2d and Supplementary Fig. 8). To resolve the stacking configuration at the atomic scale, we performed cross-sectional aberration-corrected scanning transmission electron microscopy (STEM) on spiral WS_2_ sliced along the armchair direction (Fig. 2b). In the side-view image, tungsten atoms maintain the same in-plane orientation from layer to layer, indicating parallel-oriented stacking rather than 60° rotated 2H sequence. This parallel-oriented stacking is consistent with the aligned spiral edges seen by AFM (Fig. 2d).

Despite the parallel-oriented stacking, the spiral WS_2_ does not follow the standard 3R sequence, in which adjacent layers are translated by 1.8 Å along the armchair direction to form a rhombohedral structure with a regular out-of-plane stacking period (Fig. 2c and Supplementary Fig. 9). Cross-sectional and plan-view STEM images (Fig. 2b and Supplementary Figs. 10, 11) instead reveal that the interlayer translations fluctuate from period to period, deviating substantially from this 1.8 Å shift and thereby destroying the regular 3R order. These irregular translations also tend to bring chalcogen atoms in adjacent layers into closer in-plane proximity, in contrast to 3R arrangement where sulfur atoms sit beneath the hexagon centers of the upper layer (Fig. 2c). The resulting steric repulsion expands the interlayer spacing to 6.29 Å (Fig. 2b), compared with 6.16 Å in bulk WS_2_^48,49^. The translation disorder and the sterically expanded spacing could be attributed to strain fields generated by the screw dislocation during growth^50,51^, which perturb the registry of newly deposited layers away from the energetically preferred 3R pattern (Supplementary Fig. 12). This registry provides the structural basis for the compression-twist coupling discussed below.

### Twist degree of freedom driven by interlayer engineering

**Fig. 3:**
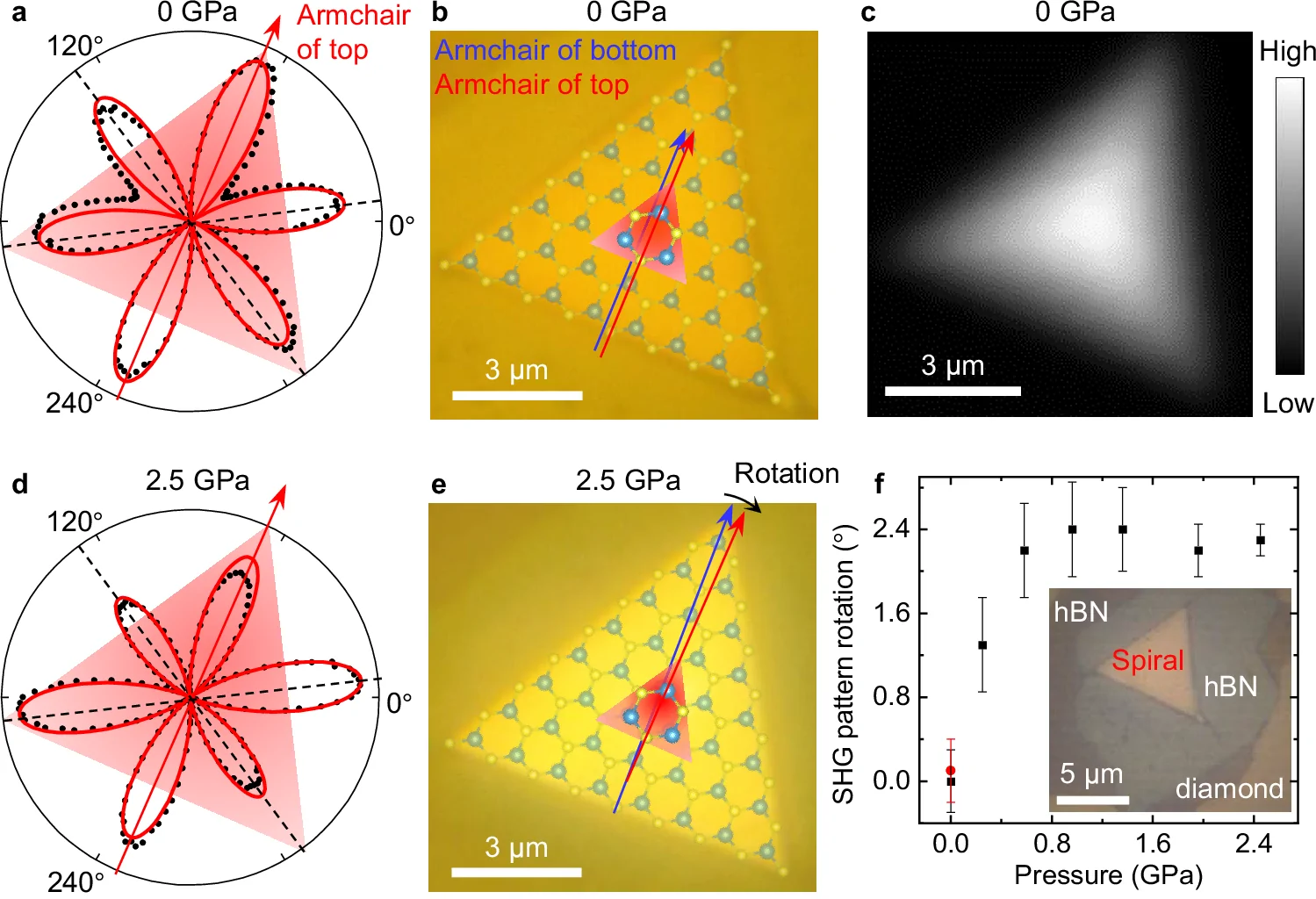
Pressure-controlled twist degree of freedom in spiral WS_2_ revealed by SHG pattern rotation. **a**,**d** Polarization-resolved SHG polar plots from the top surface at 0 GPa (**a**) and 2.5 GPa (**d**). Black symbols are experimental data, and red curves are fits based on the SH field superposition model. The fitted six-lobe pattern defines the armchair direction of the top surface (red arrow). **b**,**e** Optical images of the same spiral at 0 GPa (**b**) and 2.5 GPa (**e**), where the armchair direction of the bottom layers (blue arrow) is taken as perpendicular to the straight edges. Scale bars, 3 μm. **c** Spatial map of unpolarized SHG intensity at 0 GPa. Scale bar, 3 μm. **f** Rotation angle of the SHG polar pattern versus pressure for spiral WS_2_ (inset, optical image of the hBN-encapsulated flake on the diamond culet). The SHG pattern rotation reverses upon decompression (red data point). Error bars represent the fitting uncertainties of the SHG pattern rotation. Scale bar, 5 μm.

To directly track in situ interlayer reorientation under external stimuli, we use polarization-resolved SHG microscopy as an optical probe of crystal symmetry and in-plane orientation for noncentrosymmetric media^52,53^. At ambient pressure, the azimuthal polarization-resolved SHG pattern of spiral WS_2_ recorded from the top surface displays well-defined sixfold lobes whose maxima, obtained by fitting the polar plot (Fig. 3a and Supplementary Figs. 1, 2), define the armchair direction. This assignment is consistent with previous reports on monolayer and stacked transition metal dichalcogenides (TMDs) with in-plane threefold rotational symmetry^54–56^. For CVD-grown triangular TMD flakes, previous structural studies via TEM, STM, and ab initio calculations have established that straight edges correspond to zigzag directions, so the armchair direction lies perpendicular to these edges^52,54,57^. In spiral WS_2_, the armchair directions inferred for the bottom layers from the edge orientation (Fig. 3b) and extracted for the top surface from SHG polar fit (Fig. 3a) coincide within experimental uncertainty. This agreement indicates that, at ambient pressure, the in-plane crystallographic orientation is preserved from bottom to top across the multilayer spiral. Together with the strong and spatially uniform SHG intensity in the SHG map (Fig. 3c), as expected for parallel-oriented stacking, these observations provide an independent optical confirmation that the spiral WS_2_ adopts a parallel-oriented, noncentrosymmetric stacking configuration, consistent with the AFM and STEM analyses.

Classical Cauchy linear elasticity treats axial loading as essentially independent of rotational degrees of freedom^39,40^. By contrast, in spiral WS_2_, the translation-disordered and sterically expanded stacking identified above suggests that axial compression could relax the interlayer registry and activate an internal twist degree of freedom. To examine this potential compression-twist coupling, we encapsulated the spiral WS_2_ in hexagonal boron nitride (hBN) to isolate it from the pressure-transmitting medium (inset of Fig. 3f) and applied hydrostatic pressure while recording polarization-resolved SHG. As the pressure increases, the SHG polar pattern gradually rotates relative to the bottom-surface orientation defined by the triangular edges (Fig. 3d-f and Supplementary Fig. 2). The rotation reaches 2.5° around 1 GPa and then saturates (Fig. 3f). Upon decompression, the SHG pattern returns to its initial orientation (red point in Fig. 3f and Supplementary Fig. 2f), indicating the elastic and reversible compression-twist response.

In multilayer systems, the rotation of the SHG pattern does not directly equal the geometric twist angle of the entire stack, because the measured SHG field is a coherent superposition of layer-resolved SH dipoles. This response can be modeled as a weighted vector sum with layer-dependent attenuation factors accounting for absorption and refractive-index-induced phase mismatch (Supplementary Fig. 3)^56,58^. For a spiral WS_2_ with a thickness of about 20 nm (about 32 layers), this model maps a 2.5° SHG pattern rotation to an effective average twist of about 0.2° per layer. This corresponds to an overall twist of about 6° per percent axial strain, comparable to or exceeding values reported for engineered artificial chiral metamaterials (Supplementary Fig. 3)^39–42^. The twist direction follows the chirality of the screw dislocation (Supplementary Fig. 4): for the left-handed screw dislocation (Fig. 2d), axial compression tends to flatten the dislocation-induced out-of-plane displacement (helical step), which is mechanically equivalent to a clockwise rotation of the upper layers with respect to the bottom ones^59^. Together, these measurements show that enhanced interlayer coupling drives a finite, reversible interlayer twist, establishing a clear compression-twist coupling in the spiral WS_2_.

### High-frequency Raman response

**Fig. 4:**
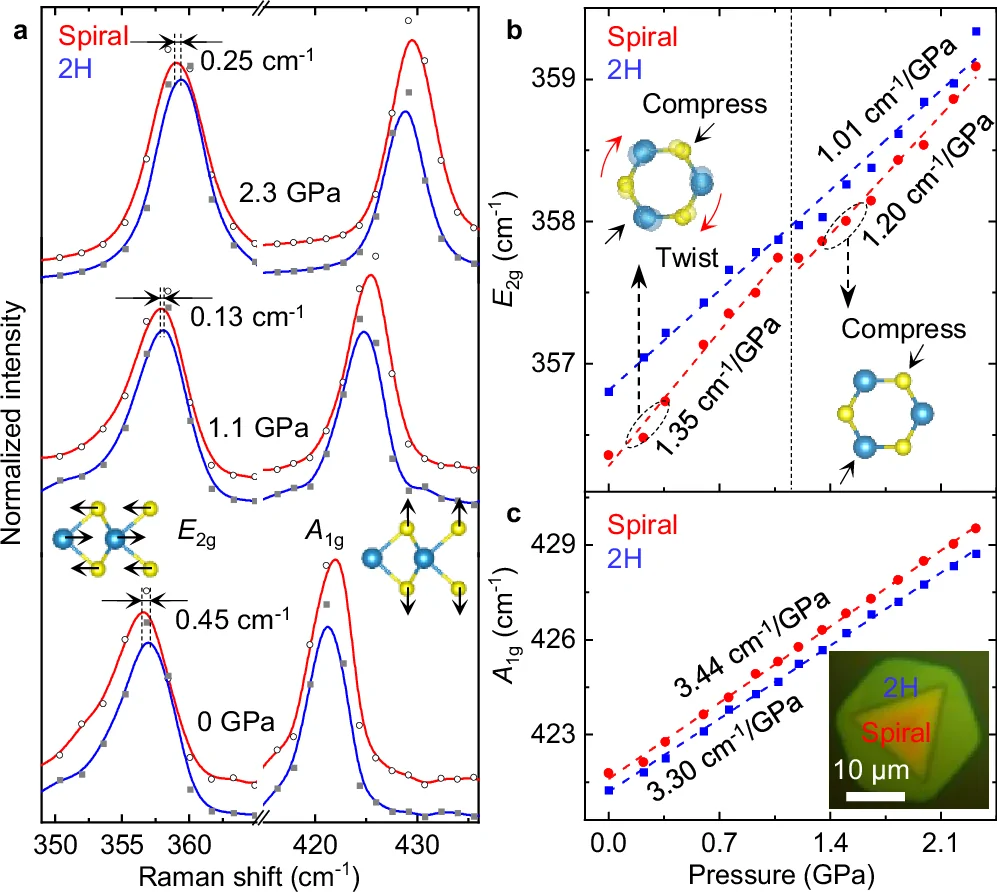
High-frequency Raman signatures of compression-twist coupling in spiral WS_2_. **a** Representative Raman spectra showing the in-plane *E*_2g_ and out-of-plane *A*_1g_ modes for the spiral (red) and 2H (blue) regions at selected pressures, acquired with 488 nm excitation. Symbols denote the experimental spectra, and the curves are B-spline guides to the eye. Inset, schematic atomic vibration patterns of the *E*_2g_ and *A*_1g_ modes. **b**,**c** Pressure dependence of the *E*_2g_ (**b**) and *A*_1g_ (**c**) mode frequencies for the spiral (red circles) and 2H (blue squares) regions. The dashed lines are linear fits to the indicated pressure ranges. Inset of (**b**), schematic attribution of the *E*_2g_ behavior to combined in-plane lattice compression and compression-induced interlayer twist. Inset of (**c**), optical image of a WS_2_ flake containing a central spiral domain and an adjacent 2H region.

Raman spectroscopy does not provide a direct quantitative measure of the interlayer twist angle in this multilayer, translation-disordered system. We therefore use it primarily as a sensitive probe of pressure-dependent phonon behavior and lattice dynamics, enabling a comparison between spiral and 2H-stacked WS_2_. Both samples were encapsulated in hBN and measured during the same DAC run under identical conditions (inset of Fig. 4c). The triangular spiral flake, exhibiting strong SHG response, was compared with the surrounding thick hexagonal flake, which served as the 2H-stacked reference for the Raman measurements (Supplementary Fig. 5). Raman spectra from both samples exhibit the dominant in-plane *E*_2g_ and out-of-plane *A*_1g_ modes characteristic of bulk WS_2_ (Fig. 4a). In both cases, the *A*_1g_ mode shows a larger pressure coefficient than the *E*_2g_ mode (Fig. 4b,c). This trend is expected for layered vdW crystals because the out-of-plane lattice spacing is more compressible than the in-plane lattice^38,60,61^.

By contrast, below 1 GPa, the *E*_2g_ mode in spiral WS_2_ hardens more rapidly with pressure than that in the 2H reference (Fig. 4b), with a pressure coefficient about 35% larger. This anomalous *E*_2g_ behavior is consistent with twist-induced moiré lattice reconstruction in the spiral structure (inset of Fig. 4b). At small twist angles (typically below 2°), large moiré supercells enable substantial atomic reconstruction into extended triangular stacking domains (XM/MX regions) separated by strain-soliton networks and localized high-energy nodes, as discussed below^12,13,62^. Such reconstruction introduces an additional in-plane strain component, estimated to be on the order of 0.18% at an interlayer twist of 0.2°^63^. This is comparable to the in-plane compression of about 0.22% expected under 1 GPa hydrostatic pressure (Supplementary Fig. 6)^48,49^. Below 1 GPa, the hydrostatic contribution and the evolving twist-driven reconstruction act together, steepening the *E*_2g_ pressure slope of the spiral sample. Above 1 GPa, the SHG-derived interlayer twist saturates, while the *E*_2g_ mode exhibits a reduced pressure coefficient, consistent with a diminished contribution from twist-related structural evolution.

Out-of-plane *A*_1g_ Raman shifts imply that pressure-induced interlayer translation is strongly constrained. The interlayer spacing in spiral WS_2_ is about 2% larger than in regular WS_2_ (Fig. 2b), comparable to the typical interlayer-spacing compression of about 1% per GPa (Supplementary Fig. 6)^48,49^. Thus, if substantial pressure-driven sliding toward a denser 3R registry occurred, it would be expected to introduce a detectable extra *A*_1g_ stiffening signature in spiral WS_2_ (for example, a noticeably larger slope or a clear anomaly). Instead, the *A*_1g_ slopes of spiral and regular WS_2_ differ only slightly across the entire pressure range (Fig. 4c), arguing against extensive interlayer sliding during compression. In-plane Raman data further indicate that compression-induced twist remains small. Previous studies have shown that twist angles of 2° to 6° increase the relative area of strain solitons, consistent with our calculation results in Supplementary Fig. 14, and can hence break the in-plane threefold rotational symmetry, splitting the doubly degenerate *E*_2g_ mode^63^. In spiral WS_2_, the absence of any resolvable *E*_2g_ splitting constrains the twist below about 2°, consistent with the small average twist inferred from the multilayer SHG analysis.

### Ultralow-frequency shear-like response

**Fig. 5:**
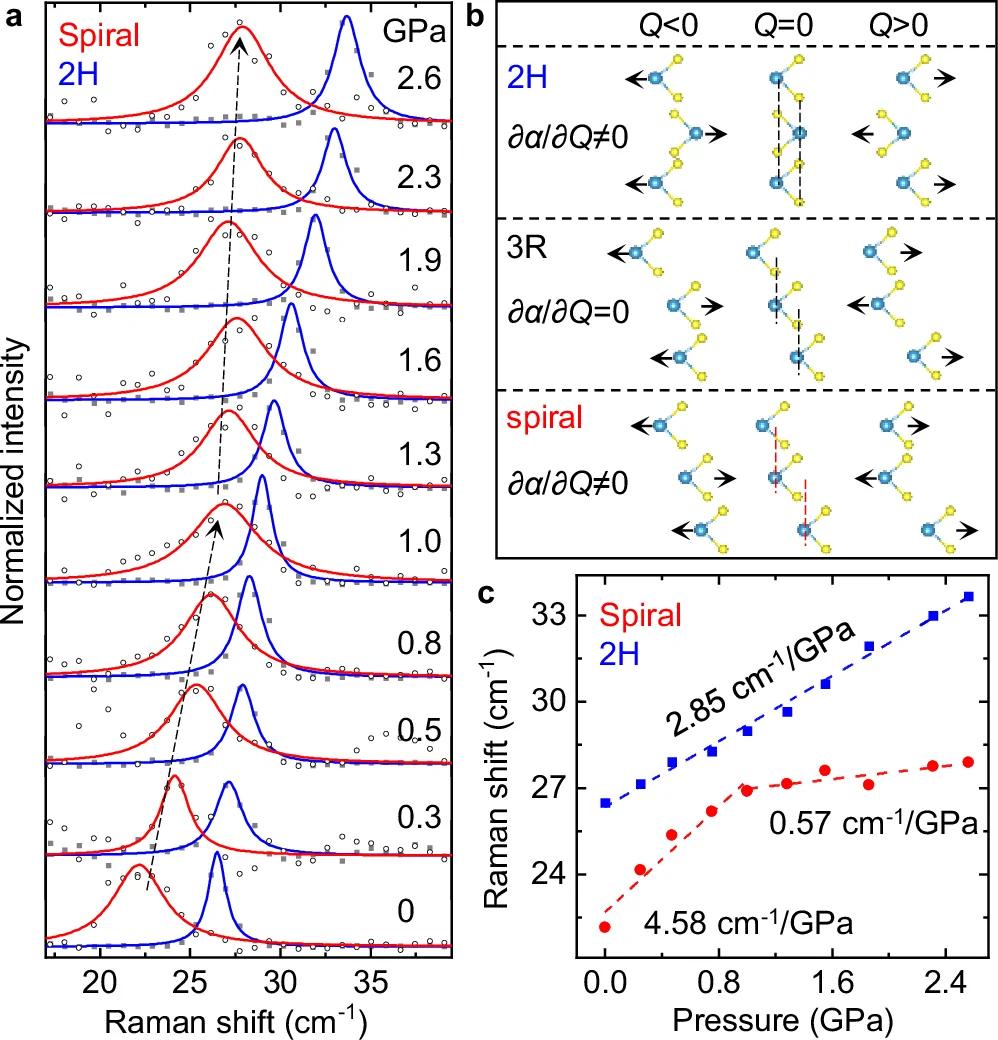
Ultralow-frequency Raman signatures of pressure-dependent interlayer shear-like coupling in spiral WS_2_. **a** Pressure-dependent ultralow-frequency Raman spectra of spiral WS_2_ (red) and 2H WS_2_ (blue) recorded with 442 nm excitation. The dashed lines trace the pressure evolution of the shear-like features. Symbols and solid curves denote the experimental spectra and Lorentzian-fitted profiles, respectively. **b** Schematic Raman-tensor response of the high-frequency shear branch under the normal coordinate *Q*. Random interlayer translations in spiral WS_2_ break the interfacial equivalence of ideal 3R stacking, yielding a finite polarizability derivative, ∂*α*/∂*Q* ≠ 0, and hence a Raman-active shear-like response. **c** Pressure-dependent shear-mode frequencies of spiral WS_2_ (red) and 2H WS_2_ (blue). The dashed lines are linear fits to the indicated pressure ranges.

Further insight into the compression-twist coupling is provided by ultralow-frequency Raman spectroscopy. At ambient pressure, spiral WS_2_ displays a weak and broad shear-like feature at 22.2 cm^-1^, redshifted relative to the high-frequency shear mode of the 2H reference at 26.5 cm^-1^ (Fig. 5a and Supplementary Fig. 19). We conservatively assign this feature to a disorder-activated high-frequency shear-like interlayer mode. In ideal 3R stacking, the equivalent interfaces yield nearly identical bond-polarizability derivatives, so that the interfacial contributions to the Raman tensor, *R*∝∂*α*/∂*Q*, cancel for the high-frequency shear branch (Fig. 5b). Here, *α* is the electronic polarizability tensor, and *Q* is the normal coordinate^64^. The opposite branch selectivity occurs in 2H stacking (Fig. 5b), where the alternating interface orientation permits coherent addition of the high-frequency shear mode contributions^64–66^. Spiral WS_2_ lies between these limits: its layers remain parallel-oriented, but layer-to-layer translations fluctuate away from the regular 3R sequence, rendering the interfacial bond-polarizability derivatives inequivalent (Fig. 5b). The incomplete cancellation therefore leaves a finite Raman response for the high-frequency branch. Its detection is further facilitated by the 442 nm excitation, which lies near the C-exciton resonance of WS_2_ and enhances the exciton-mediated Raman scattering amplitude^67,68^. Within the linear chain model, the bulk-like high-frequency shear branch obeys *ω*^2^ = *α*_∥_/(π^2^c^2^*μ*), where α_∥_ is the interlayer shear force constant, and μ is the monolayer areal mass density^60,68^. Treating the spiral feature as an effective high-frequency shear-like mode gives *α*_∥,spiral,eff_ = 2.09 ×10^19 ^N/m^3^, indicating a 30% reduction relative to 2H WS_2_ in the effective lateral restoring force. This reduction can be attributed to the expanded interlayer spacing, which reduces interlayer electronic overlap and weakens the lateral corrugation, as well as to random sliding and screw-dislocation-induced strain, which disrupt the equivalence of interlayer registries and reduce the long-range coherent restoring response^65,69^. Related stacking-dependent shear-mode softening and a reduced interfacial shear force constant have been reported for AB′-stacked MoSe_2_ bilayers and weakly coupled twisted multilayer graphene interfaces, respectively^68,69^. These interfacial shear-like phonon signatures provide a dynamical consequence of the slipped, metastable stacking that enables twist activation in spiral WS_2_.

The pressure evolution of this mode further links interlayer engineering to the twist degree of freedom. Below 1 GPa, the spiral feature hardens at 4.58 cm^-1^/GPa, compared with 2.85 cm^-1^/GPa for 2H (Fig. 5c). This enhancement coincides with the SHG pattern rotation and anomalous *E*_2g_ hardening (Figs. 3f and 4b), consistent with direct compression acting together with rotation-driven reconstruction to increase the effective lateral restoring force and hence the shear-mode blueshift^60^. Once the SHG rotation saturates near 1 GPa, this reconstruction-driven contribution is diminished, and the pressure coefficient of the shear-like mode falls to 0.57 cm^-1^/GPa (Fig. 5c). The markedly reduced post-crossover pressure coefficient does not indicate that every local interface ceases to stiffen. Instead, the broad shear-like feature is more appropriately viewed as a Raman-selected collective response; it combines unresolved contributions from reconstructed interfacial regions with different registry, local lateral stiffness, and Raman-tensor activity. Upon further compression, the reconstructed interfacial landscape may become locally more uniform, increasing the fraction of regions that exhibit near-3R-like cancellation of the high-frequency shear Raman tensor^64^. This enhanced cancellation could suppress the Raman weight of relatively stiff and higher-frequency components. The residual signal may then become increasingly weighted toward soliton-like or domain-boundary regions^63^, where the cancellation remains incomplete, and the local lateral response is comparatively compliant. A pressure-dependent redistribution among these unresolved components would partially limit the blueshift of the fitted peak centroid, even if local interlayer stiffness continues to increase throughout the multilayer. The Raman-selected shear-like feature can therefore harden much more slowly than the shear phonon in the laterally uniform 2H reference.

Taken together, the high- and ultralow-frequency Raman responses under pressure are consistent with compression-driven twist and interfacial reconstruction in the metastable spiral stacking, providing complementary vibrational support for the proposed twist degree-of-freedom mechanism.

### Moiré-driven origin of compression-twist coupling

Within Cauchy linear elasticity, the stress σᵢⱼ is related to the symmetric strain *ε*_*kl*_ via *σ*_*ij*_ *= C*_*ijkl*_ *ε*_*kl*_. Because *C*_*ijkl*_ is invariant under space inversion, this homogeneous description contains no independent internal rotational degree of freedom under pure compression^40^. Micropolar elasticity extends this framework by introducing microrotation and couple-stress fields, allowing such coupling in micropolar media through additional force-rotation terms (Supplementary Fig. 15)^40–42^. For spiral WS_2_, we use this framework only as a conceptual effective-medium analogy. The translation-disordered interfaces can be viewed as an effective micropolar interfacial medium, introducing local registry and relative-rotation coordinates that are absent from a homogeneous Cauchy description (Supplementary Fig. 15). At the microscopic level, we propose that compression activates this rotational degree of freedom by modifying the moiré energy landscape through atomic reconstruction, thereby generating an interlayer rotational torque. Spiral WS₂ therefore provides an atomic-scale route to pressure-tunable compression-twist coupling through interfacial engineering.

**Fig. 6:**
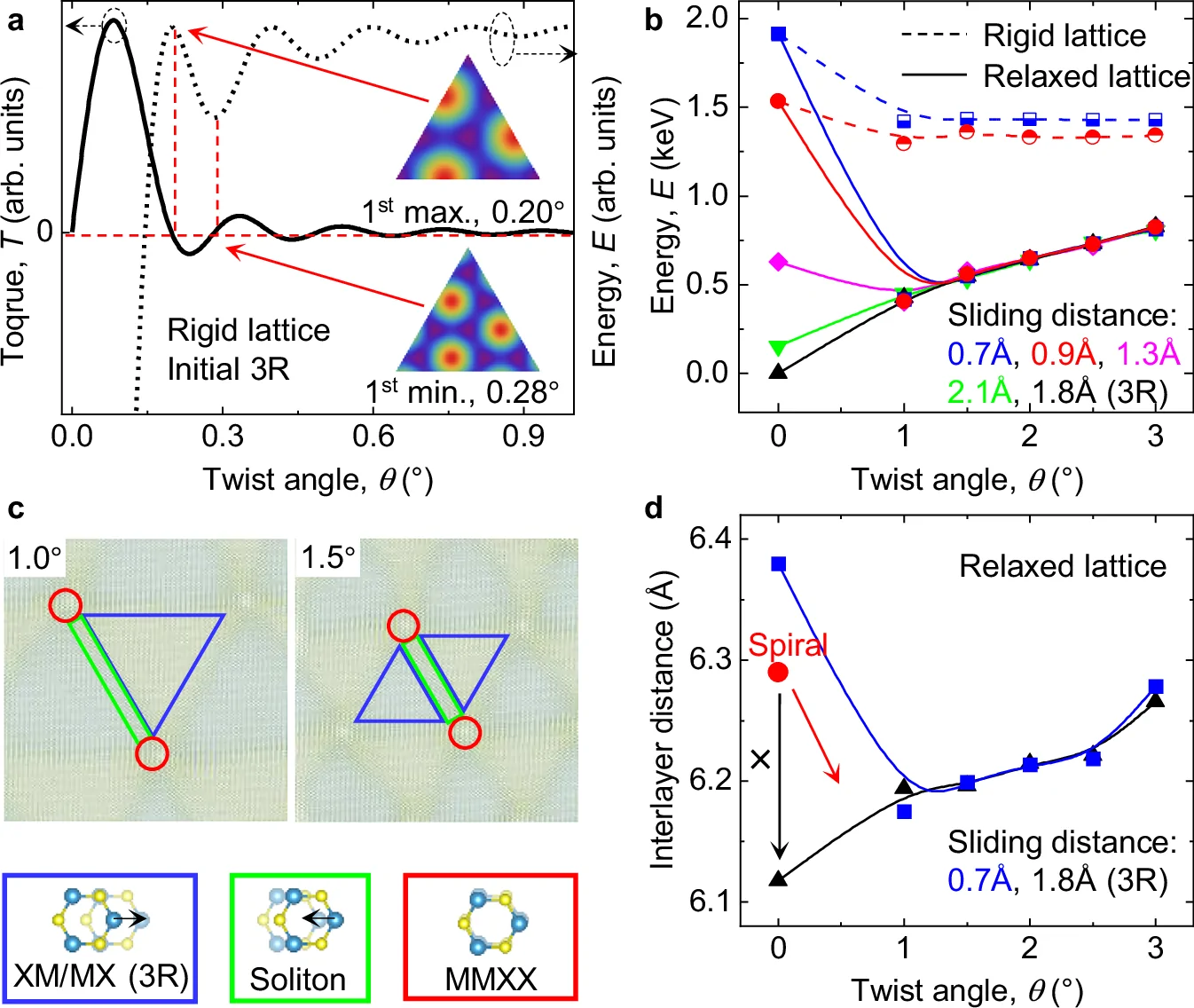
Moiré-governed spontaneous torque and reconstruction enabled coupling among twist, lateral sliding, and interlayer spacing in spiral WS_2_. **a** Calculated rotational energy *E*(*θ*) (dashed) and interlayer torque *T*(*θ*)∝d*E*/d*θ* (solid) for a rigid triangular bilayer WS_2_ initialized in 3R stacking (side length *L* = 160 nm), providing a thermodynamic driving force for interlayer rotation. Insets, local stacking energy maps at the first maximum (around 0.20°) and the first minimum (around 0.28°). **b** Total energy of bilayer WS_2_ versus twist angle for different in-plane sliding distances (0.7 Å, 0.9 Å, 1.3 Å, 1.8 Å, and 2.1 Å, where 1.8 Å corresponds to ideal 3R stacking) for rigid (dashed) and relaxed (solid) lattices. **c** Relaxed-lattice moiré reconstructions at 1.0° and 1.5°, with representative local atomic stacking configurations: 3R-like XM/MX domains (blue), soliton regions (green), and MMXX nodes (red). **d** Interlayer distance versus twist angle with ideal 3R stacking (black triangles) and a representative slipped registry (blue squares, sliding distance of 0.7 Å), together with the experimental interlayer distance of the spiral sample (red circle). Solid arrows are guides to the eye. In (**b**) and (**d**), symbols denote discrete calculated values, and the curves are B-spline guides to the eye.

To clarify the microscopic driving forces for interlayer rotation, we employ a moiré-geometry-based model to compute the twist-angle-dependent stacking-energy landscape and the resulting rotational torque for triangular WS_2_ bilayer initialized in 3R registry (Fig. 6a and Supplementary Fig. 7)^37,70,71^. The calculated stacking energy forms a periodic moiré pattern with alternating high-energy (red) and low-energy (purple) domains, and its angle-dependent morphology sets the rotational energy profile *E*(*θ*). At 0.20°, a triangular flake with side length 160 nm contains a larger high-energy area than at 0.28°, giving a higher integrated stacking energy (inset of Fig. 6a). The interlayer torque follows from this angle-dependent energy landscape, *T*(*θ*)∝ d*E*/d*θ*, so it vanishes at energy extrema and drives relaxation of the twist angle toward nearby minima when the system is perturbed (Fig. 6a). These energy extrema thus define rotational barriers, so interlayer rotation naturally stabilizes once a local minimum is reached, accounting for the saturation of pressure-driven twist angle observed experimentally (Fig. 3f).

Within this moiré driven rotational energy landscape, perfect 3R stacking at *θ* = 0° corresponds to the global energy minimum, so small angular perturbation experiences a restoring torque that relaxes the twist back to *θ* = 0° rather than enabling an internal twist degree of freedom (Fig. 6a and Supplementary Fig. 7). Spiral WS_2_ instead shows translation-disordered, sterically expanded stacking (Fig. 2). Motivated by this structure, we introduce an in-plane interlayer slip along the armchair direction to reshape the rotational energy landscape. Atomic level simulations of the total energy as a function of twist angle and in-plane shift (Fig. 6b) show that even modest slip (such as 1.3 Å) lowers the rotational barrier, because twisting no longer forces the system to pass through the high-symmetry 3R registry^27^. In the relaxed-lattice model, increasing the slip to 0.7 Å or 0.9 Å further shifts the global minimum away from *θ* = 0° and creates a minimum at a finite twist angle (Fig. 6b). Calculations on twisted trilayer WS_2_ containing two coupled interfaces show a consistent finite-angle stabilization (Supplementary Figs. 16–18). Together, these results provide a thermodynamic driving force for spontaneous rotation.

Building on this slipped, metastable configuration, we next examine how moiré reconstruction couples the twist angle *θ* to the interlayer spacing. In a relaxed-lattice picture at large *θ* above 6°, or in an idealized rigid-lattice, the average interlayer spacing is nearly independent of *θ*^36,37,63^. This behavior changes in the small-angle reconstructed regime. At small angles, the competition between vdW interaction energy and elastic strain at the interface drives atomic reconstruction into a domain network with stacking-dependent relaxed spacings (Fig. 6c and Supplementary Figs. 13, 14). For example, as *θ* decreases from 1.5° to 1.0° (Fig. 6c), low-energy XM/MX domains expand while high-energy XXMM domains shrink. This redistribution weakens steric repulsion and thereby reduces both the total energy and the average interlayer spacing (Fig. 6b, d)^12,13,36^. The slipped interface therefore differs fundamentally from ground-state 3R registry^36^: finite twisting can lower, rather than raise, the relaxed interlayer spacing (Fig. 6d).

Under compression, this difference creates a competition between translation and rotation. Relative to the ideal 3R stacking, the slipped, metastable spiral WS_2_ has two possible relaxation channels under compression: lateral sliding toward the smaller spacing 3R registry or twisting to rearrange the moiré stacking domains. Experimentally, as discussed above (Fig. 4c), the out-of-plane *A*_1g_ response rules out extensive pressure-induced interlayer sliding in the spiral crystal, marked by the black arrow and cross in Fig. 6d. Consistently, theory for multilayer moiré systems indicates that twist alignment can yield a larger energetic gain than pure translation^72^. In spiral WS_2_, the 3D screw-dislocation strain field should further penalize large-scale lateral sliding. Together, these constraints favor rotation away from the initial *θ* = 0° state toward a small finite twist angle rather than lateral sliding (red arrow in Fig. 6d). This behavior is consistent with the calculated 0.7 Å slipped configuration shown in Fig. 6d. The twist is accompanied by a reconstructed stacking pattern that supports a reduced interlayer spacing compatible with the compressed state, allowing the system to relax into a lower-energy minimum.

## Discussion

Beyond this thermodynamic driving force set by the rotational energy landscape, the kinetics of compression-twist coupling are controlled by interfacial friction. The incommensurate, slipped interface is expected to be in a structural superlubricity regime with very low lateral friction, as established in twisted graphite and other vdW heterostructures^72–75^. Under load, the out-of-plane moiré corrugation is progressively flattened, which should suppress energy dissipation through out-of-plane modes and further lower effective friction^76,77^. These conditions provide favorable kinetics for the moiré-driven rotational torque to realize compression induced twist. Upon unloading, the interlayer spacing relaxes back toward its initial value, so the same low-friction interface allows the spiral WS_2_ to untwist and recover its original orientation (Fig. 3f). This reversibility parallels the self-retracting motion of graphite flakes observed in classic superlubric torque experiments^78,79^.

In conclusion, we demonstrate that screw-dislocation-driven spiral WS_2_ hosts an intrinsic, pressure-tunable twist degree of freedom. Hydrostatic compression alone, without external torque actuators or artificial metamaterials, induces a small but finite and reversible interlayer rotation. Structural characterization reveals a translation disordered, sterically expanded stacking that places the interface in a slipped metastable state. Moiré-based modeling further indicates that atomic reconstruction reshapes the stacking energy landscape and generates a spontaneous rotational torque that couples the twist to interlayer spacing. Beyond establishing compression-twist coupling in spiral WS_2_, our results suggest opportunities for twistronics and moiré engineering. A pressure-tunable internal twist in three-dimensional moiré crystals provides a route to reconfigurable higher-order moiré architectures and dynamic access to correlated and topological phases. More broadly, coupling axial strain to interfacial rotation enriches vdW mechanics beyond classical Cauchy elasticity and provides a solid-state platform to explore chiral elastic responses. This coupling also points to mechanically controlled twistronic elements that link mechanical loading to electronic and optical functions in future NEMS or MEMS devices.

## Methods

### Synthesis of Spiral WS_2_ nanostructures

Spiral WS_2_ nanostructures were synthesized using a previously reported water-vapor-assisted chemical vapor transport growth^80^. A custom-built reactor system, consisting of a 1-inch outer diameter fused silica tube, pressure and gas flow controls, a Lindberg Blue M three-zone tube furnace, and two heating tapes, was used as the reactor. 100 mg WS_2_ precursor powder (Alfa Aesar) was placed in the first zone of the three-zone furnace, and the SiO_2_/Si substrate was placed between the second and the third zone with the polished side facing up. 1 g CaSO_4_‧2H_2_O powder (Sigma Aldrich) was placed upstream from the heating zone as the water vapor source and heated with two heating tapes. With 100 sccm argon flowing at 800 torr, the second zone was heated to 1200 °C at a rate of 20 °C/min, and simultaneously the third zone was heated to 700 °C at the same rate, while the CaSO_4_‧2H_2_O was not heated. Once the furnace temperatures were reached, the CaSO_4_‧2H_2_O was heated to around 90 °C to release the water vapor. After all the temperatures were stabilized, the WS_2_ boat was pushed into the second zone by a magnet coupled positioner and a quartz rod to initiate the reaction. After 15 min reaction, the furnace was opened and cooled down rapidly.

### Polarization resolved SHG patterns

SHG measurements were performed using a Ti:Sapphire femtosecond laser with a wavelength of ~800 nm, a pulse width of ~100 fs, and a repetition rate of 80 MHz. The laser beam was mechanically chopped for signal demodulation to suppress background noise. The polarization of the input laser beam was controlled by a half wave plate and focused onto the sample through a 50× long working distance objective lens. Under this optical configuration, the SHG excitation spot diameter was calibrated to be 1.5–2 μm. The generated SHG signal at ~400 nm was collected by the same objective lens and separated from the fundamental beam by a harmonic separator. A polarizer selected the polarization of the SHG signal, and color filters were used to block the fundamental laser. The SHG signal was detected by a photomultiplier tube and demodulated at the modulation frequency of the input laser beam using a lockin amplifier. Control SHG measurements confirmed that the diamond anvils, silicone oil, and hBN encapsulation do not affect the polarization-resolved SHG patterns under our measurement conditions (Supplementary Figs. S20, 21).

### High-frequency Raman and AFM characterization

High-frequency Raman spectra were measured using a Raman spectrometer (Renishaw Inc.) with a 488 nm laser as the excitation source. Sample morphology was characterized by an AFM system (Vistascope, Molecular Vista) operated in tapping mode.

### Ultralow-frequency Raman characterization

Ultralow-frequency Raman spectra were measured under a backscattering configuration at 300 K with a Jobin Yvon HR800 Raman system equipped with a liquid-nitrogen-cooled charge-coupled device (CCD) and a 50× objective (numerical aperture = 0.35). 1800 lines per mm grating was used in the Raman measurements. The excitation energy was 2.81 eV from a HeCd laser. The plasma lines were removed by using BragGrate Bandpass filters. Measurements down to 5 cm^-1^ were enabled by three BragGrate notch filters (OptiGrate Corp) with an optical density of 4 and full width at half maximum of ∼6 cm^-1^ and 10 cm^-1^, respectively.

### Cross-sectional aberration-corrected STEM characterization

Cross sectional lamellae of the spiral WS_2_ were first prepared along the armchair direction by focused ion beam milling and thinning. The iDPC-STEM imaging was conducted in a Cs-corrected STEM (FEI Titan Cubed Themis G2 300) operated at 300 kV. The convergence semi-angle was 25 mrad and the collection angle was 6-34 mrad.

### High-pressure measurements in the diamond anvil cell

The high-pressure measurements were performed in a diamond anvil cell composed of two diamond anvils with 800 μm culets and a 304 stainless-steel gasket of approximately 30 μm thickness. Silicone oil was used as the pressure-transmitting medium, and pressure was calibrated using the standard ruby-luminescence method.

### Atomic level simulations

Empirical potentials were adopted, with slightly modified Stillinger-Weber and Lennard-Jones (LJ) parameters^81,82^. These parameters were implemented in the QuantumATK package for structural relaxation and total energy calculations. Twisted bilayer and trilayer WS_2_ models were constructed in supercells of approximately 490 Å × 490 Å × 35 Å, containing about 110,286 and 160,065 atoms, respectively.

## Supplementary information

Supplementary Information

Transparent Peer Review file

## Source data

Source Data

## Data Availability

The Source Data underlying the figures of this study are available with the paper. All raw data generated during the current study are available from the corresponding authors upon request. Source data are provided with this paper.
