# Supplementary material for: Twist degree of freedom activated by pressure-driven interlayer engineering in spiral WS2
Source: Nat Commun. 2026 Sep 16;17:9928. doi: 10.1038/s41467-026-77227-3 (PMC13586321; doi:10.1038/s41467-026-77227-3)
Supplement: Supplementary file 1 [file 41467_2026_77227_MOESM1_ESM.pdf]

Supplementary Information

Twist degree of freedom activated by pressure-driven interlayer engineering in spiral WS_2

Penghong Ci[1,†,*], Muhua Sun[2,†], Yabin Chen[3,†], Yoonsoo Rho[4,5,†], Yuzhou Zhao[6], Kaiyao Xin[1], Zifeng Mai[1], Shanyu Liang[1], Zhongjie Wang[1], Jiangbin Wu[1], Hao Hong[7], Zhongming Wei[1], Costas P. Grigoropoulos[5], Yue-Yang Liu[1,*], Ping-Heng Tan[1], Junqiao Wu[8,*]

[1]State Key Laboratory of Semiconductor Physics and Chip Technologies, Institute of Semiconductors, Chinese Academy of Sciences, Beijing 100083, China
[2]Key Laboratory of Advanced Materials (MOE), School of Materials Science and Engineering, Tsinghua University, Beijing 100084, China
[3]School of Interdisciplinary Science, Beijing Institute of Technology, Beijing 100081, China
[4]Department of Mechanical Engineering, Ulsan National Institute of Science and Technology (UNIST), Ulsan, 44919, Republic of Korea
[5]Department of Mechanical Engineering, University of California, Berkeley, CA 94720, USA
[6]School of Physical Science and Technology, ShanghaiTech University, Shanghai, 201210, China
[7]State Key Lab for Mesoscopic Physics and Frontiers Science Center for Nano-optoelectronics, School of Physics, Peking University, Beijing, China
[8]Department of Materials Science and Engineering, University of California, Berkeley, CA 94720, USA

These authors contributed equally: Penghong Ci, Muhua Sun, Yabin Chen, Yoonsoo Rho.
E-mail: ci@semi.ac.cn, yueyangliu@semi.ac.cn, wuj@berkeley.edu

List of contents:

Supplementary Figure 1. Second harmonic generation (SHG) spectrum of the spiral WS_2 under 800 nm excitation.

Supplementary Figure 2. Polarization resolved SHG analysis for extracting the armchair orientation of the top surface and its rotation relative to the bottom lattice in spiral WS_2 under pressure.

Supplementary Figure 3. SH field superposition analysis of a multilayer spiral WS_2 flake for extracting the average interlayer twist per layer.

Supplementary Figure 4. Schematic illustration showing how the chirality of a left-handed screw dislocation determines the clockwise twisting tendency of a spiral structure under compression.

Supplementary Figure 5. SHG characterization of the spiral/2H WS_2 flake used for Raman measurements.

Supplementary Figure 6. Comparison between twist induced reconstruction strain and hydrostatic pressure induced lattice compression in layered TMD crystals.

Supplementary Figure 7. Rotational energy landscape of a rigid triangular bilayer WS_2 with initial 3R stacking.

Supplementary Figure 8. AFM topography of the spiral WS_2 crystal.

Supplementary Figure 9. Plan-view atomic-resolution HAADF-STEM reference of ideal 3R stacking.

Supplementary Figure 10. Plan-view HAADF-STEM evidence for spatially varying local registry in spiral WS_2.

Supplementary Figure 11. Focal-series HAADF-STEM of multilayer spiral WS_2.

Supplementary Figure 12. Strain-dependent energy and interlayer spacing of rigid slipped bilayer WS_2 configurations.

Supplementary Figure 13. Moiré supercells of twisted bilayer WS_2 at small twist angles (θ = 1.0°, 1.5°, and 2.0°).

Supplementary Figure 14. Moiré supercells of twisted bilayer WS_2 at intermediate twist angles (θ = 2.5° and 3.0°).

Supplementary Figure 15. Schematic comparison between Cauchy and micropolar descriptions of compression-twist coupling.

Supplementary Figure 16. STEM-constrained helical trilayer model of spiral WS_2.

Supplementary Figure 17. Moiré supercells of twisted trilayer WS_2 at small twist angles (θ = 1.0°, 1.5°, and 2.0°).

Supplementary Figure 18. Moiré supercells of twisted trilayer WS_2 at intermediate twist angles (θ = 2.5° and 3.0°).

Supplementary Figure 19. Ultralow-frequency Raman signatures of pressure-dependent

interlayer shear coupling in spiral WS_2.

Supplementary Figure 20. Pressure-dependent polarization-resolved SHG response of a regular 3R-stacked TMD reference.

Supplementary Figure 21. High-pressure SHG control of bulk 2H and 3R MoS_2 with diamond-background measurements.

Supplementary Note 1. Atomic-level simulation details.

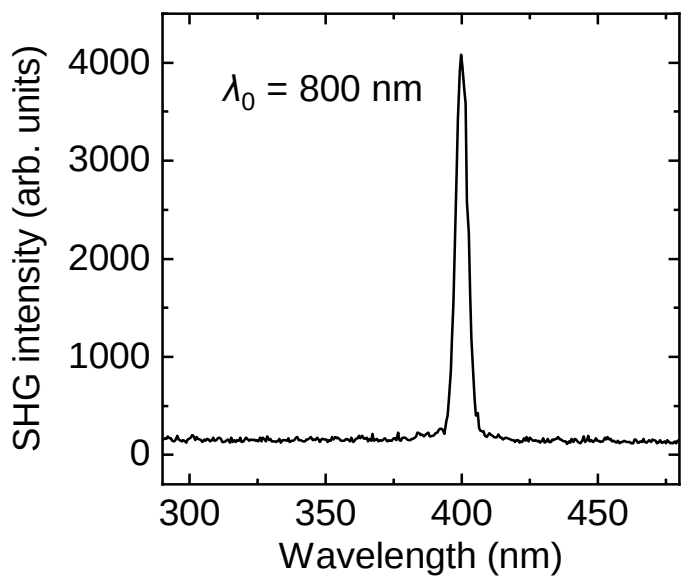

Supplementary Figure 1. Second harmonic generation (SHG) spectrum of the spiral WS_2 under 800 nm excitation.

SHG intensity as a function of emission wavelength for a spiral WS_2 pumped with a fundamental laser (ω) at λ_0 = 800 nm, showing a sharp peak at 400 nm corresponding to the second harmonic (2ω) of the excitation beam.

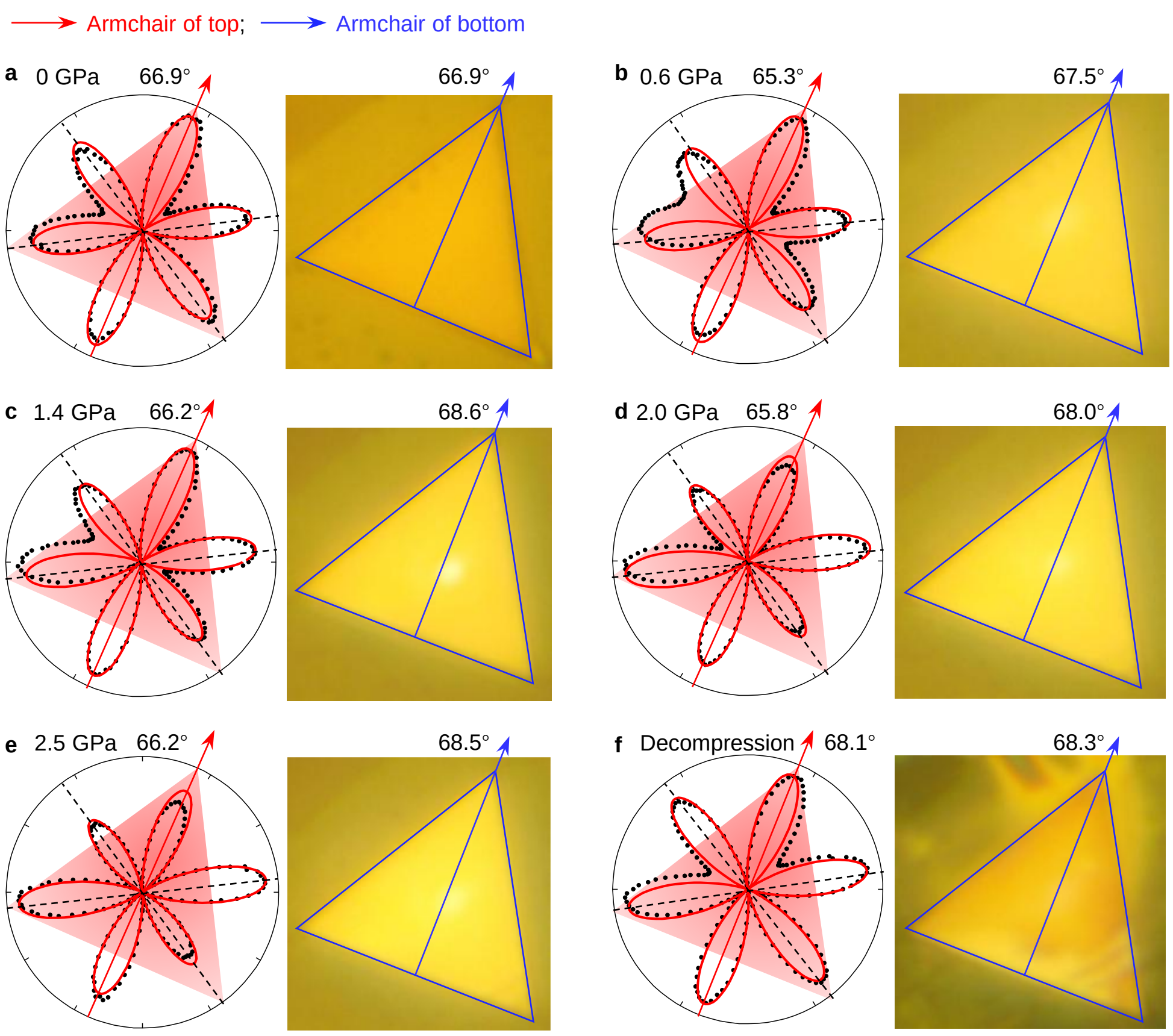

	A	B	η (°)	Armchair of top, ϕ (°)
**0 GPa**	1.9	-0.084	89.4	66.9
**0.6 GPa**	4.1	-0.27	86.0	65.3
**1.4 GPa**	2.4	-0.16	68.8	66.2
**2.0 GPa**	3.2	-0.26	51.0	65.8
**2.5 GPa**	2.6	-0.25	49.3	66.2
**Decompression**	1.9	-0.030	68.8	68.1

Supplementary Figure 2. Polarization resolved SHG analysis for extracting the armchair orientation of the top surface and its rotation relative to the bottom lattice in spiral WS_2 under pressure.

a-f, For each pressure point, the left panel shows the measured polarization resolved SHG pattern of the top surface (black symbols) together with a fit using a strain dependent SHG model adapted from Mennel et al. (red line)[1], and the fitted armchair angle ϕ of the top surface (which lies close to, but not exactly at, the SHG intensity maximum under finite strain) is indicated by the red arrow. The right panel shows the corresponding optical image of the triangular spiral flake, where the armchair direction of the bottom lattice (blue arrow) is taken to be perpendicular to the straight edges of the triangle (zigzag edges), as described in the main text. Comparison between the red and blue arrows as pressure increases from 0 GPa to 2.5 GPa and after decompression visualizes the pressure induced rotation of the top layer relative to the bottom lattice and its partial reversibility.

g, Fitted parameters A, B, η, and ϕ for the data in (a)-(f), obtained from the polarization resolved

SHG patterns using the model described below; here ϕ corresponds to the armchair angle of the top surface relative to the horizontal direction in the laboratory frame, which is listed in the last column.

The fitting expression used for Supplementary Fig. 2 builds on the strain dependent SHG theory developed by Mennel et al. for non-centrosymmetric transition metal dichalcogenide (TMD) semiconductors[1,2]. Upon normal incidence, when the excitation polarizer is parallel to the SHG signal analyzer, the detected SHG intensity as a function of the laboratory polarization angle φ can be written as

$$I_{\parallel}{}^{2}(\varphi) \propto [A\cos(3(\varphi - \phi)) + B\cos(2\eta + (\varphi - \phi))]^{2}$$

Here φ is the angle of the incident linear polarization measured from the horizontal axis of the optical image, and ϕ is the orientation of the crystal armchair axis with respect to the same axis, so that (φ - ϕ) gives the polarization angle in the crystal frame. η denotes the principal strain direction, and A and B are combinations of the photoelastic coefficients (p_1, p_2), the principal strains (ε_{xx}, ε_{yy}), the Poisson ratio (ν), and the unstrained nonlinear susceptibility (χ_0), where $A = (1-\nu)(p_1 + p_2)(\varepsilon_{xx} + \varepsilon_{yy}) + 2\chi_0$ and $B = (1+\nu)(p_1 - p_2)(\varepsilon_{xx} - \varepsilon_{yy})$. In our work we are mainly interested in crystal orientation: for each pressure we record the SHG polar pattern of the spiral top surface, fit it with the above expression, and take the fitted armchair angle ϕ that maximizes $I(\varphi)$ as the armchair direction of the top layer in the laboratory frame.

Note: Once a finite twist develops, the detected SHG response arises from the superposition of signals from the top layers rather than from a single monolayer. Consequently, the "armchair of top" extracted from the fit should be regarded as an effective armchair orientation of this multilayer stack. Later in the Supporting Information, we will use SH field superposition theory to establish the relationship between total rotation of the SHG pattern and the average twist angle per layer under the assumption of a uniformly twisted stack.

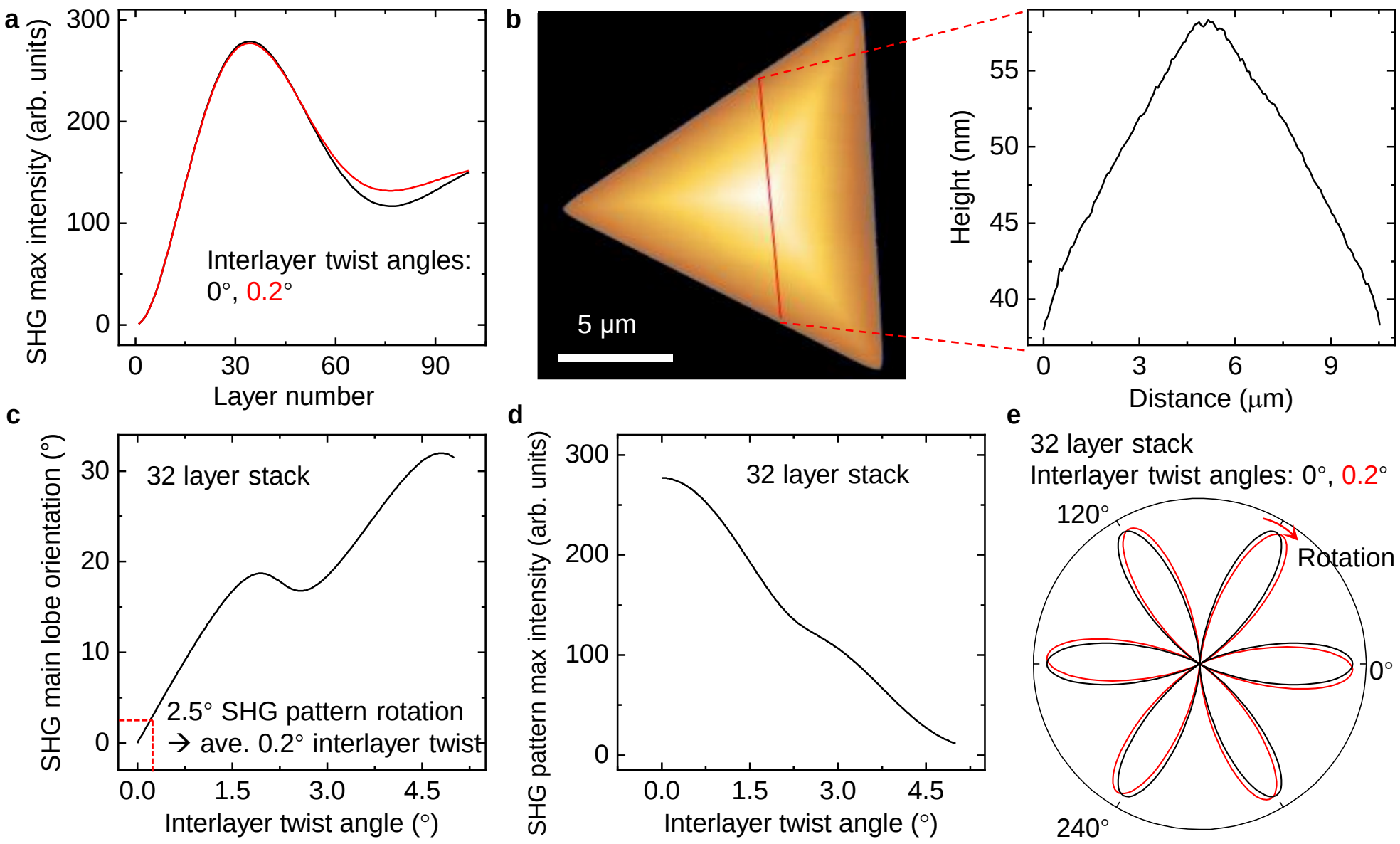

Supplementary Figure 3. SH field superposition analysis of a multilayer spiral WS_2 flake for extracting the average interlayer twist per layer.

a, Calculated SHG pattern maximum intensity as a function of layer number for two different interlayer twist angles, 0° (black) and 0.2° (red), using the SH field superposition model that incorporates both absorption and phase mismatch[2,3]. The nonmonotonic dependence of the peak intensity on thickness is consistent with previous reports on multilayer TMDs[3,4].

b, Atomic force microscopy (AFM) image of the spiral WS_2 flake, with the red line indicating the position of the height profile shown on the right. The thickness is about 20 nm, corresponding to roughly 32 layers. Scale bar, 5 μm.

c, For a stack of 32 layers, calculated orientation of the main SHG lobe as a function of the interlayer twist angle. The red dashed lines mark the experimentally observed rotation of the SHG pattern by about 2.5°, which corresponds to an average interlayer twist of about 0.2° per layer under the uniformly twisted stack assumption. Thus, we obtain an overall twist angle change of about 6° at 1 GPa; combined with the out-of-plane strain of about 1% at 1 GPa (Supplementary Fig. 6b), this corresponds to a twist strain susceptibility of roughly 6° per percent axial strain, comparable to or even larger than values reported for existing chiral metamaterials[5-8].

d, Calculated maximum SHG intensity as a function of interlayer twist angle for the same 32 layer stack, showing a gradual decrease of the SH response as the interlayer twist grows, reflecting the loss of constructive SH field superposition that occurs at zero twist angle.

e, Simulated SHG polar patterns for a 32 layer stack without interlayer twist (black) and with an average interlayer twist of 0.2° per layer (red), illustrating a net rotation of the six lobe pattern of 2.5° that matches the experimental SHG rotation used in the main text.

SH field superposition theory for multilayer TMDs:

In multilayer TMDs, the observed SHG response arises from the coherent sum of contributions from individual layers. For a stack of electrically decoupled layers with a twisting angle between them, the SH field from each layer behaves like a dipole whose phase and amplitude are modified by propagation through the flake[2,3,9]. For bilayers, if absorption and phase mismatch are neglected, the total SH field ($\vec{E}^{T}_{2\omega}$) is the vector sum of the two layer contributions and can be written as $\vec{E}^{T}_{2\omega} = \vec{E}^{1}_{2\omega} + \vec{E}^{2}_{2\omega} \propto cos3\phi + cos3(\phi + \theta)$, where ϕ is the azimuthal angle between the incident polarization and the armchair axis and θ is the relative twist angle between the two layers[9]. This simple case already explains why 2H stacking (θ = 60°) suppresses SHG whereas 3R like stacking (θ = 0°) leads to constructive interference and enhanced intensity.

To describe a general N layer stack including optical losses, the SH field from layer n is written as $\vec{E}^{N}_{2\omega} \propto \Gamma_N \cos\, 3(\phi - \delta_N)$ [3]. Here δ_N is the twisting angle of layer *N* relative to a reference layer, and Γ_N is a complex attenuation factor accounting for absorption and phase mismatch along the propagation path from layer *N* to the surface. It can be expressed as $\Gamma_N = e^{i\Delta k(N-1)t} e^{-\alpha(N-1)t/2}$, where t is the monolayer WS_2 spacing (0.63 nm), α is the absorption coefficient at the SH wavelength (0.086 nm^{-1} at 400 nm for monolayer WS_2), and $\Delta k = \frac{4\pi}{\lambda_0(\omega)}(-n(2\omega) - n(\omega))$ is the wave vector mismatch between the fundamental (ω) and second harmonic (2ω) fields, determined by the refractive indices at ω and 2ω and the excitation wavelength (4.11 at 800 nm and 3.79 at 400 nm for WS_2)[10,11]. The first exponential encodes the phase accumulated due to Δk, while the second exponential describes the reduction of the SH amplitude by absorption as the light travels through the material. According to the SH field superposition picture, the total SH field from an *N* layer stack is then obtained by summing all layer contributions[2,3],

$$\vec{E}^{Total}_{2\omega}(N, \delta) \propto \sum_{M=1}^{N} \Gamma_M \cos\, 3(\phi - \delta_M),$$

and the measurable SHG intensity is proportional to the squared modulus of this total field. In Supplementary Fig. 3 we use this framework, together with the AFM determined thickness, to simulate how the SHG maximum intensity and the main lobe orientation evolve with layer number and interlayer twist, which allows us to convert the measured SHG pattern rotation into an average twist angle per layer.

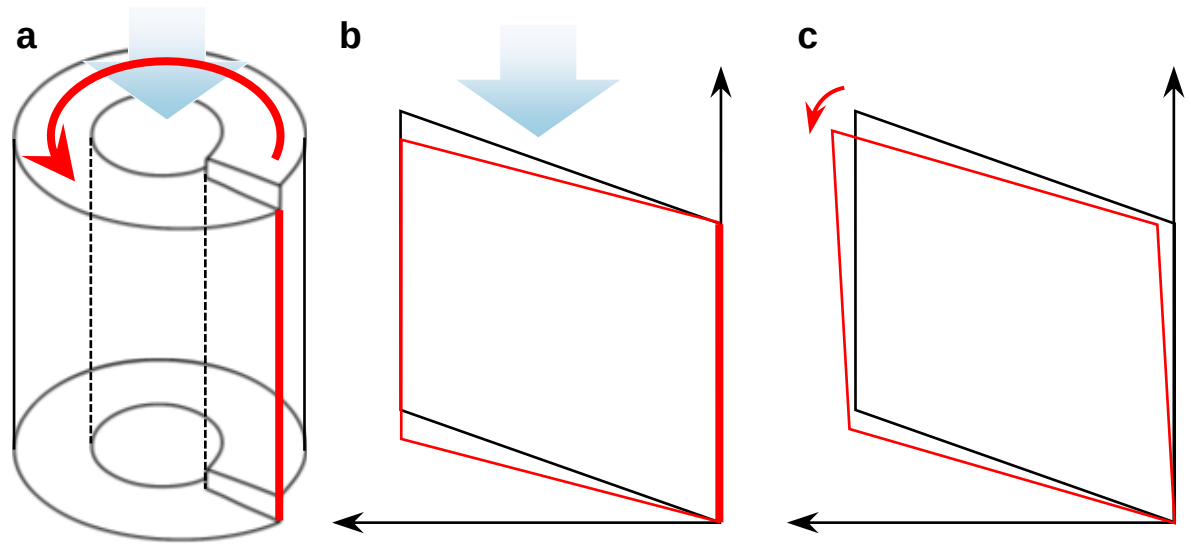

Supplementary Figure 4. Schematic illustration showing how the chirality of a left-handed screw dislocation determines the clockwise twisting tendency of a spiral structure under compression.

a, Cylindrical sketch of a left-handed screw dislocation whose Burgers vector is parallel to the cylinder axis[12], consistent with the spiral geometry discussed in the main text. Under compression (green arrow) the helical planes tend to flatten and the projected Burgers vector along the side surface is shortened, an effect that is equivalent to applying a clockwise shear at the outer rim (red arrow).

b, Conceptual construction used to analyze this deformation: the cylindrical surface at a fixed radius r from the dislocation line is cut along the red line in (a) and unwrapped into a quadrilateral (black frame). Axial compression shortens the vertical direction and produces a sheared quadrilateral (red frame), representing the effective compression of the Burgers vector.

c, Unwrapped view of the same quadrilateral (black frame) after imposing a clockwise twist (red frame). The resulting shape closely matches the compressed configuration in (b), showing that axial compression around a left-handed screw dislocation is mechanically equivalent to a clockwise interlayer twist, analogous to tightening a spring. Upon releasing the axial compression, the lattice constants elastically recover toward their original values (Fig. 3f).

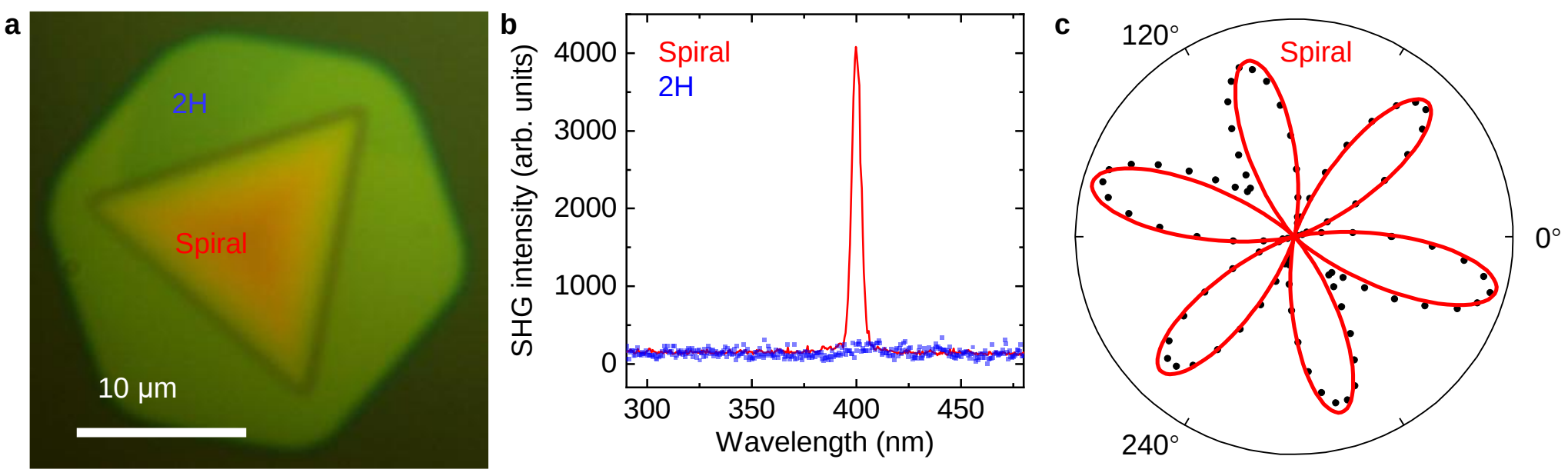

Supplementary Figure 5. SHG characterization of the spiral/2H WS_2 flake used for Raman measurements.

a, Optical image of a multilayer WS_2 flake containing a central spiral domain (red label) surrounded by an adjacent 2H region (blue label). Scale bar, 10 μm.

b, Original SHG spectra acquired from the spiral and adjacent hexagonal 2H regions under identical measurement conditions. The spiral region exhibits a pronounced SHG peak near 400 nm, whereas no detectable SHG peak is observed from the hexagonal region above the background level.

c, Polarization resolved SHG pattern acquired from the spiral region in (a) under parallel polarization detection (black dots, experiment; red curve, fit). The characteristic six lobed response confirms a noncentrosymmetric stacking in the spiral domain, while the surrounding hexagonal region exhibits no detectable SHG signal, consistent with a standard 2H stacking in that area.

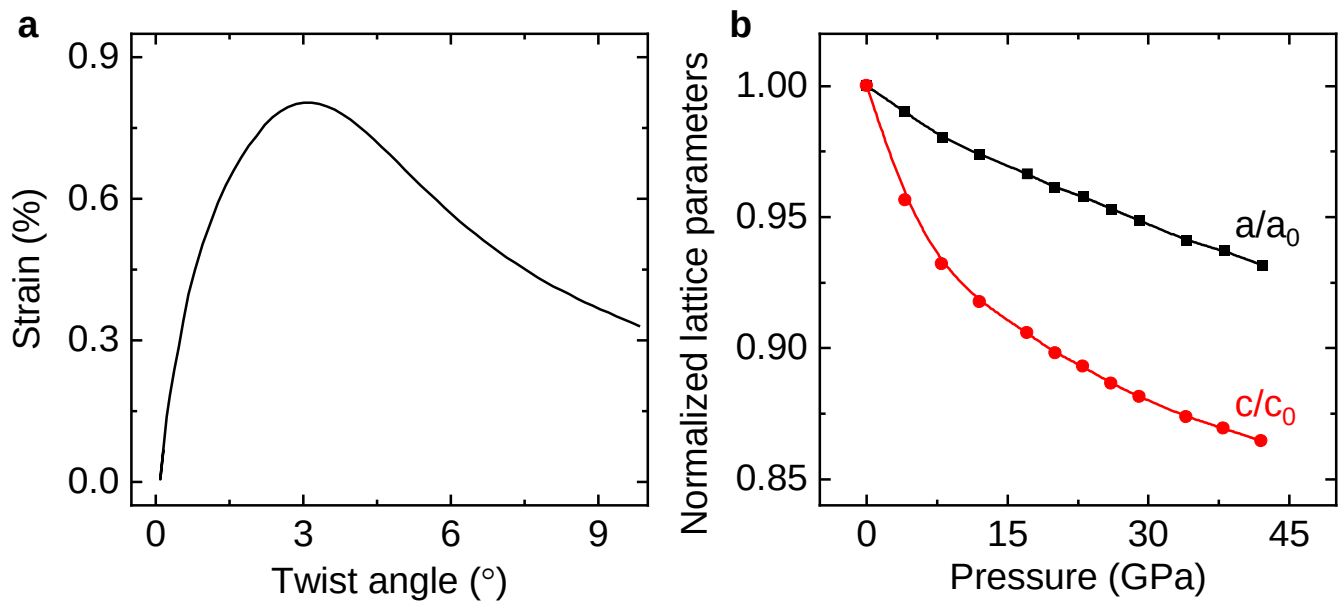

Supplementary Figure 6. Comparison between twist induced reconstruction strain and hydrostatic pressure induced lattice compression in layered TMD crystals.

a, Calculated in plane strain versus interlayer twist angle for reconstructed TMD moiré superlattices, replotted by the authors using data reported in literature[13]. The strain originates mainly from the soliton like domain walls separating nearly commensurate 3R type regions, as illustrated by the atomic configurations in Fig. 6c of the main text. The lattice reconstruction generates an additional in plane tensile strain that reaches a maximum of order 0.8% near a few degrees twist and remains finite down to small angles. At the interlayer twist relevant to our experiment (about 0.2°), the model gives an extra in plane strain of about 0.18%, which is used in the Raman analysis of the E_{2g} mode in the main text.

b, Normalized lattice parameters a/a_0 and c/c_0 of multilayer 2H WS_2 as a function of hydrostatic pressure, measured by synchrotron X ray diffraction and replotted by the authors using data reported in literature[14]. In the low-pressure regime, linear fits to a/a_0 and c/c_0 give in plane and out of plane compressibilities of about 0.22% and 1.06% per GPa, respectively. The much larger out-of-plane slope reflects the higher compressibility of the van der Waals gap. The in-plane value of 0.22% at 1 GPa is comparable to the reconstruction induced in plane strain of about 0.18% in (**a**), supporting the pressure equivalent strain estimate used in the Raman analysis of the main text; the out-of-plane compression of about 1% at 1 GPa is used to evaluate the twist angle associated with a given axial strain (Supplementary Fig. 3c and main text).

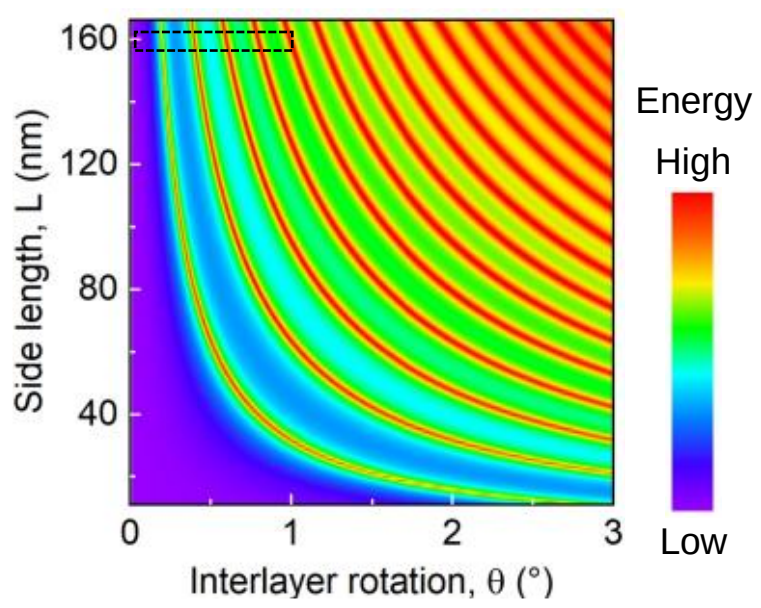

Supplementary Figure 7. Rotational energy landscape of a rigid triangular bilayer WS_2 with initial 3R stacking. Calculated rotational energy landscape $E(\theta,L)$ for a rigid triangular bilayer WS_2 lattice starting from 3R stacking, plotted as a function of interlayer rotation angle θ and triangle side length L. The color map reveals a sequence of energy extrema set by the moiré stacking potential, and shows that the angular positions of the first maximum and minimum become nearly independent of L for sufficiently large triangles. The rotational energy $E(\theta)$ and the corresponding interlayer torque $T(\theta)$, proportional to $\mathrm{d}E/\mathrm{d}\theta$, extracted from the dashed line in the upper-left region are shown in main-text Fig. 6a.

To compute Supplementary Fig. 7, we adopt the analytical moiré potential framework developed by Zhang et al. for the rotational energy of twisted van der Waals bilayers[15,16], using parameters appropriate for WS_2. In this approach, for a hexagonal moiré lattice with initial 3R stacking configuration, the local interfacial energy density is represented by a moiré potential[15-17]:

$$\rho(x,y,\lambda) = \cos\left[\frac{4\pi(x-\frac{\lambda}{\sqrt{3}})}{\sqrt{3}\lambda}\right] + 2\cos\left[\frac{2\pi(x-\frac{\lambda}{\sqrt{3}})}{\sqrt{3}\lambda}\right]\cos\left(\frac{2\pi y}{\lambda}\right)$$

where x and y are in-plane Cartesian coordinates, and $\lambda(\theta) = \frac{R_S}{2\sin(\frac{\theta}{2})}$ is the moiré wavelength, determined solely by the twist angle θ and the lattice constant R_S of the WS_2 layer. This potential reproduces the XM/MX low-energy domains (blue) and MMXX high-energy regions (red) visible in the moiré energy maps of main-text Fig. 6a.

To compare energies for different triangular flake sizes, Zhang et al. introduced a dimensionless rotational energy landscape $E(\theta,r)$ by integrating the local potential over the triangular domain and normalizing it with the flake area[15,16]:

$$E(\theta,r) = \frac{-3[-1+\cos(2\pi r\sqrt{2-2\cos\theta})]}{8\pi^2 r^2(-1+\cos\theta)}$$

where r is a dimensionless size parameter defined as $r = R/a$, with R the circumradius of the equilateral triangle and a is the lattice constant. In our work, Supplementary Fig. 7 is produced by evaluating this expression $E(\theta,L)$ for WS_2 parameters over the relevant range of θ and triangle side length L.

For the specific spiral size (L = 160 nm), we take a cut through the energy map along the horizontal dashed line in Supplementary Fig. 7 to obtain $E(\theta)$, and then differentiate numerically to obtain corresponding interlayer torque $T(\theta)$, as plotted in main-text Fig. 6a. The first positive torque peak near $\theta = 0.20°$ and the following negative minimum near $\theta = 0.28°$ correspond to the energy maximum and minimum highlighted by the moiré energy maps in the insets, providing a microscopic origin for the thermodynamic rotational driving force discussed in the main text.

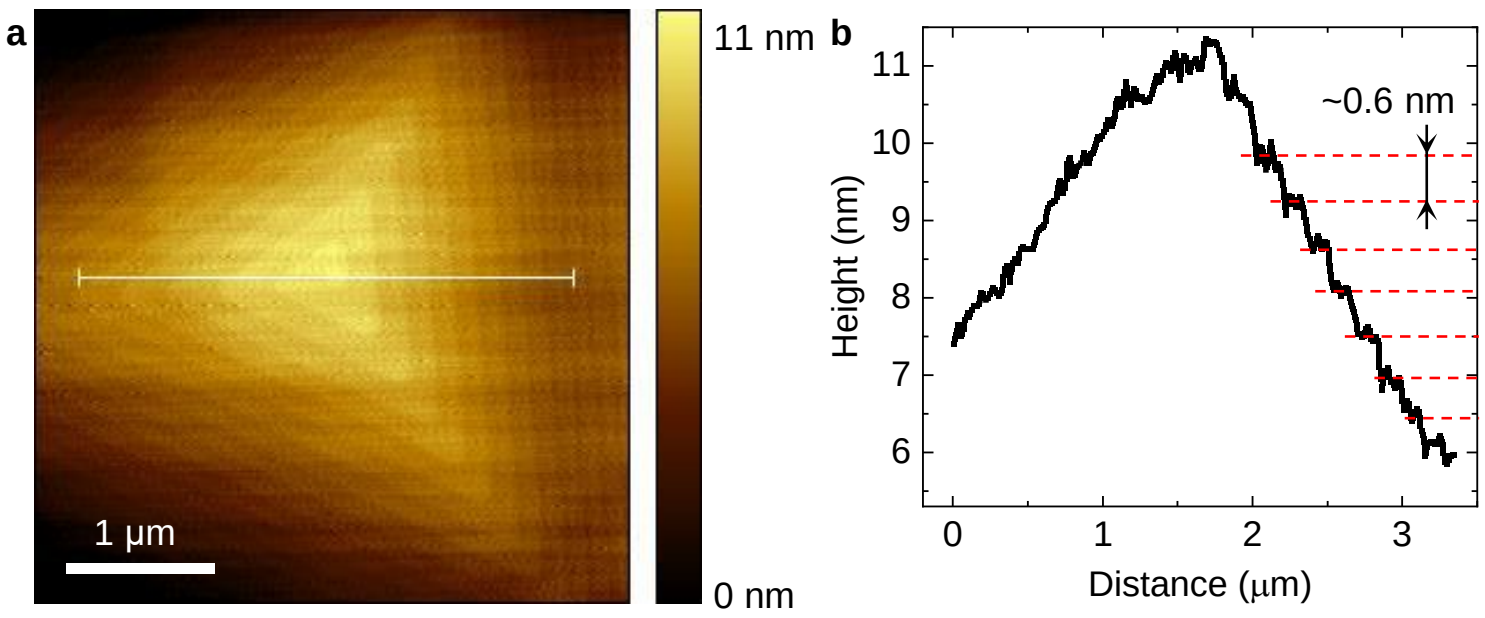

Supplementary Figure 8. AFM topography of the spiral WS_2 crystal.

a, AFM height image of the spiral WS_2 crystal after first-order plane flattening. The color scale indicates the sample height. The white line marks the position used to extract the height profile.

b, Height profile across the spiral core. The step height is about 0.6 nm, consistent with monolayer WS_2 terraces.

The revised map and profile show that the spiral core is the highest region, while the overall height envelope decreases from the core toward the outer terraces, consistent with our previous reports[18-20]. The profile further resolves discrete steps of about 0.6 nm, consistent with monolayer WS_2 terraces. These data provide a clearer visualization of the spiral terraces surrounding the central core and their aligned edges.

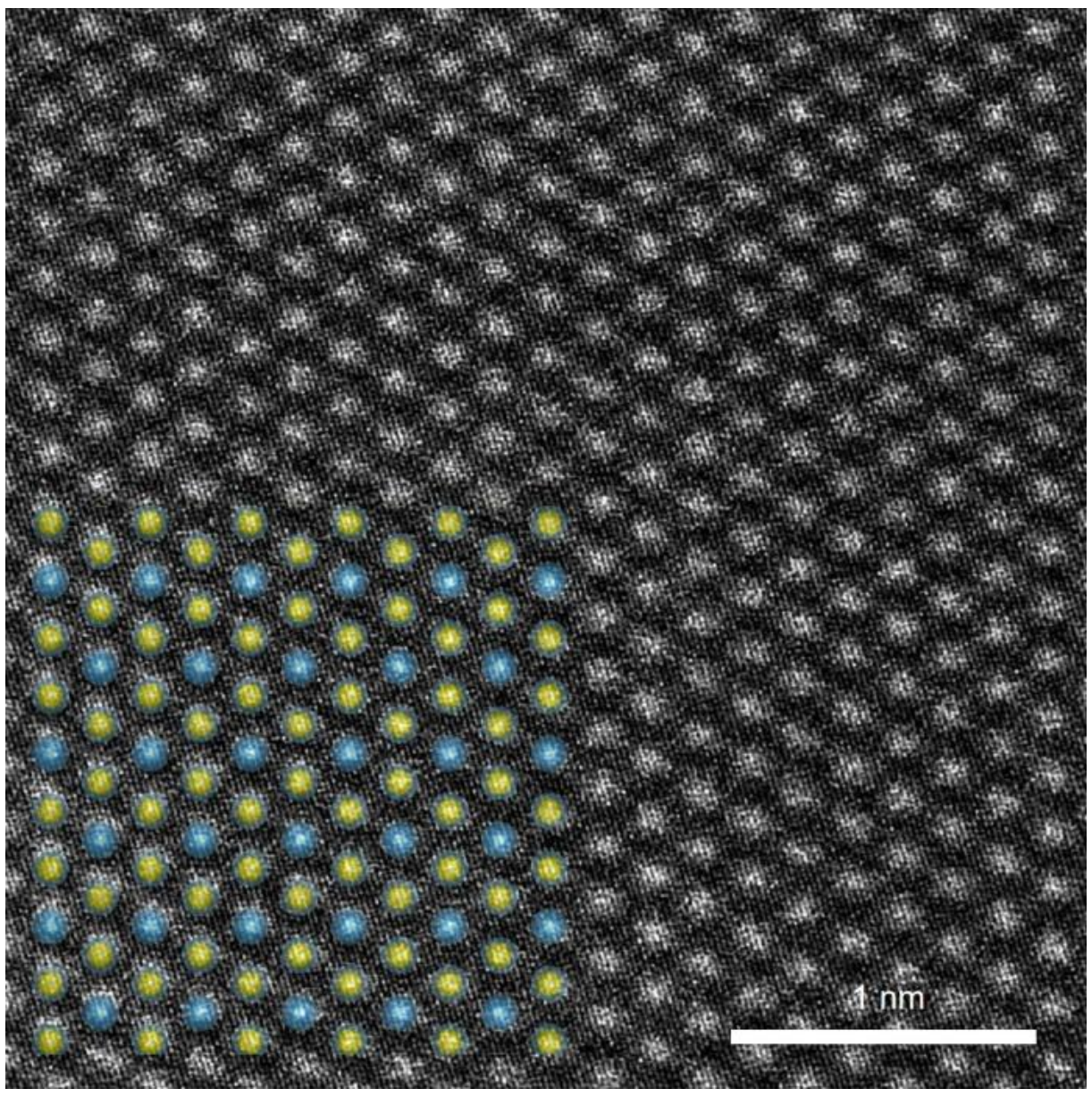

Supplementary Figure 9. Plan-view atomic-resolution HAADF-STEM reference of ideal 3R stacking. The overlaid model identifies representative projected W and S sublattices. Scale bar, 1 nm.

As a qualitative structural reference for ideal 3R registry, we also measured an exfoliated 3R MoS_2 specimen. The 3R reference displays a regular projected atomic pattern with nearly uniform contrast, consistent with the periodic rhombohedral stacking sequence[4,21]. This dataset is used only as a structural reference for ideal 3R registry, rather than for a quantitative comparison of absolute HAADF intensities between MoS_2 and WS_2.

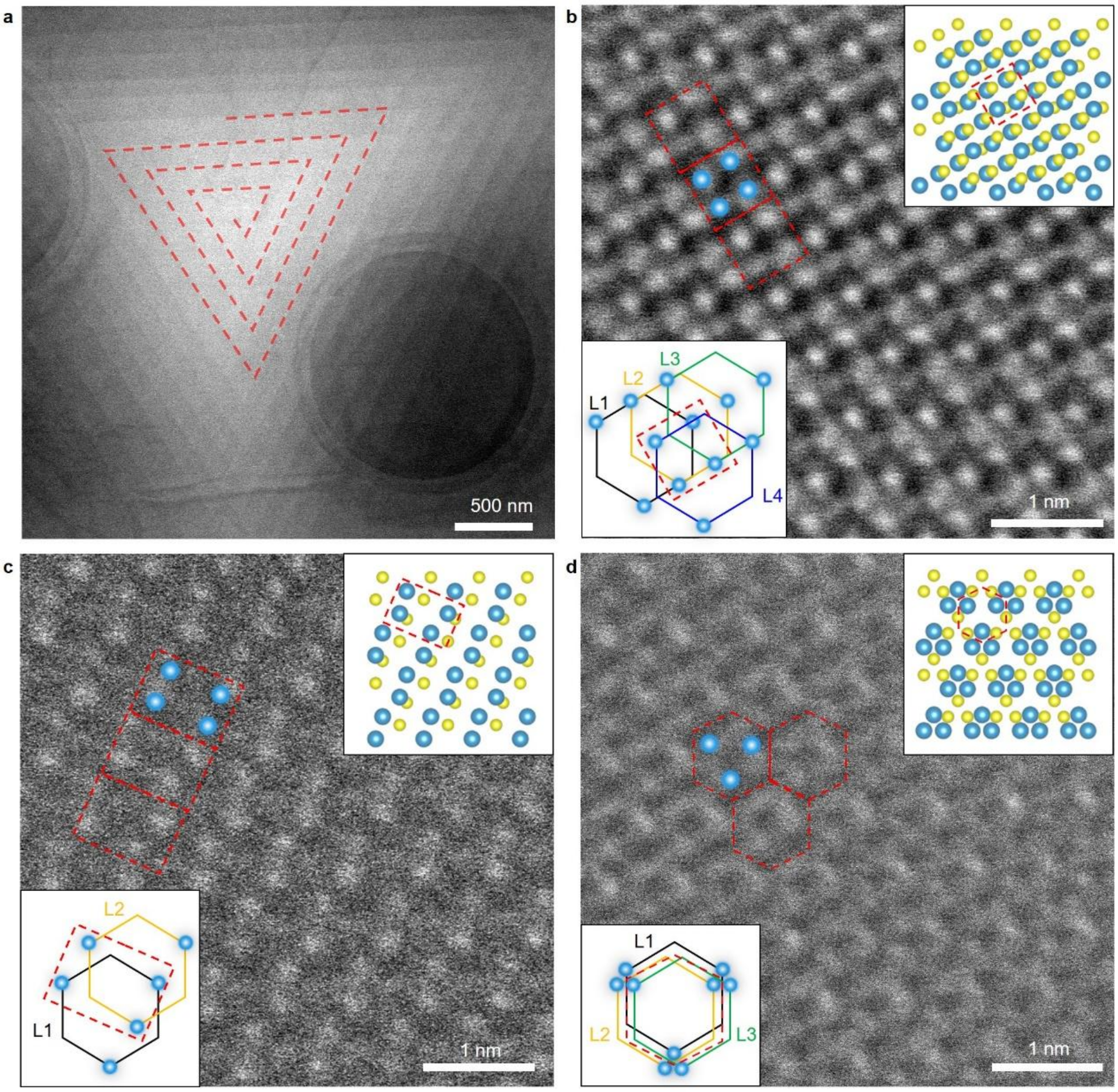

Supplementary Figure 10. Plan-view HAADF-STEM evidence for spatially varying local registry in spiral WS_2.

a, Low-magnification plan-view STEM image of a multilayer spiral WS_2. Red dashed lines trace the spiral terraces.

b-d, Atomic-resolution HAADF-STEM images acquired from different regions of the same spiral crystal, revealing distinct projected atomic motifs. Blue circles mark selected projected W-containing columns. The upper-right insets show projected W/S arrangements, and the lower-left insets show minimal illustrative models comprising four (**b**), two (**c**), and three (**d**) parallel-oriented WS_2 layers, respectively. Red dashed outlines identify corresponding local motifs in the experimental images and models. These models illustrate how nonuniform interlayer translations can qualitatively reproduce the observed projections and are not unique structural reconstructions. Scale bars, 500 nm in a and 1 nm in **b-d**.

Low-magnification TEM image of the overall spiral structure shows that the edge atoms are well aligned (Supplementary Fig. 10a), consistent with the AFM observations (main-text Fig. 2d), indicating a parallel stacking relationship between layers. Unlike regular 3R stacking, the

plan-view HAADF-STEM images of spiral WS_2 reveal spatially varying projected atomic configurations at different locations within the same multilayer spiral crystal (Supplementary Fig. 10b-d). These local motifs are inconsistent with a uniform ideal 3R sequence. Combined with the cross-sectional STEM results (Fig. 2b in the main text), which establish a common in-plane orientation of adjacent layers, the plan-view observations provide independent, spatially resolved evidence for translation-disordered interlayer registry among parallel-oriented WS_2 layers. This result supports the central structural picture of the manuscript: the spiral crystal retains parallel orientation but contains spatially nonuniform lateral sliding rather than a regular ground-state 3R stacking sequence.

To aid interpretation of these representative local motifs, we constructed minimal illustrative projected models comprising four, two, and three parallel WS_2 layers for the upper-right, lower-left, and lower-right HAADF-STEM panels (Supplementary Fig. 10b-d), respectively. In each model, L_1, outlined in black, serves as the reference layer, whereas the colored hexagons denote later layers laterally translated relative to L_1. The lower-left inset in each panel shows the relative positions of the layer-specific W sublattices, and the upper-right inset shows the corresponding projected W/S arrangement. The red dashed outlines identify equivalent local motifs in the experimental HAADF-STEM images and in the projected structural models, while blue circles mark the projected W-containing columns used for comparison. These nonuniform in-plane translations qualitatively reproduce the distinct projected motifs observed at different locations. We emphasize that this model is intended as a visual illustration rather than a unique structural reconstruction. Different translation combinations or local strain induced by screw dislocation can in principle generate related projected motifs.

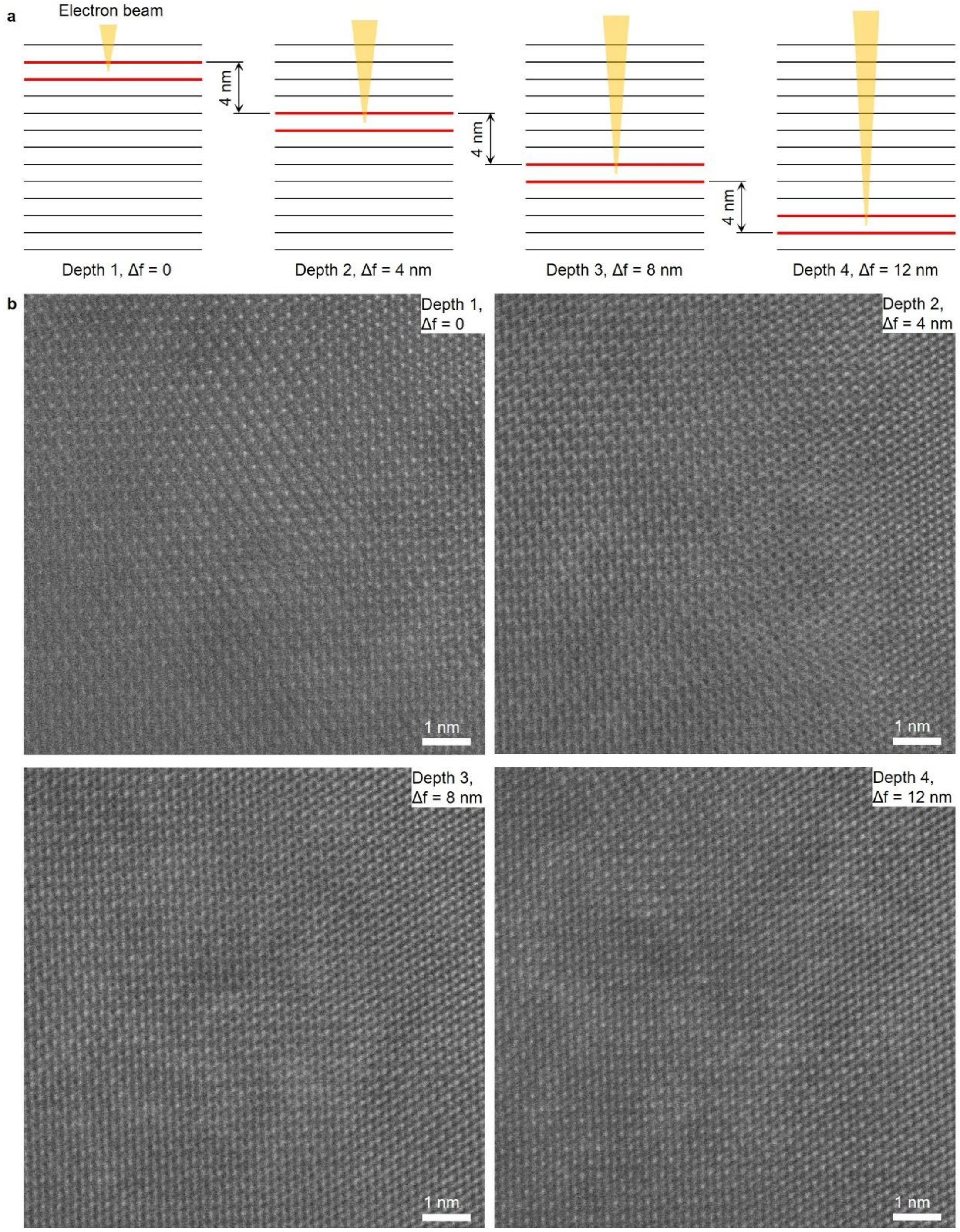

Supplementary Figure 11. Focal-series HAADF-STEM of multilayer spiral WS_2.

a, Schematic of the nominal focal conditions used for the focal series. Red lines indicate the nominal depth ranges preferentially weighted at each defocus setting.

b, Atomic-resolution HAADF-STEM images collected from the same region at Δf = 0, 4, 8, and 12 nm. The focus-dependent changes in projected atomic contrast are consistent with depth-dependent interlayer registry and translational disorder. Scale bars, 1 nm.

We collected an atomic-resolution HAADF-STEM focal series from the same multilayer spiral WS_2 region at different probe defocus values (Supplementary Fig. 11). Under the present imaging conditions of 300 kV and a 25 mrad convergence semi-angle, the axial response of the STEM probe extends over several nanometres, substantially exceeding the interlayer spacing of WS_2. We therefore do not interpret the focal series as direct imaging of individual layers. Rather, changing the probe focus modifies the relative contribution of distinct stacking configurations along the beam direction to the projected HAADF contrast. The resulting focus-dependent change in the apparent atomic arrangement provides additional real-space evidence consistent with depth-dependent interlayer registry and translational disorder.

Together, these plan-view (Supplementary Fig. 10-11) and cross-sectional STEM (Fig. 2b) measurements support a parallel-oriented, translation-disordered, non-3R stacking structure in spiral WS_2.

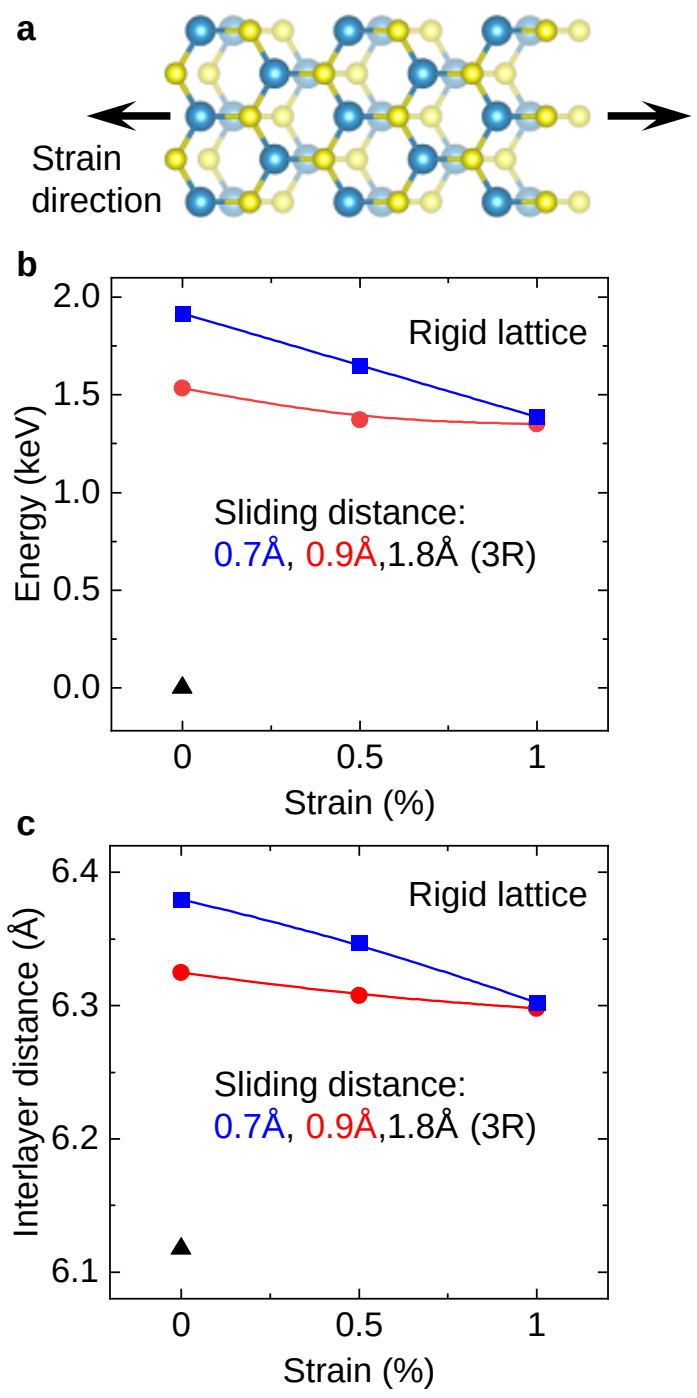

Supplementary Figure 12. Strain-dependent energy and interlayer spacing of rigid slipped bilayer WS_2 configurations.

a, Schematic of the applied uniaxial in-plane strain along the direction indicated.

b-c, Calculated total energy and corresponding equilibrium interlayer distances as a function of strain for bilayer WS_2 with fixed lateral sliding distances of 0.7 Å (blue) and 0.9 Å (red). The black triangle denotes the ideal 3R reference configuration, corresponding to a 1.8 Å lateral translation.

The calculations show that the slipped configurations have a less favourable registry-dependent interlayer interaction energy and a larger equilibrium spacing than ideal 3R stacking. Importantly, the uniaxial strain field lowers the total energy and interlayer distance of the slipped configurations relative to their unstrained counterparts (Supplementary Fig. 12). This allows screw-dislocation-induced strain to stabilize non-3R registries as energetically accessible metastable configurations despite their higher energy than ideal 3R stacking.

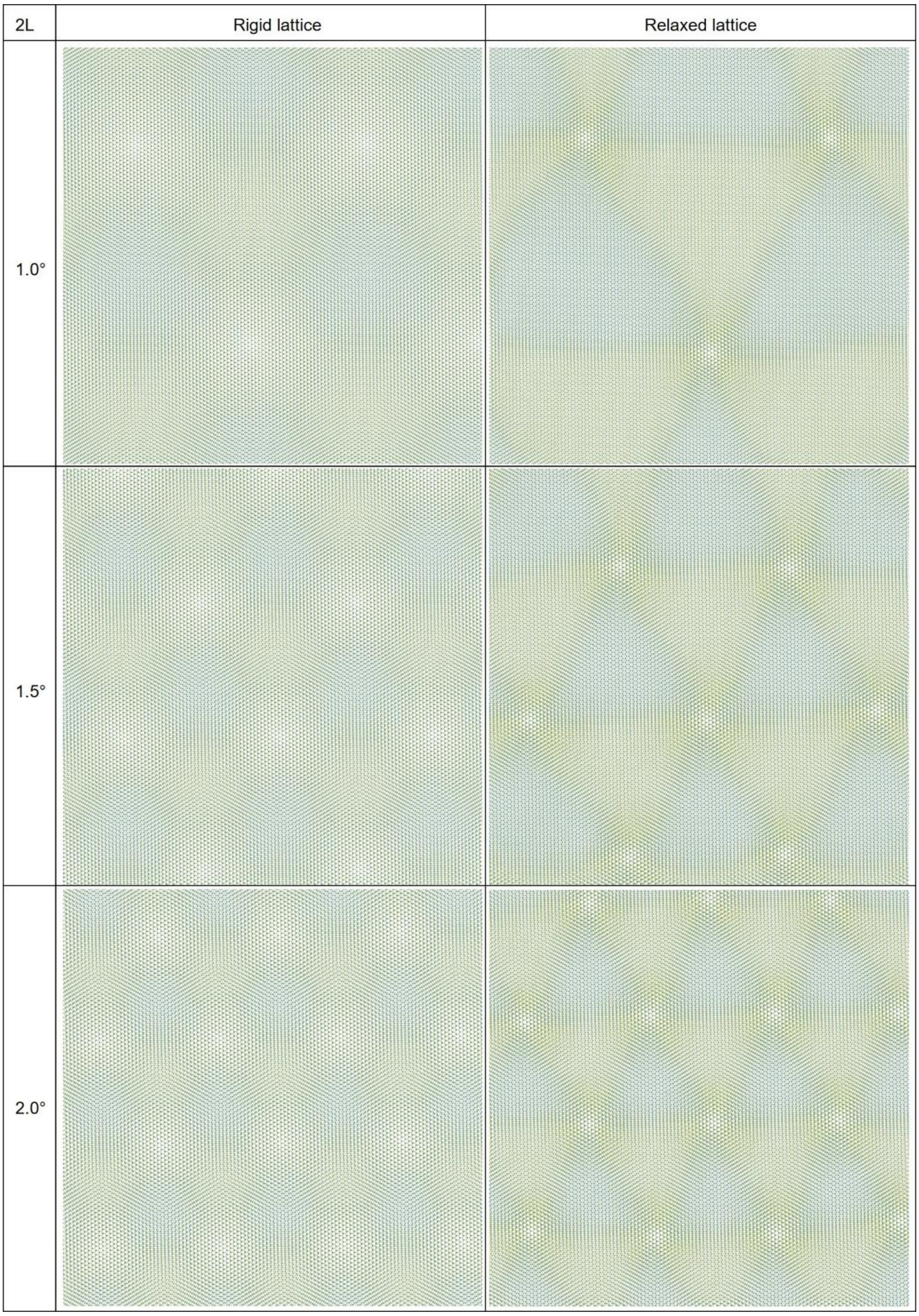

Supplementary Figure 13. Moiré supercells of twisted bilayer WS_2 at small twist angles (θ = 1.0°, 1.5°, and 2.0°). The moiré supercells contain 110,286 atoms with lateral dimensions of approximately 490 Å × 490 Å. The left and right columns show the rigid-lattice and relaxed-lattice atomistic configurations, respectively.

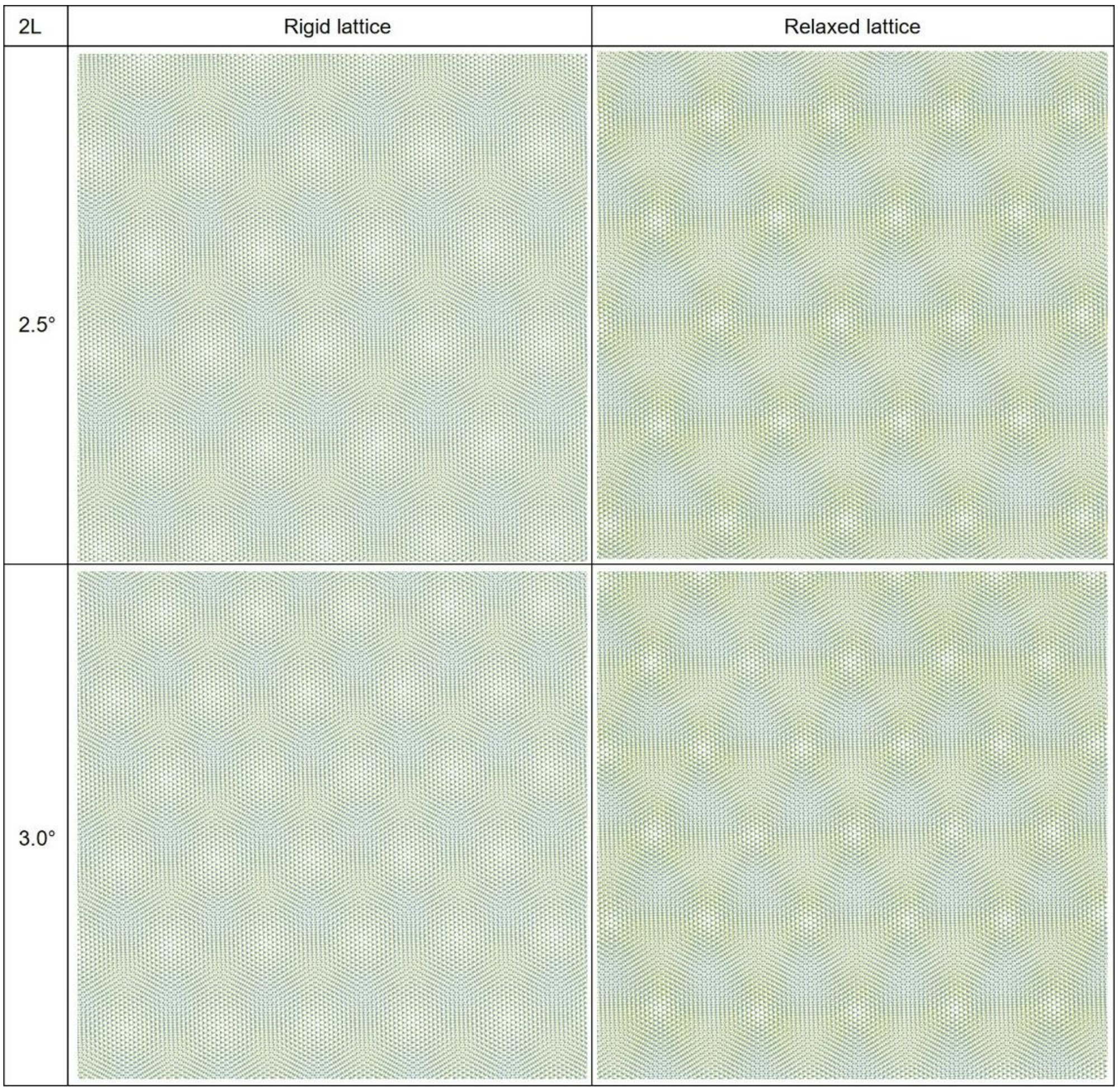

Supplementary Figure 14. Moiré supercells of twisted bilayer WS_2 at intermediate twist angles (θ = 2.5° and 3.0°). The moiré supercells contain 110,286 atoms with lateral dimensions of approximately 490 Å × 490 Å. The left and right columns show the rigid-lattice and relaxed-lattice atomistic configurations, respectively.

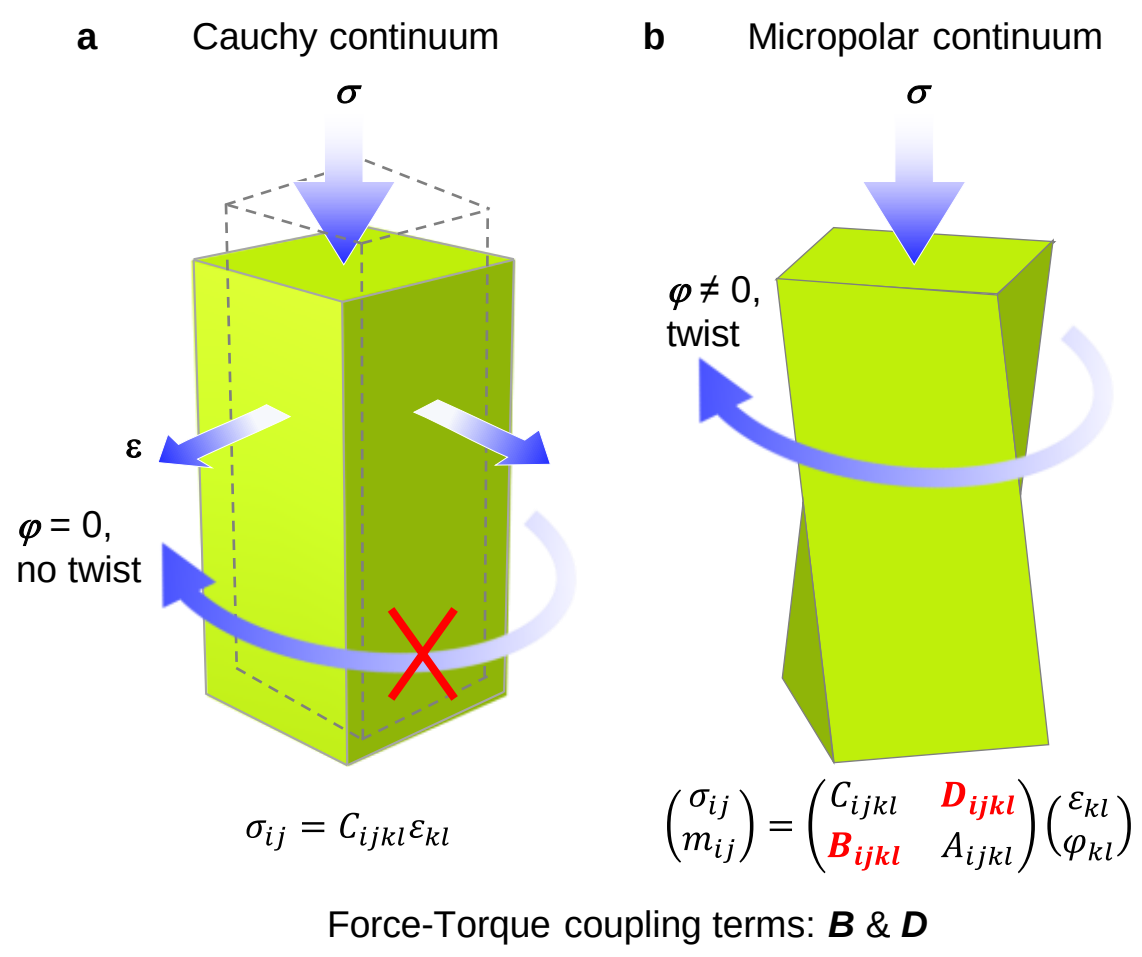

Supplementary Figure 15. Schematic comparison between Cauchy and micropolar descriptions of compression-twist coupling.

a, under uniaxial stress (σ), a homogeneous Cauchy continuum develops conventional strain components (ε) but has no independent microrotation, $\varphi = 0$.

b, a micropolar continuum additionally admits an internal microrotation φ. The cross-coupling tensors B_{ijkl} and D_{ijkl} enable coupling between axial deformation and rotational response[6].

In a homogeneous Cauchy linear elasticity, the force-stress tensor σ_{ij} is related to the symmetric strain tensor ε_{kl} through $\sigma_{ij} = C_{ijkl}\,\varepsilon_{kl}$. The rank-four elasticity tensor C_{ijkl} is invariant under space inversion and therefore does not provide an intrinsic axial force-to-torque constitutive coupling[6]. Accordingly, a homogeneous Cauchy effective medium cannot exhibit a macroscopic twist response under pure axial loading (Supplementary Fig. 15a).

A micropolar continuum extends this description by introducing an independent microrotation field θ_k and its associated curvature tensor $\varphi_{kl} = \partial\theta_k/\partial x_l$, together with the couple-stress tensor m_{ij} [8]. The generalized constitutive relation can be written as $\begin{pmatrix}\sigma_{ij}\\ m_{ij}\end{pmatrix} = \begin{pmatrix}C_{ijkl} & D_{ijkl}\\ B_{ijkl} & A_{ijkl}\end{pmatrix}\begin{pmatrix}\varepsilon_{kl}\\ \varphi_{kl}\end{pmatrix}$ [6-8]. Here, C_{ijkl} describe the conventional force-strain, whereas A_{ijkl} describes the couple-stress response to microrotation gradients. The off-diagonal tensors B_{ijkl} and D_{ijkl} describe force-rotation coupling: strain-like deformation can generate couple stress through B_{ijkl}, whereas rotation gradients can contribute to force stress through D_{ijkl}. Such cross-coupling terms are symmetry-allowed in micropolar media and provide the continuum-mechanics basis for compression-twist conversion (Supplementary Fig. 15b).

For spiral WS_2, we use this micropolar description as a conceptual effective-medium analogy rather than as a quantitative constitutive fit. The translation-disordered interfaces provide an internal structural coordinate, namely the local interlayer registry and rotation, that is absent from a homogeneous Cauchy description. Under compression, the reduction in interlayer spacing modifies the moiré-modulated stacking-energy landscape and can generate an interlayer rotational torque through atomic reconstruction. In this sense, the pressure-sensitive moiré interface acts as an atomic-scale analogue of a force-rotation-coupled micropolar layer.

This framework connects the central observation of this work, namely pressure-driven interlayer twist in spiral WS_2, with the broader concept of compression-twist coupling in micropolar continuum mechanical systems.

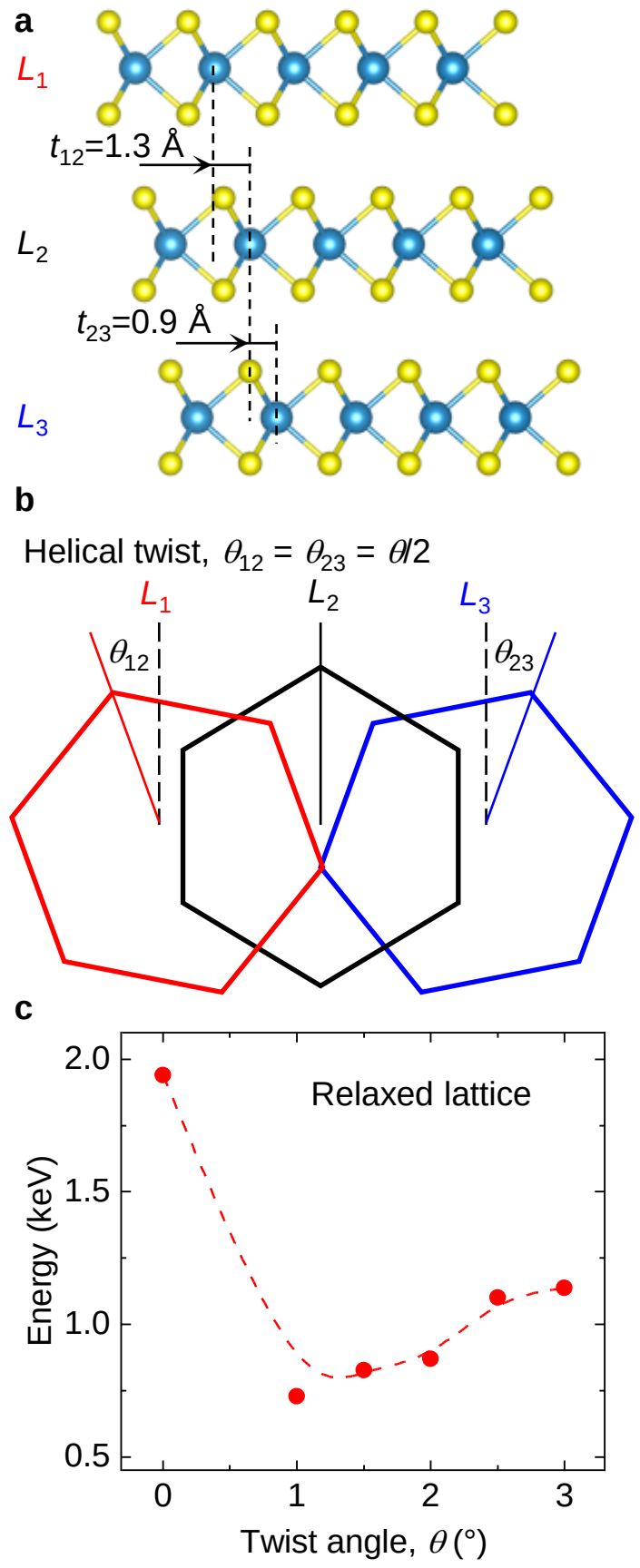

Supplementary Figure 16. STEM-constrained helical trilayer model of spiral WS_2.

a, Side-view structural model of three parallel-oriented WS_2 layers. The armchair-projected translations between L_1/L_2 and L_2/L_3 are set to $t_{12} = 1.3$ Å and $t_{23} = 0.9$ Å, respectively, based on representative shifts resolved by cross-sectional STEM. Blue and yellow spheres denote W and S atoms, respectively.

b, Definition of the helical twist configuration. L_1, L_2, and L_3 are rotated by $-\theta$, 0, and $+\theta$, respectively, giving $\theta_{12} = \theta_{23} = \theta/2$.

c, Total energy of the STEM-constrained trilayer model as a function of twist angle θ.

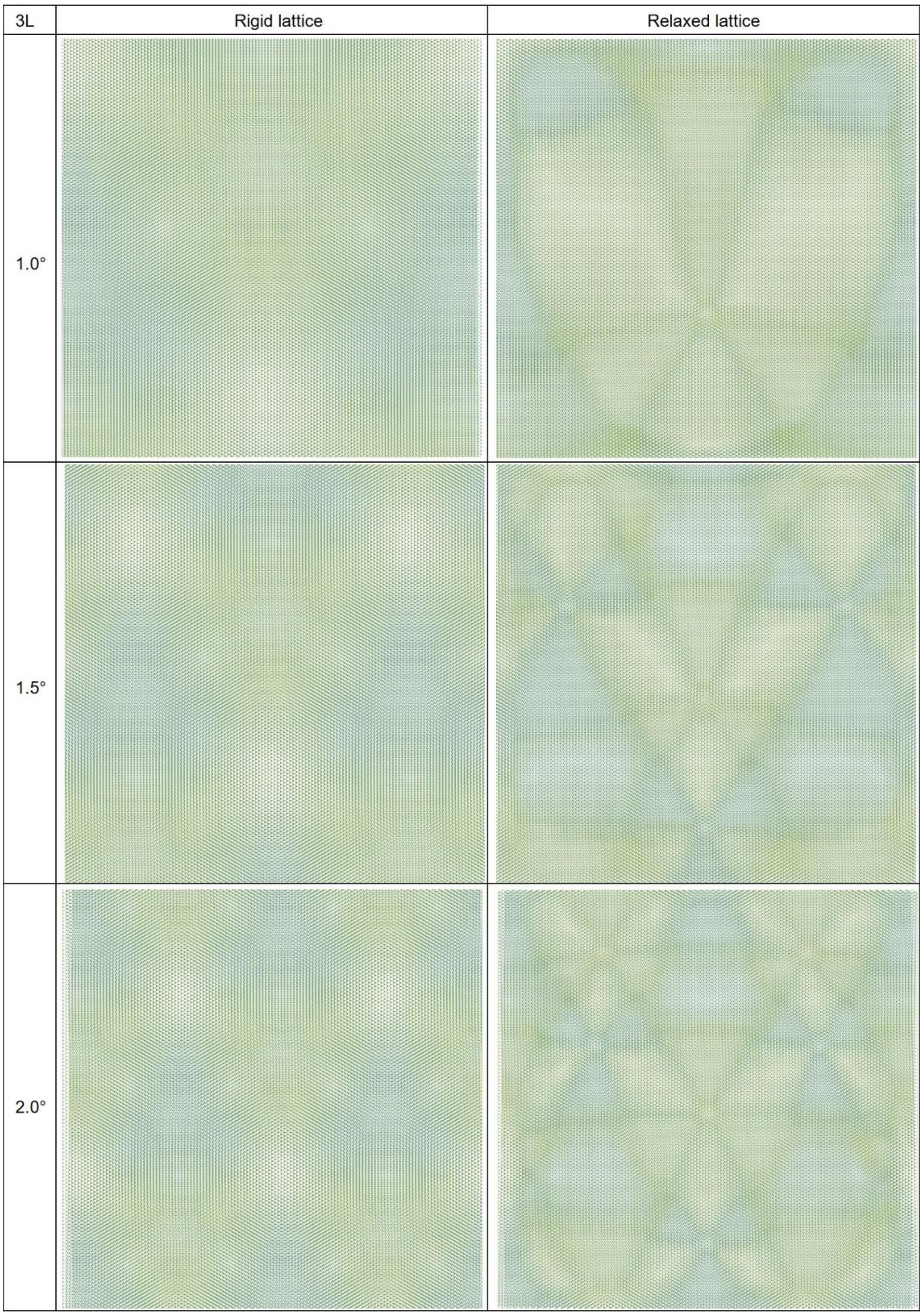

Supplementary Figure 17. Moiré supercells of twisted trilayer WS_2 at small twist angles (θ = 1.0°, 1.5°, and 2.0°). The moiré supercells contain 160,065 atoms with lateral dimensions of approximately 490 Å × 490 Å. The left and right columns show the rigid-lattice and relaxed-lattice atomistic configurations, respectively.

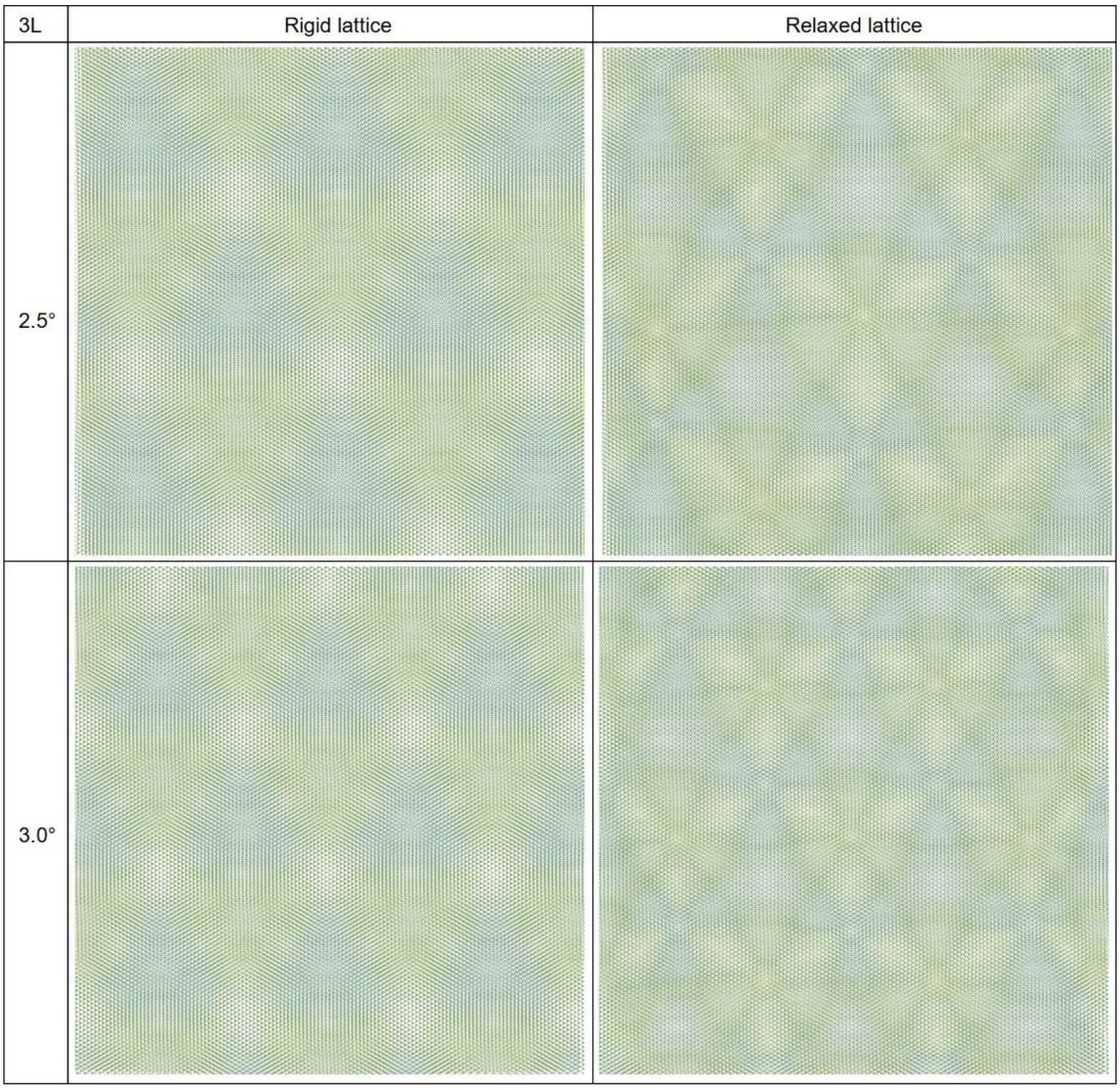

Supplementary Figure 18. Moiré supercells of twisted trilayer WS_2 at intermediate twist angles (θ = 2.5° and 3.0°). The moiré supercells contain 160,065 atoms with lateral dimensions of approximately 490 Å × 490 Å. The left and right columns show the rigid-lattice and relaxed-lattice atomistic configurations, respectively.

We constructed a trilayer WS_2 model containing 160,065 atoms in a supercell of approximately 490 Å × 490 Å (Supplementary Fig. 17-18). The successive interlayer translations were set to t_{12} = 1.3 Å and t_{23} = 0.9 Å (Supplementary Fig. 16a), corresponding to representative projected shifts measured by STEM. A helical twist configuration was imposed with $\theta_{12} = \theta_{23} = \theta/2$, corresponding to layer orientations of $-\theta/2$, 0, and $+\theta/2$ for L_1, L_2, and L_3, respectively (Supplementary Fig. 16b). This trilayer model contains two coupled moiré interfaces and allows the middle layer to respond simultaneously to the registry-dependent interactions from both neighbouring layers. The rigid and relaxed trilayer configurations reveal atomic reconstruction over the range θ = 1.0° to 3.0° (Supplementary Fig. 17-18). More importantly, the relaxed trilayer energy decreases substantially from θ = 0° and reaches a minimum near small non-zero twist angle, followed by an increase at larger twist angles (Supplementary Fig. 16c). Thus, finite-angle stabilization persists in a minimal multilayer model containing two coupled interfaces and is consistent with the energy-lowering trend obtained for the STEM-constrained slipped bilayers.

We emphasize that the trilayer model is not intended to reproduce the exact registry distribution or layer-resolved twist profile of the full multilayer spiral crystal. Rather, it establishes that the reconstruction-mediated rotational energy landscape remains operative beyond a single bilayer interface, supporting the extension of the proposed compression-twist mechanism to the multilayer spiral structure.

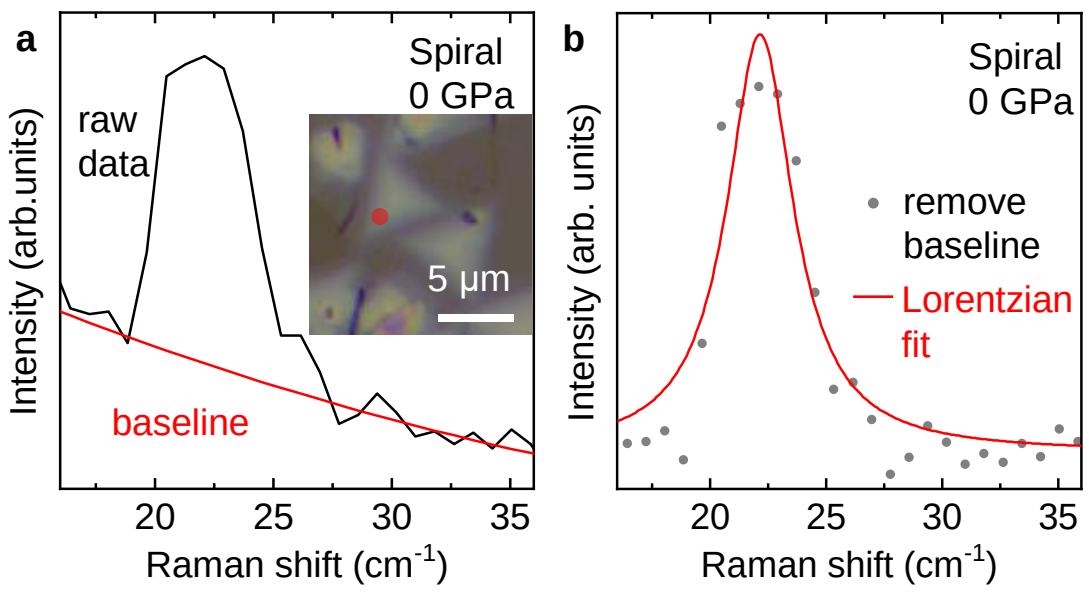

Supplementary Figure 19. Ultralow-frequency Raman signatures of pressure-dependent interlayer shear coupling in spiral WS_2.

a-b, Raw ambient-pressure ultralow-frequency Raman spectrum of spiral WS_2 measured at the edge region marked by the red point in the inset, the fitted spectral baseline, and the baseline-subtracted spectrum fitted with a Lorentzian line shape. The spiral WS_2 was loaded on the diamond culet of the DAC.

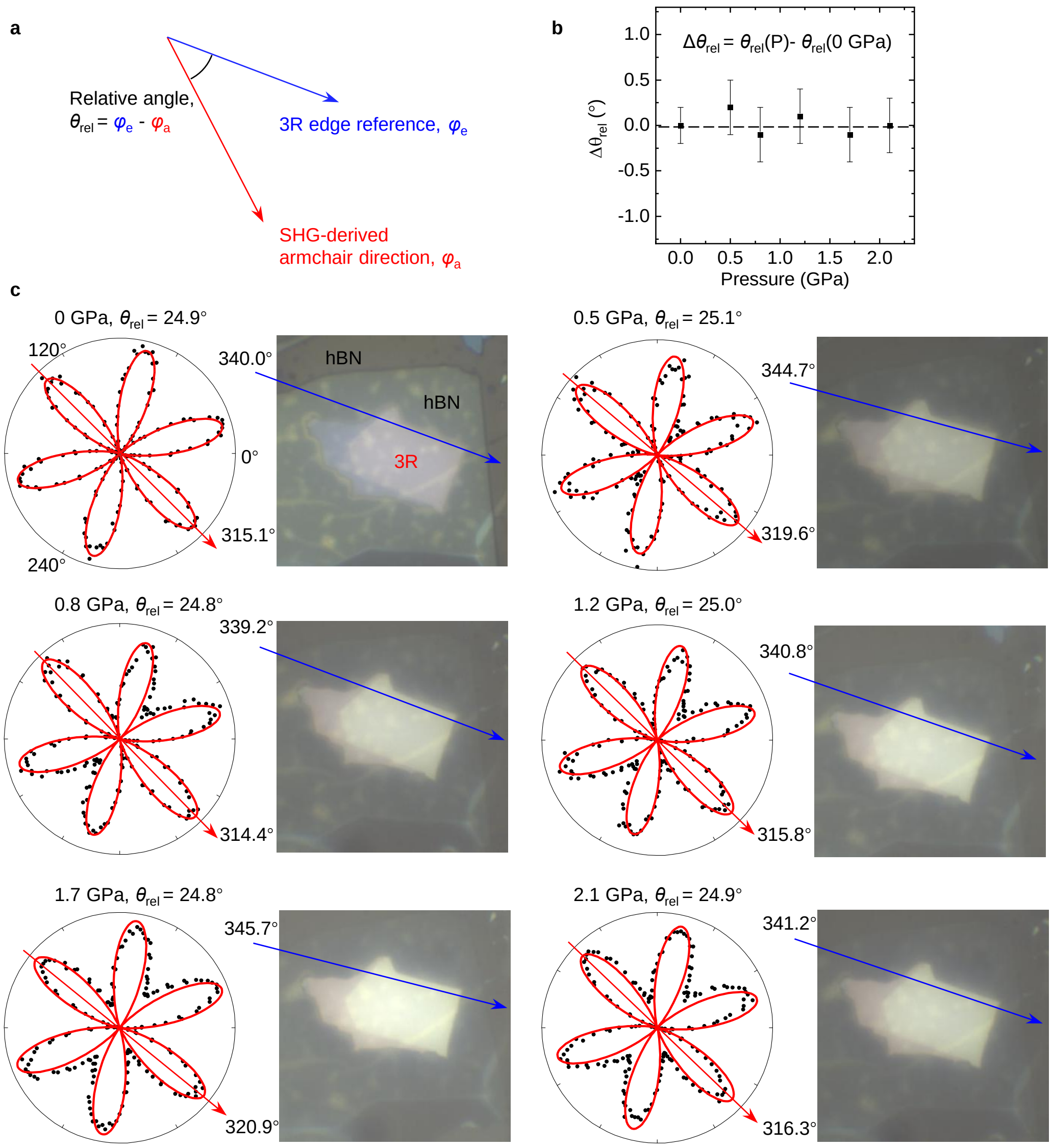

Supplementary Figure 20. Pressure-dependent polarization-resolved SHG response of a regular 3R-stacked TMD reference.

a, Definition of the relative angle, $\theta_{rel} = \varphi_e - \varphi_a$, where φ_e is the direction of a straight crystalline edge and φ_a is the armchair direction extracted from the fitted SHG pattern.

b, Pressure dependence of $\Delta\theta_{rel} = \theta_{rel}(P) - \theta_{rel}(0\ \text{GPa})$ for mechanically exfoliated 3R MoS_2 measured under the same DAC and optical conditions as spiral WS_2. Error bars represent the fitting uncertainties of the SHG pattern rotation. No systematic pressure-dependent rotation is observed within experimental uncertainty.

c, Polarization-resolved SHG patterns and corresponding optical images of the 3R MoS_2 crystal measured at 0, 0.5, 0.8, 1.2, 1.7, and 2.1 GPa, respectively. Black symbols denote measured SHG intensities and red curves denote the corresponding fits. Blue arrows indicate the edge reference direction, whereas red arrows indicate the SHG-derived armchair direction.

We have performed pressure-dependent polarization-resolved SHG measurements on a mechanically exfoliated 3R MoS_2 crystal as a control for a regular parallel-stacked TMD. The measurements were conducted under the same DAC and optical conditions used for spiral WS_2, including hBN encapsulation, silicone-oil pressure medium, diamond anvils with 800 μm culets, and the same polarization-resolved SHG configuration.

The 3R reference exhibits a well-defined sixfold SHG pattern throughout the pressure range from ambient pressure to 2.1 GPa (Supplementary Fig. 20). To quantify any pressure-induced reorientation, we defined the relative angle as $\theta_{rel} = \varphi_e - \varphi_a$, where φ_e is the direction of a straight crystalline edge and φ_a is the armchair direction extracted from the fitted SHG pattern. We then evaluated $\Delta\theta_{rel} = \theta_{rel}(P) - \theta_{rel}(0\ GPa)$. Within experimental uncertainty, $\Delta\theta_{rel}$ remains centred around zero over the entire pressure range. Thus, regular 3R stacking does not show a systematic pressure-dependent rotation of the SHG pattern relative to the crystallographic edge reference under the present experimental conditions.

This control has two implications. First, it confirms that the combined effects of compressed diamond, silicone oil, hBN encapsulation, and the polarization-resolved optical path do not produce an artificial rotation of the SHG pattern. Second, it shows that hydrostatic compression alone does not induce an observable reorientation in a regular 3R-stacked TMD. The pressure-dependent SHG rotation observed in spiral WS_2 is therefore attributed to its translation-disordered, metastable interlayer registry rather than to a generic response of parallel-stacked TMDs under pressure.

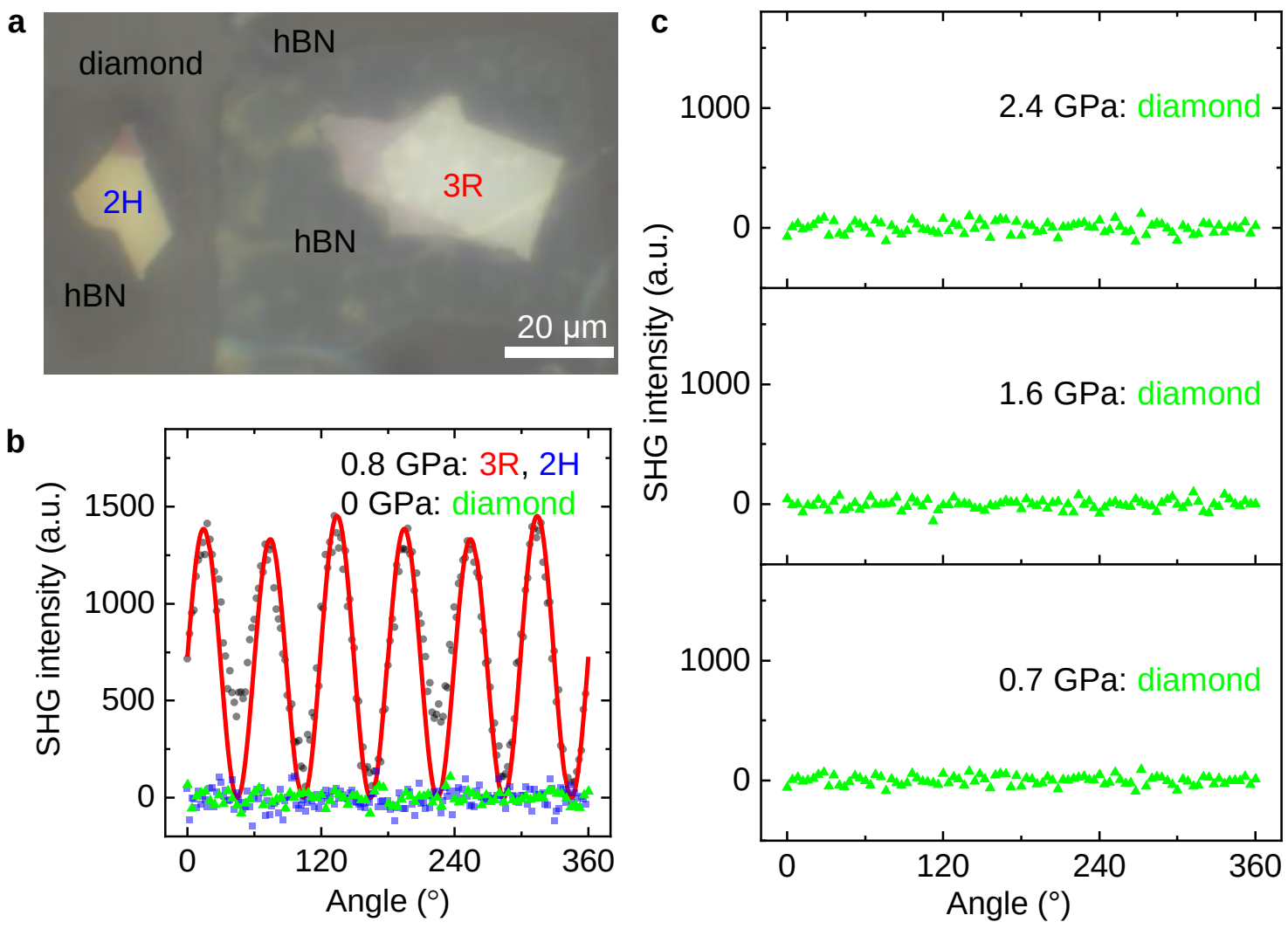

Supplementary Figure 21. High-pressure SHG control of bulk 2H and 3R MoS_2 with diamond-background measurements.

a, Optical image of the 2H and 3R MoS_2 regions encapsulated by hBN and loaded in the diamond-anvil cell at 0.8 GPa.

b, Polarization-resolved SHG intensities of the 3R and 2H regions measured under the same DAC and optical conditions as those used for spiral WS_2, including 800 nm excitation, hBN encapsulation, silicone-oil pressure medium, and diamond anvils. Grey circles and red curve denote the measured SHG intensity and fit for the 3R region, respectively. Blue squares denote the signal from the 2H region. Green triangles denote the representative diamond-background signal measured at 0 GPa and plotted on the same intensity scale. The 3R reference exhibits a pronounced sixfold SHG pattern, whereas the 2H region and diamond background show no detectable SHG signal above the background.

c, Pressure-dependent diamond-background SHG signals measured at 0.7, 1.6, and 2.4 GPa using the same DAC optical configuration. The diamond-background data in **b** and **c** were acquired from a separate diamond-control loading batch, whereas the 3R and bulk 2H data in **b** were acquired from the same hBN-encapsulated sample loading batch.

To assess the combined optical effects of the diamond anvils, pressure medium, hBN encapsulation, and polarization-resolved SHG measurement geometry, we performed control measurements on hBN-encapsulated 2H and 3R MoS_2 flakes (prepared by mechanical exfoliation) under the same DAC and optical conditions as those used for the spiral sample (Supplementary Fig. 21a). At 0.8 GPa, the 3R reference exhibits a pronounced sixfold SHG pattern, whereas the adjacent 2H region shows no detectable SHG signal above the background level (Supplementary Fig. 21b). To directly assess the diamond contribution, we further measured the SHG response from the diamond background at different pressures. The 0 GPa diamond-background data are plotted together with the 3R and bulk 2H data in Supplementary Fig. 21b on the same intensity scale. The diamond signal also remains close to the noise level

at 0.7, 1.6, and 2.4 GPa and shows no resolvable angular modulation (Supplementary Fig. 21c). These comparisons confirm that the hBN encapsulation, compressed silicone oil, and diamond optical path do not generate an artificial sixfold SHG response under the present measurement conditions.

More importantly, we measured the pressure-dependent polarization-resolved SHG response of the 3R reference from ambient pressure to 2.1 GPa (Supplementary Fig. 20). Within experimental uncertainty, the combined high-pressure optical environment does not induce a systematic pressure-dependent rotation of the SHG pattern relative to the crystallographic edge reference.

These controls directly rule out, within the experimental uncertainty, the relevant artifact of a systematic rotation of the polarization-dependent SHG pattern caused by compressed diamond, silicone oil, or hBN encapsulation. The pressure-dependent relative rotation observed in spiral WS_2 is therefore attributed to an intrinsic structural response of the spiral stack rather than to the DAC optical environment.

Supplementary Note 1. Atomic-level simulation details.

To understand the experimental observations, we performed atomic level simulations on layered WS_2 to determine how the total energy depends on interlayer sliding, interlayer rotation, and atomic reorganization. Generating realistic moiré patterns in layered WS_2 requires supercells containing several thousand atoms, which renders first principles calculations very challenging, if not impractical. We therefore adopted empirical potentials, using Stillinger–Weber and Lennard-Jones (LJ) parameters with slight modifications[22,23]. These parameters were implemented in the QuantumATK package for structural relaxation and total energy calculations.

We constructed bilayer and trilayer WS_2 models. The bilayer model contains about 110,286 atoms in a supercell of approximately 490 Å × 490 Å × 35 Å. The simulations start from a 3R-stacked structure, which is first fully relaxed to obtain the lowest-energy configuration. We then slide one WS_2 layer by 0.7 Å, 0.9 Å, 1.3 Å, 1.8 Å, and 2.1 Å along the direction from MMXX stacking toward 3R stacking (1.8 Å), respectively, and record the corresponding increase in total energy. For each sliding configuration, the top layer is further rotated by 1°, 1.5°, 2°, 2.5°, and 3°, followed by full relaxation to examine atomic reorganization, the evolution of the moiré pattern, and the change in total energy.

The trilayer model contains about 160,065 atoms in a supercell of approximately 490 Å × 490 Å × 35 Å. We then slide the middle layer by 0.9 Å and the top layer by 1.3 Å along the same in-plane direction relative to the underlying layer, respectively, and record the corresponding increase in total energy. Next, the top layer is further rotated by $+\theta/2$, while the underlying layer is rotated by $-\theta/2$, followed by full relaxation to examine atomic reorganization, the evolution of the moiré pattern, and the change in total energy. The negative sign indicates that the twist direction is opposite to that of the topmost layer.

Supplementary References

1 Mennel, L. *et al.* Optical imaging of strain in two-dimensional crystals. *Nature Communications* **9**, 516, doi:10.1038/s41467-018-02830-y (2018).

2 Ci, P. *et al.* Breaking Rotational Symmetry in Supertwisted WS2 Spirals via Moiré Magnification of Intrinsic Heterostrain. *Nano Letters* **22**, 9027-9035, doi:10.1021/acs.nanolett.2c03347 (2022).

3 Zhao, M. *et al.* Atomically phase-matched second-harmonic generation in a 2D crystal. *Light: Science & Applications* **5**, e16131-e16131, doi:10.1038/lsa.2016.131 (2016).

4 Shi, J. *et al.* 3R MoS2 with Broken Inversion Symmetry: A Promising Ultrathin Nonlinear Optical Device. *Advanced Materials* **29**, 1701486, doi:https://doi.org/10.1002/adma.201701486 (2017).

5 Coulais, C. As the extension, so the twist. *Science* **358**, 994-995, doi:doi:10.1126/science.aaq0818 (2017).

6 Frenzel, T., Kadic, M. & Wegener, M. Three-dimensional mechanical metamaterials with a twist. *Science* **358**, 1072-1074, doi:doi:10.1126/science.aao4640 (2017).

7 Montazeri, A. & Mahnama, M. Twisting chiral mechanical metamaterials: A review. *Materials Today Communications* **44**, 112070, doi:https://doi.org/10.1016/j.mtcomm.2025.112070 (2025).

8 Fernandez-Corbaton, I. *et al.* New Twists of 3D Chiral Metamaterials. *Advanced Materials* **31**, 1807742, doi:https://doi.org/10.1002/adma.201807742 (2019).

9 Hsu, W.-T. *et al.* Second Harmonic Generation from Artificially Stacked Transition Metal Dichalcogenide Twisted Bilayers. *ACS Nano* **8**, 2951-2958, doi:10.1021/nn500228r (2014).

10 Jung, G.-H., Yoo, S. & Park, Q.-H. Measuring the optical permittivity of two-dimensional materials without a priori knowledge of electronic transitions. *Nanophotonics* **8**, 263-270, doi:doi:10.1515/nanoph-2018-0120 (2019).

11 Hsu, C. *et al.* Thickness-Dependent Refractive Index of 1L, 2L, and 3L MoS2, MoSe2, WS2, and WSe2. *Advanced Optical Materials* **7**, 1900239, doi:https://doi.org/10.1002/adom.201900239 (2019).

12 Hirth, J. P., Lothe, J. & Mura, T. Theory of dislocations. *Journal of Applied Mechanics* **50**, 476-477 (1983).

13 Quan, J. *et al.* Phonon renormalization in reconstructed MoS2 moiré superlattices. *Nature Materials* **20**, 1100-1105, doi:10.1038/s41563-021-00960-1 (2021).

14 Bandaru, N. *et al.* Structural stability of WS2 under high pressure. *International Journal of Modern Physics B* **28**, 1450168, doi:10.1142/s0217979214501689 (2014).

15 Zhu, S., Annevelink, E., Pochet, P. & Johnson, H. T. Selection rules of twistronic angles in two-dimensional material flakes via dislocation theory. *Physical Review B* **103**, 115427, doi:10.1103/PhysRevB.103.115427 (2021).

16 Zhang, Z. & Zhu, S. Universal scaling laws on the rotational energy landscape for twisted van der Waals bilayers. *Nanoscale* **17**, 8515-8523, doi:10.1039/D4NR04493B (2025).

17 Zhu, S., Pochet, P. & Johnson, H. T. Controlling Rotation of Two-Dimensional Material Flakes. *ACS Nano* **13**, 6925-6931, doi:10.1021/acsnano.9b01794 (2019).

18 Zhao, Y. *et al.* Supertwisted spirals of layered materials enabled by growth on non-Euclidean surfaces. *Science* **370**, 442-445, doi:doi:10.1126/science.abc4284 (2020).

19 Shearer, M. J. *et al.* Complex and Noncentrosymmetric Stacking of Layered Metal Dichalcogenide Materials Created by Screw Dislocations. *Journal of the American Chemical Society* **139**, 3496-3504, doi:10.1021/jacs.6b12559 (2017).

20 Zhao, Y. & Jin, S. Stacking and Twisting of Layered Materials Enabled by Screw Dislocations and

Non-Euclidean Surfaces. *Accounts of Materials Research* **3**, 369-378, doi:10.1021/accountsmr.1c00245 (2022).

21 Ci, P. *et al.* Quantifying van der Waals Interactions in Layered Transition Metal Dichalcogenides from Pressure-Enhanced Valence Band Splitting. *Nano Letters* **17**, 4982-4988, doi:10.1021/acs.nanolett.7b02159 (2017).

22 Mobaraki, A., Kandemir, A., Yapicioglu, H., Gülseren, O. & Sevik, C. Validation of inter-atomic potential for WS2 and WSe2 crystals through assessment of thermal transport properties. *Computational Materials Science* **144**, 92-98, doi:https://doi.org/10.1016/j.commatsci.2017.12.005 (2018).

23 Ding, Z., Jiang, J.-W., Pei, Q.-X. & Zhang, Y.-W. In-plane and cross-plane thermal conductivities of molybdenum disulfide. *Nanotechnology* **26**, 065703, doi:10.1088/0957-4484/26/6/065703 (2015).
