# Supplementary material for: Twist degree of freedom activated by pressure-driven interlayer engineering in spiral WS2
Source: Nat Commun. 2026 Sep 16;17:9928. doi: 10.1038/s41467-026-77227-3 (PMC13586321; doi:10.1038/s41467-026-77227-3)
Supplement: Supplementary file 2 [file 41467_2026_77227_MOESM2_ESM.pdf]

nature portfolio

# Peer Review File

# Twist degree of freedom activated by pressure-driven interlayer engineering in spiral WS2

Corresponding Author: Professor Junqiao Wu

**This file contains all reviewer reports in order by version, followed by all author rebuttals in order by version.**

Version 0:

Reviewer comments:

Reviewer #1

(Remarks to the Author)
This manuscript reports a pressure-activated twist response in screw-dislocation-driven spiral $WS_2$, probed primarily by polarization-resolved SHG and supported by Raman analysis and theoretical modeling. By linking interlayer compression, metastable stacking, and rotational reconstruction, the work proposes an intriguing route toward in-situ control of twist in multilayer van der Waals systems. Overall, the study is timely and conceptually interesting. The experimental observations are compelling, and the proposed mechanism is thought provoking. However, several physical and conceptual issues would benefit from further clarification before the manuscript can be considered for publication in its present form.

1. The interpretation of the pressure-induced SHG polar rotation as an average interlayer twist across the entire multilayer stack appears to rely on an implicit assumption that the rotational deformation is smoothly distributed along the thickness, with the bottom portion serving as an effectively fixed reference. This point deserves further clarification. Under compression, does the twist develop in a nearly uniform layer-by-layer manner, or is it concentrated at only a few interfaces through highly nonuniform reconstruction? Pressure-dependent SHG measurements on regions of different thickness within the same sample would be particularly informative.

2. Figure 5d correlates interlayer distance with twist angle using an initially ideal 3R reference. However, the spiral $WS_2$ studied here is described as a slipped, metastable interface with translation disorder, rather than a perfect 3R stack. This raises an important conceptual question: for such a non-ideal interfacial registry, how should the interlayer distance vs. twist angle relation be expected to evolve? In particular, does the reconstructed metastable landscape still favor a well-defined minimum interlayer spacing at a specific twist angle?

3. The proposed reversibility is physically intriguing, yet it also raises an important question. If compression drives the system into a pressure-stabilized local minimum, why does unloading restore the initial orientation rather than leave the interface trapped in another metastable state? Given the structural complexity of the spiral multilayer, a fully quantitative model may be unrealistic. Nevertheless, the authors should provide a clearer physical picture for why the process is expected to be intrinsically reversible, rather than history dependent or partially irreversible.

4. The extension of the present results to a beyond Cauchy or effective micropolar-elasticity framework is conceptually appealing, but the theoretical basis remains underdeveloped. In its current form, it is unclear whether the micropolar language is intended as a rigorous effective constitutive description or as a qualitative physical analogy. This distinction should be stated explicitly. If the latter, the language should be moderated accordingly.

5. The manuscript would benefit from a more consistent use of stacking terminology. At present, terms such as “parallel stacking,” “AA stacking,” “parallel, noncentrosymmetric stacking,” and “non-3R” appear to be used with partially overlapping meanings. These descriptors are not equivalent: parallel stacking specifies relative in-plane orientation, whereas AA stacking implies a much more specific atomic registry. Given the authors’ own emphasis that interlayer translations fluctuate strongly, the current wording risks overstating the degree to which an ideal AA configuration has been structurally established.

6. The SHG analysis appears to involve two distinct levels of inference that should be more clearly separated in the manuscript. The experimentally observed quantity is the pressure-induced rotation of the SHG polar pattern, whereas the

reported value of about 0.2° per layer is a model-dependent quantity extracted from a multilayer SHG framework. At present, the wording risks giving the impression that the layer-resolved twist is measured directly. It would be important to distinguish more explicitly between the direct optical observable and the structural parameter inferred from modeling.

Reviewer #2

(Remarks to the Author)
The authors have experimentally compressed the screw-dislocation-driven (SDD) multilayer spiral $WS_2$ via diamond-anvil-cell (DAC) system to achieve the in-situ and reversible tunning of interlayer twist in the three-dimensional (3D) moiré crystals, and theoretically elucidate the mechanism of this compression-twist coupling from an atomic reconstruction and a spontaneous rotational torque by a moiré-based model. Below are a few lingering concerns that need to be addressed to improve the quality, completeness, and depth of the work.
1. The AFM image in Fig. 2d has a low resolution. To emphasize the central spiral, the authors likely performed a second- or third-order flattening on the original height image, resulting in abnormally higher heights on the left and right sides. The shape of the spiral should gradually decrease from the center outwards. It is recommended that the authors provide a clearer AFM height image and a height image with only a first-order flattening in supporting information (SI). Additionally, this image lacks a color bar.
2. Do you have a top-view STEM image of the spiral $WS_2$? The atomic lattice arrangement from this angle would illustrate from another perspective that it's not a ground-state 3R stacking, but rather the parallel stacking the authors described.
3. In line 118, the authors claimed, "This motif is expected to balance a modest increase in interlayer vdW energy against a reduction in elastic strain energy…". Is this argument based on existing knowledge? Does it explain how the interlayer energy and elastic strain energy change in parallel stacking? Are there any references? Is model calculation necessary, and should it be compared with 3R to illustrate its equilibrium effect.
4. The SHG laser spot diameter, using the 50x lens, tested on the spiral $WS_2$ surface and its corresponding size in the AFM image should be given, since the central highest point of the spiral is much smaller than the laser spot.
5. In the SHG in-situ compression and decompression experiments in Fig. 3f, data points during the pressure reduction process also need to be provided to compare whether 1 GPa is also a point of abrupt change in pressure reduction.
6. In line 177-178, the authors claimed, "…whereas the hexagonal sample exhibited no measurable SHG response, indicating centrosymmetric 2H stacking". This description lacks SHG signal response intensity data for the 2H region surrounding the spiral $WS_2$. Furthermore, the 2H stacked TMDs samples do not lack SHG signal; rather, the even-numbered layers cancel each other out, while the odd-numbered layers exhibit it. The authors are requested to provide a more rigorous description.
7. In line 189-192, the authors claimed, "At small twist angles (typically below 2°), large moiré supercells…(Fig. 5c)". However, Fig. 5c is 3°, not less than 2°. Supporting evidence of a value less than or equal to 2° is required.
8. The authors need to provide the goodness-of-fit values for Fig. 4b, especially for the Spiral data, showing the difference between the goodness-of-fit values for two-segment and full-segment fittings, and providing the confidence bands for the fit. This is because fitting each partition at around 1 Gpa is too subjective, and the data points are also suitable for full-segment fitting without partitioning.
9. In line 197-199, the authors claimed, "Above 1 GPa, the interlayer twist saturates and the reconstruction no longer evolves, so the $E_{2g}$ shift mainly reflects conventional hydrostatic response (inset of Fig. 4b)…". However, exceeding 1 GPa, if only the traditional hydrostatic pressure response is considered, the $E_{2g}$ response slopes of Spira and 2H should be the same or similar, not still ~20% larger. Please connect this to the artificial fitting problem I raised above (No. 8).
10. In line 221-223, the author explains the principle of Cauchy linear elasticity. However, references should be included. Additionally, the author could add $\sigma_{ij}$ and $\varepsilon_{kl}$ into Fig. 1a to improve the readability of the formula. Also, in line 223-229, the description is too theoretical. It would be better to add formulas and corresponding diagrams to SI to make it easier for readers to understand.
11. Why not use the parallel registry parameters measured by the STEM for moiré-based modeling (Fig. 5)? Furthermore, a triangular $WS_2$ bilayer structure is too simplistic compared to the complexity of a spiral structure. It should be expanded to at least three layers.
Minor issues:
1. The discission on the behavior and applications of 3D multilayer moiré superstructures (lines 45-49) lacks an aspect and citations regarding the conductive properties of spiral TMDs materials.
2. The authors should clearly indicate which figure and section in SI corresponds to "(Supporting Information)" in the whole manuscript. Please check the entire manuscript against this information and add specific details in parentheses if only SI is mentioned.
3. The current image resolution is not high enough and needs to be improved. Also, the overall aesthetics of the graphics need to be enhanced. For example, the broken axis in Fig. 4a is too tilted.
4. In line 209-211, the authors claimed, "Previous studies…(Fig. 5c)…". However, this part of the article mainly describes the Raman data in Fig. 4, so Fig. 5c should be the result of the research in the following. The author needs to improve the logical consistency of the writing.
5. In line 209-211, the authors claimed, "…a periodic moiré pattern with alternating high-energy (red) and low-energy (blue) domains…". However, in Fig. 5a, the low-energy domains should be described as purple, which is not blue. Also, Fig. 5a and Fig. S7b are the same. Please delete the redundant data.
6. In SI, the authors need to supplement the type of scanning probe model and basic data in the section of AFM characterization.

Reviewer #3

(Remarks to the Author)
The authors d applied longitudinal stress to the spiral WS2 using DAC. Through polarization-dependent SHG and Raman spectroscopy, they observed reversible tuning of interlayer twist up to an average of 0.2° per layer at a pressure of approximately 1 GPa. However, I cannot recommend its publication in NC due to the following reasons, or a major revision is seriously needed for any further consideration.
1, There is no evidence from low-frequency Raman spectroscopy (shear modes and breathing modes), and the small changes in high-frequency Raman are insufficient to indicate variations in interlayer coupling upon interlayer rotation and compression.

2, The analysis of high-frequency Raman is insufficient; in particular, the pressure-dependent frequency difference between the spiral and 2H samples are not explained in detail.

3, The preparation of the spiral $WS_2$ sample is not mentioned. In addition, this sample is not that representative in 2D-related studies.

4, There is no evidence provided for the existence of a moiré potential in the spiral sample.

5, The separation arrangement of main text and figures in the manuscript makes it particularly uncomfortable for reviewers to read.

6, The authors should discuss the effect from the high-pressure medium (such as compressed diamond, pressure-transmitting medium, hBN, etc.) on the polarization-dependent SHG signal.

7, The authors should also provide a comparative study of the polarization-dependent SHG study for 3R stacking under high pressure.

Version 1:

Reviewer comments:

Reviewer #1

(Remarks to the Author)
The authors addressed all the comments of the reviewer satisfactorily. I now can recommend publication of this paper as it is.

Reviewer #2

(Remarks to the Author)
The authors have answered the questions and concerns I raised in the first round of review.

Reviewer #3

(Remarks to the Author)
The supplementary data provided by the authors largely answered my question. However, I would also like to know the influence of diamond (DAC) on the polarization results during the measurement process regarding the SHG data.

**Open Access** This Peer Review File is licensed under a Creative Commons Attribution 4.0 International License, which permits use, sharing, adaptation, distribution and reproduction in any medium or format, as long as you give appropriate credit to the original author(s) and the source, provide a link to the Creative Commons license, and indicate if changes were made.
In cases where reviewers are anonymous, credit should be given to 'Anonymous Referee' and the source.
The images or other third party material in this Peer Review File are included in the article's Creative Commons license, unless indicated otherwise in a credit line to the material. If material is not included in the article's Creative Commons license and your intended use is not permitted by statutory regulation or exceeds the permitted use, you will need to obtain permission directly from the copyright holder.
To view a copy of this license, visit https://creativecommons.org/licenses/by/4.0/

**Point-by-point response to Reviewer #1's comments:**

**Overall comment:** *This manuscript reports a pressure-activated twist response in screw-dislocation-driven spiral $WS_2$, probed primarily by polarization-resolved SHG and supported by Raman analysis and theoretical modeling. By linking interlayer compression, metastable stacking, and rotational reconstruction, the work proposes an intriguing route toward in-situ control of twist in multilayer van der Waals systems. Overall, the study is timely and conceptually interesting. The experimental observations are compelling, and the proposed mechanism is thought provoking. However, several physical and conceptual issues would benefit from further clarification before the manuscript can be considered for publication in its present form.*

Response:

We thank the reviewer for the positive assessment and constructive comments. We have revised the manuscript to address these issues by spatially resolved SHG measurements and updated theoretical calculations.

**Q1:** *The interpretation of the pressure-induced SHG polar rotation as an average interlayer twist across the entire multilayer stack appears to rely on an implicit assumption that the rotational deformation is smoothly distributed along the thickness, with the bottom portion serving as an effectively fixed reference. This point deserves further clarification. Under compression, does the twist develop in a nearly uniform layer-by-layer manner, or is it concentrated at only a few interfaces through highly nonuniform reconstruction? Pressure-dependent SHG measurements on regions of different thickness within the same sample would be particularly informative.*

Response:

We thank the reviewer for this important comment. We agree that the polarization-resolved SHG measurement does not directly resolve the twist angle of each individual interface. Rather, it provides an effective relative reorientation of the optically probed multilayer region with respect to the crystallographic edge reference.

To address the requested spatial dependence, we performed pressure-dependent polarization-resolved SHG measurements at two distinct positions of the same spiral $WS_2$ crystal (Fig. R1). Position 1 is closer to the spiral center, whereas Position 2 is farther from the center. At both positions, the SHG pattern rotation increases upon compression and approaches saturation near 1 GPa, consistent with the behavior observed in main-text Fig. 3. However, the saturated rotation is position dependent: Position 1 reaches about 1.7° to 1.8°, whereas Position 2 reaches about 1.0° to 1.2°. These results show that the compression-induced rotational response is spatially nonuniform across the spiral crystal. The common crossover pressure near 1 GPa supports a shared compression-twist mechanism, whereas the reduced rotation away from the spiral center is consistent with a smaller number of participating interfaces and/or different local registry and reconstruction conditions.

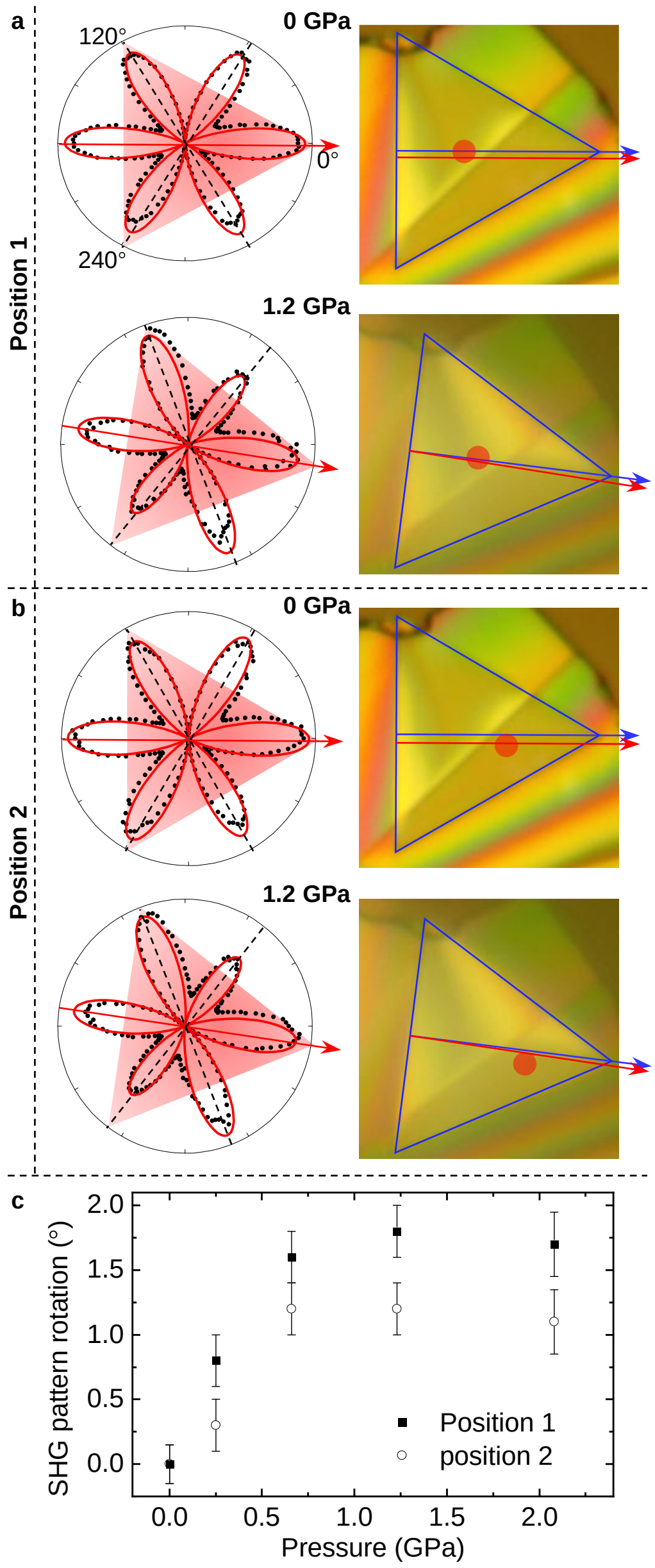


**Fig. R1 Spatial dependence of the pressure-induced SHG pattern rotation in a single spiral $WS_2$ crystal. a-b**, Polarization-resolved SHG patterns acquired at two positions of the same spiral $WS_2$ crystal under 0 and 1.2 GPa. The optical images mark the SHG excitation positions with red circles. Black symbols denote the measured SHG signal, and red curves denote the corresponding fits. Blue arrows indicate the crystallographic edge reference, whereas red arrows indicate the SHG-derived armchair direction. **c**, pressure dependence of the SHG pattern rotation relative to the ambient-pressure orientation for Position 1 (filled squares) and Position 2 (open circles).

**Q2:** *Figure 5d correlates interlayer distance with twist angle using an initially ideal 3R reference. However, the spiral $WS_2$ studied here is described as a slipped, metastable interface with translation disorder, rather than a perfect 3R stack. This raises an important conceptual question: for such a non-ideal interfacial registry, how should the interlayer distance vs. twist angle relation be expected to evolve? In particular, does the reconstructed metastable*

*landscape still favor a well-defined minimum interlayer spacing at a specific twist angle?*

Response:

We thank the reviewer for this important comment. We agree that the original Fig. 5d, which considered only an initially ideal 3R registry, did not directly address the interlayer-distance evolution of the slipped, metastable interfaces observed in spiral $WS_2$.

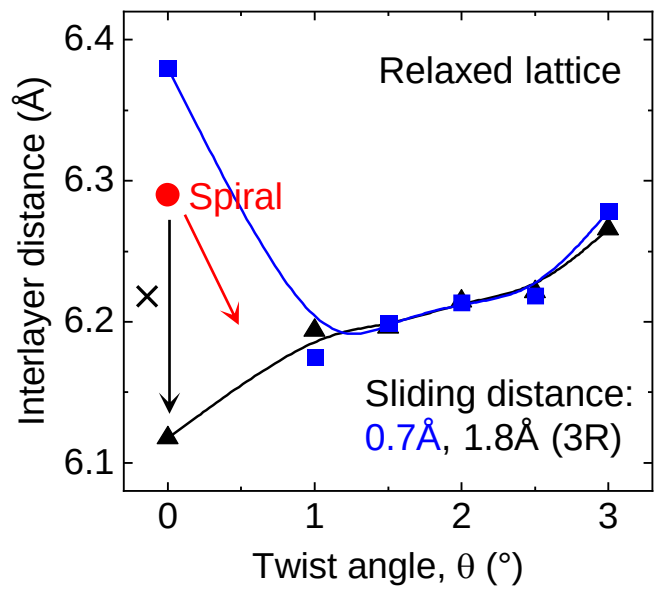


**Fig. R2 Interlayer distance coupled to twist angle in ideal and slipped bilayer $WS_2$.** Equilibrium interlayer distance obtained from relaxed-lattice calculations as a function of twist angle for ideal 3R stacking with an armchair translation of 1.8 Å (black triangles) and a representative slipped parallel configuration with an armchair-projected translation of 0.7 Å (blue squares). Symbols denote discrete calculated energies, and curves are B-spline guides to the eye. The red circle indicates the ambient-pressure interlayer distance measured for spiral $WS_2$ by cross-sectional STEM.

To address this issue, we replaced the original analysis with relaxed-lattice calculations comparing ideal 3R stacking and a representative slipped parallel configuration (Fig. R2). The slipped model was initialized with an armchair-projected interlayer translation of 0.7 Å. For ideal 3R stacking, the equilibrium interlayer distance is smallest at $\theta = 0°$ and increases with finite twist angle, as expected for the ground-state registry[1]. In contrast, the slipped configuration exhibits a sterically expanded interlayer spacing at $\theta = 0°$. Upon introducing a small twist, the equilibrium spacing decreases substantially and reaches a minimum near $\theta = 1.0°$, before increasing again at larger twist angles. Thus, the metastable slipped interface does not follow the same twist-spacing relation as ideal 3R stacking. Instead, finite rotation can simultaneously lower the interlayer spacing and relax the registry-dependent interfacial energy. This result provides a direct microscopic basis for the proposed compression-twist coupling. Under hydrostatic compression, a slipped interface can access a lower-spacing finite-angle configuration without requiring extensive lateral sliding toward the ideal 3R registry.

We emphasize that the multilayer spiral contains a distribution of local interlayer translations, so the specific twist angle associated with the spacing minimum is not expected to be identical for every interface. The calculation instead demonstrates that a representative slipped, metastable registry can possess a finite-angle minimum in both interlayer spacing and rotational energy, supporting the physical feasibility of compression-driven twist in the translation-disordered spiral structure.

Based on the above discussion, we have updated main-text Fig. 6d and its caption in the revised manuscript and added the corresponding discussion to the section "Moiré-driven origin of

compression-twist coupling."

**Q3:** *The proposed reversibility is physically intriguing, yet it also raises an important question. If compression drives the system into a pressure-stabilized local minimum, why does unloading restore the initial orientation rather than leave the interface trapped in another metastable state? Given the structural complexity of the spiral multilayer, a fully quantitative model may be unrealistic. Nevertheless, the authors should provide a clearer physical picture for why the process is expected to be intrinsically reversible, rather than history dependent or partially irreversible.*

Response:

We thank the reviewer for this important question. We agree that the reversibility requires a clearer physical picture. In our interpretation, the finite-angle state reached under compression is not an independent metastable state that remains energetically preferred after pressure release. Instead, it is a pressure-conditioned local minimum created by the compression-induced reduction in interlayer spacing and the accompanying change in the registry-dependent interfacial energy landscape.

Upon unloading, the interlayer spacing expands and the reconstructed energy landscape evolves back toward its ambient-pressure form. The finite-angle minimum is therefore expected to shift toward, or merge with, the initial slipped configuration. The screw-dislocation-driven spiral further retains an intrinsic elastic restoring contribution associated with its built-in strain field, continuous terrace geometry, and local registry constraints. These factors favour recovery toward the initial relative orientation rather than unrestricted trapping at a new rotated state. Consistent with this picture, the SHG pattern rotation decreases during decompression and the initial orientation is recovered after full pressure release, as shown in main-text Fig. 3f.

**Q4:** *The extension of the present results to a beyond Cauchy or effective micropolar-elasticity framework is conceptually appealing, but the theoretical basis remains underdeveloped. In its current form, it is unclear whether the micropolar language is intended as a rigorous effective constitutive description or as a qualitative physical analogy. This distinction should be stated explicitly. If the latter, the language should be moderated accordingly.*

Response:

We thank the reviewer for this important clarification. We agree that the original text did not sufficiently distinguish between a rigorous micropolar constitutive description and a qualitative physical analogy. Our intention is the latter. We do not derive, fit, or extract effective micropolar elastic constants, couple-stress parameters, or force-rotation coupling tensors for spiral $WS_2$.

We have therefore moderated the corresponding discussion to clarify that the Cauchy and micropolar frameworks are introduced only to provide continuum-mechanics context for the existence of an internal rotational degree of freedom. The microscopic basis of our interpretation remains the calculated registry-dependent interfacial energy landscape, its pressure-dependent reconstruction, and the resulting interlayer rotational torque. The revised text reads:

Within Cauchy linear elasticity, the stress $\sigma_{ij}$ is related to the symmetric strain $\varepsilon_{kl}$ via $\sigma_{ij} = C_{ijkl}$

$\varepsilon_{kl}$. Because $C_{ijkl}$ is invariant under space inversion, this homogeneous description contains no independent internal rotational degree of freedom under pure compression[2]. Micropolar elasticity extends this framework by introducing microrotation and couple-stress fields, allowing such coupling in micropolar media through additional force-rotation terms (Fig. R15)[2-4]. For spiral $WS_2$, we use this framework only as a conceptual effective-medium analogy. The translation-disordered interfaces can be viewed as an effective micropolar interfacial medium, introducing local registry and relative-rotation coordinates that are absent from a homogeneous Cauchy description. At the microscopic level, we propose that compression activates this rotational degree of freedom by modifying the moiré energy landscape through atomic reconstruction, thereby generating an interlayer rotational torque. Spiral $WS_2$ therefore provides an atomic-scale route to pressure-tunable compression-twist coupling through interfacial engineering.

We have added the above discussion to the "Moiré-driven origin of compression-twist coupling" section in the main text and to Supplementary Fig. 15.

**Q5:** *The manuscript would benefit from a more consistent use of stacking terminology. At present, terms such as "parallel stacking," "AA stacking," "parallel, noncentrosymmetric stacking," and "non-3R" appear to be used with partially overlapping meanings. These descriptors are not equivalent: parallel stacking specifies relative in-plane orientation, whereas AA stacking implies a much more specific atomic registry. Given the authors' own emphasis that interlayer translations fluctuate strongly, the current wording risks overstating the degree to which an ideal AA configuration has been structurally established.*

Response:

We thank the reviewer for this important clarification. We agree that the original terminology did not sufficiently distinguish relative layer orientation, local interlayer registry, and the noncentrosymmetric optical response inferred from SHG.

We have therefore revised the terminology throughout the manuscript. "Parallel orientation" is now used only to indicate that adjacent $WS_2$ layers retain the same in-plane crystallographic orientation, in contrast to the 60°-rotated 2H sequence. The local stacking of spiral $WS_2$ is described as a parallel-oriented, translation-disordered registry, reflecting the fluctuating interlayer translations resolved by cross-sectional and plan-view STEM (main-text Fig. 2b and Supplementary Fig. 9-11).

We have removed the term "AA stacking" from the manuscript. In particular, the local high-energy nodes in the reconstructed moiré model are now denoted as MMXX nodes, without assigning them to an ideal AA registry. This change avoids implying that an exact AA configuration is structurally established in the spiral multilayer.

Q6: *The SHG analysis appears to involve two distinct levels of inference that should be more clearly separated in the manuscript. The experimentally observed quantity is the pressure-induced rotation of the SHG polar pattern, whereas the reported value of about 0.2° per layer is a model-dependent quantity extracted from a multilayer SHG framework. At present, the wording risks giving the impression that the layer-resolved twist is measured directly. It would be important to distinguish more explicitly between the direct optical observable and the*

*structural parameter inferred from modeling.*

Response:

We thank the reviewer for this important clarification. We agree that the original wording did not sufficiently distinguish the directly measured optical quantity from the structural parameter inferred through modelling.

The direct experimental observable is the pressure-dependent rotation of the fitted SHG polar pattern relative to the crystallographic reference direction determined from the spiral edges. This measurement establishes a pressure-induced reorientation of the optically probed multilayer region, but it does not directly resolve the twist angle of each individual interface. The value of approximately 0.2° per layer is instead obtained from a multilayer SHG field-superposition model. It represents an effective average interlayer rotation estimated under the model assumption that the total relative reorientation is distributed among the contributing interfaces. It should therefore not be interpreted as a direct layer-resolved measurement or as evidence that every interface undergoes the same rotation.

We have revised the manuscript accordingly.

**Point-by-point response to Reviewer #2's comments:**

**Overall comment:** *The authors have experimentally compressed the screw-dislocation-driven (SDD) multilayer spiral $WS_2$ via diamond-anvil-cell (DAC) system to achieve the in-situ and reversible tunning of interlayer twist in the three-dimensional (3D) moiré crystals, and theoretically elucidate the mechanism of this compression-twist coupling from an atomic reconstruction and a spontaneous rotational torque by a moiré-based model. Below are a few lingering concerns that need to be addressed to improve the quality, completeness, and depth of the work.*

Response:

We thank the reviewer for the constructive evaluation and helpful suggestions. In the revised manuscript, we have addressed the remaining concerns by adding plan-view STEM evidence, reprocessing the AFM data, expanding the small-angle and trilayer calculations, and including Raman analysis of the in-plane vibrational response.

**Q1:** *The AFM image in Fig. 2d has a low resolution. To emphasize the central spiral, the authors likely performed a second- or third-order flattening on the original height image, resulting in abnormally higher heights on the left and right sides. The shape of the spiral should gradually decrease from the center outwards. It is recommended that the authors provide a clearer AFM height image and a height image with only a first-order flattening in supporting information (SI). Additionally, this image lacks a color bar.*

Response:

We thank the reviewer for this constructive suggestion. We agree that the original Fig. 2d did not present the spiral topography with sufficient clarity.

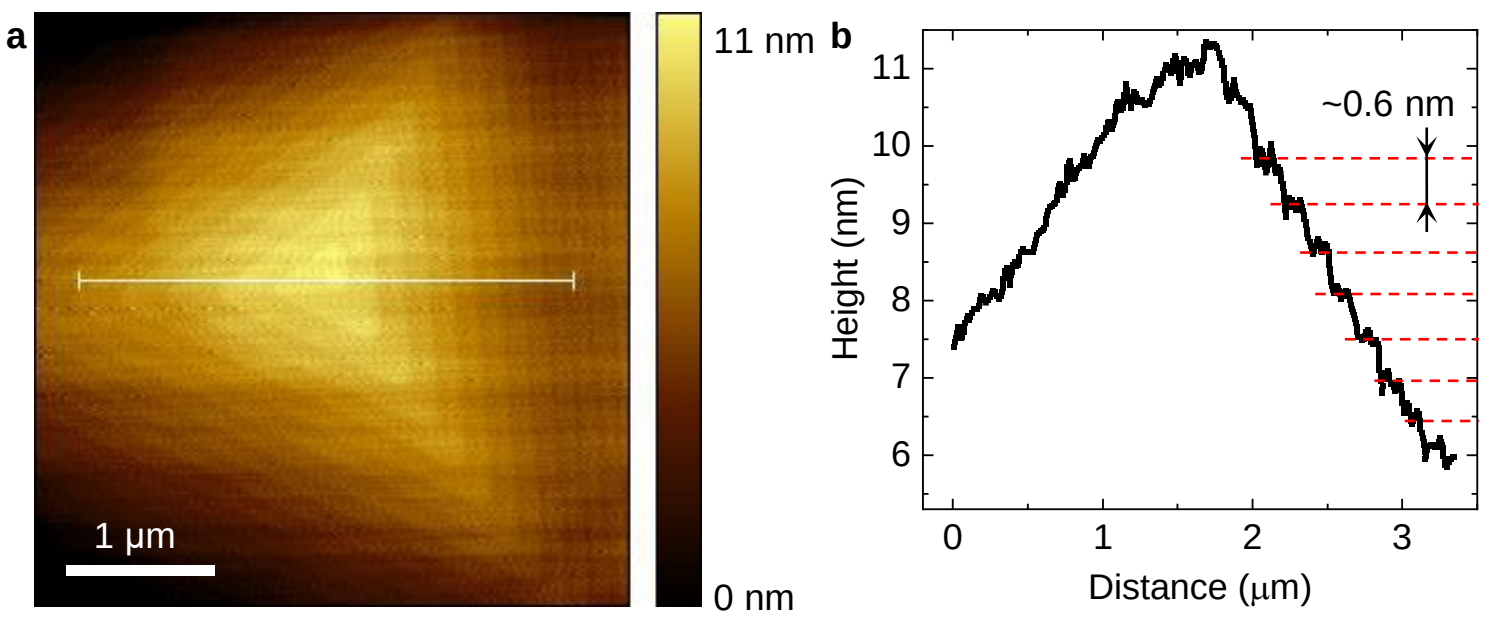


**Fig. R3 First-order-flattened AFM topography of the spiral $WS_2$ crystal. a**, AFM height image of the spiral $WS_2$ crystal after first-order plane flattening. The color scale indicates the sample height. The white line marks the position used to extract the height profile. **b**, Height profile across the spiral core. The step height is about 0.6 nm, consistent with monolayer $WS_2$ terraces.

We have reprocessed the AFM height data with a clearer height map obtained using first-order plane flattening only (Fig. R3). No second- or third-order flattening was applied. As suggested, we provide the corresponding first-order-flattened height image (Fig. R3a), including a color

bar, together with a height profile across the spiral core (Fig. R3b). The revised map and profile show that the spiral core is the highest region, while the overall height envelope decreases from the core toward the outer terraces, consistent with our previous reports[5-7]. The profile further resolves discrete steps of about 0.6 nm, consistent with monolayer $WS_2$ terraces. These data provide a clearer visualization of the spiral terraces surrounding the central core and their aligned edges.

The above discussion has been incorporated into Fig. 2d in the main text and Supplementary Fig. 8.

**Q2:** *Do you have a top-view STEM image of the spiral $WS_2$? The atomic lattice arrangement from this angle would illustrate from another perspective that it's not a ground-state 3R stacking, but rather the parallel stacking the authors described.*

Response:

We thank the reviewer for this valuable suggestion. We agree that a plan-view STEM image can provide important complementary structural information and help distinguish a regular ground-state 3R registry from a parallel stacking configuration. To provide this independent structural view of the multilayer spiral $WS_2$, we have acquired plan-view atomic-resolution HAADF-STEM images from multiple regions of the same spiral crystal, together with a focal series collected from the same local region.

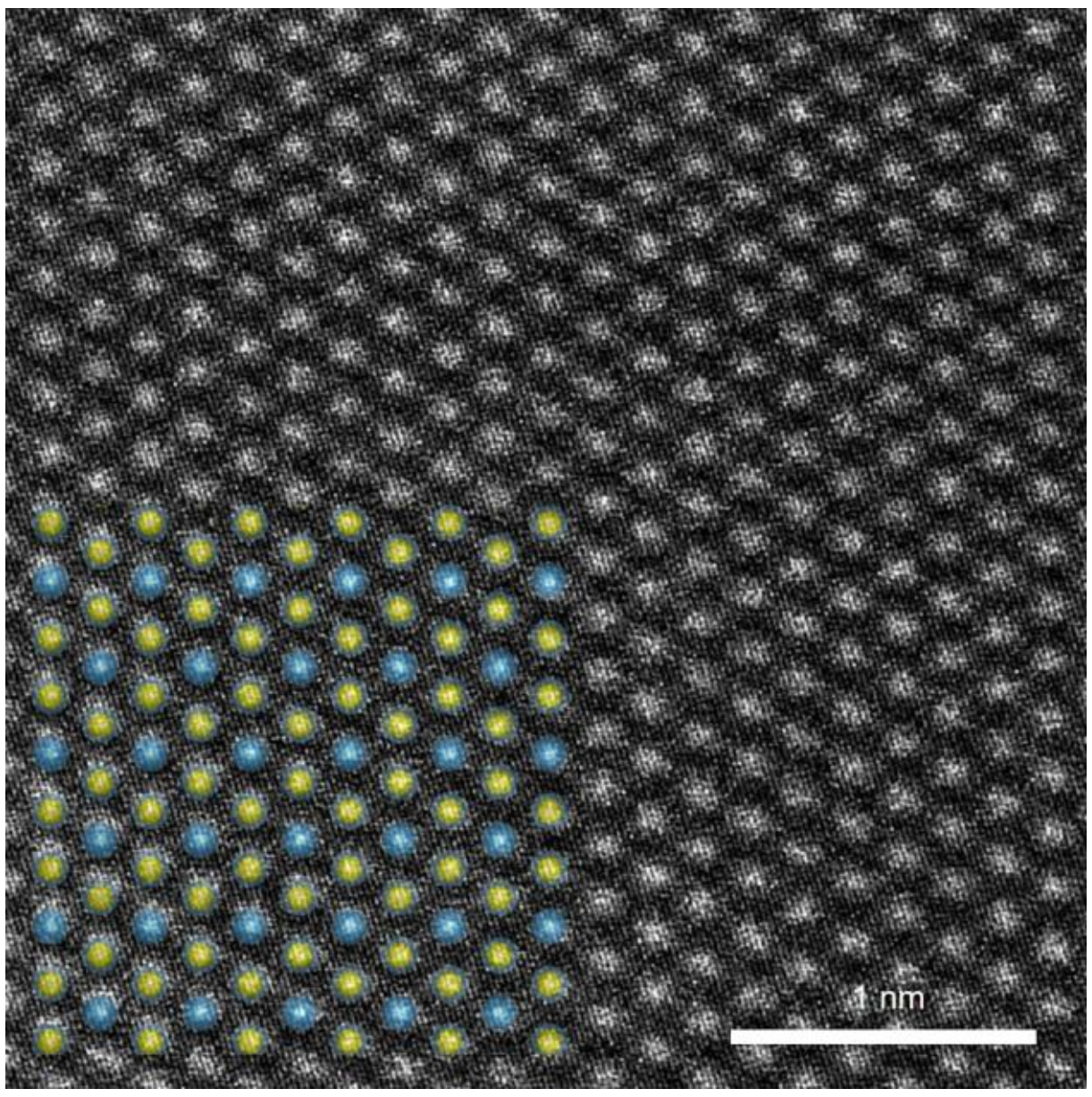


**Fig. R4 Plan-view atomic-resolution HAADF-STEM reference of ideal 3R stacking.** The regular projected atomic pattern of exfoliated 3R $MoS_2$ is consistent with the periodic rhombohedral registry expected for ideal 3R stacking. The overlaid model identifies representative projected W and S sublattices. Scale bar, 1 nm.

As a qualitative structural reference for ideal 3R registry, we also measured an exfoliated 3R $MoS_2$ specimen (Fig. R4). The 3R reference displays a regular projected atomic pattern with nearly uniform contrast, consistent with the periodic rhombohedral stacking sequence[8,9]. This dataset is used only as a structural reference for ideal 3R registry, rather than for a quantitative

comparison of absolute HAADF intensities between $MoS_2$ and $WS_2$.

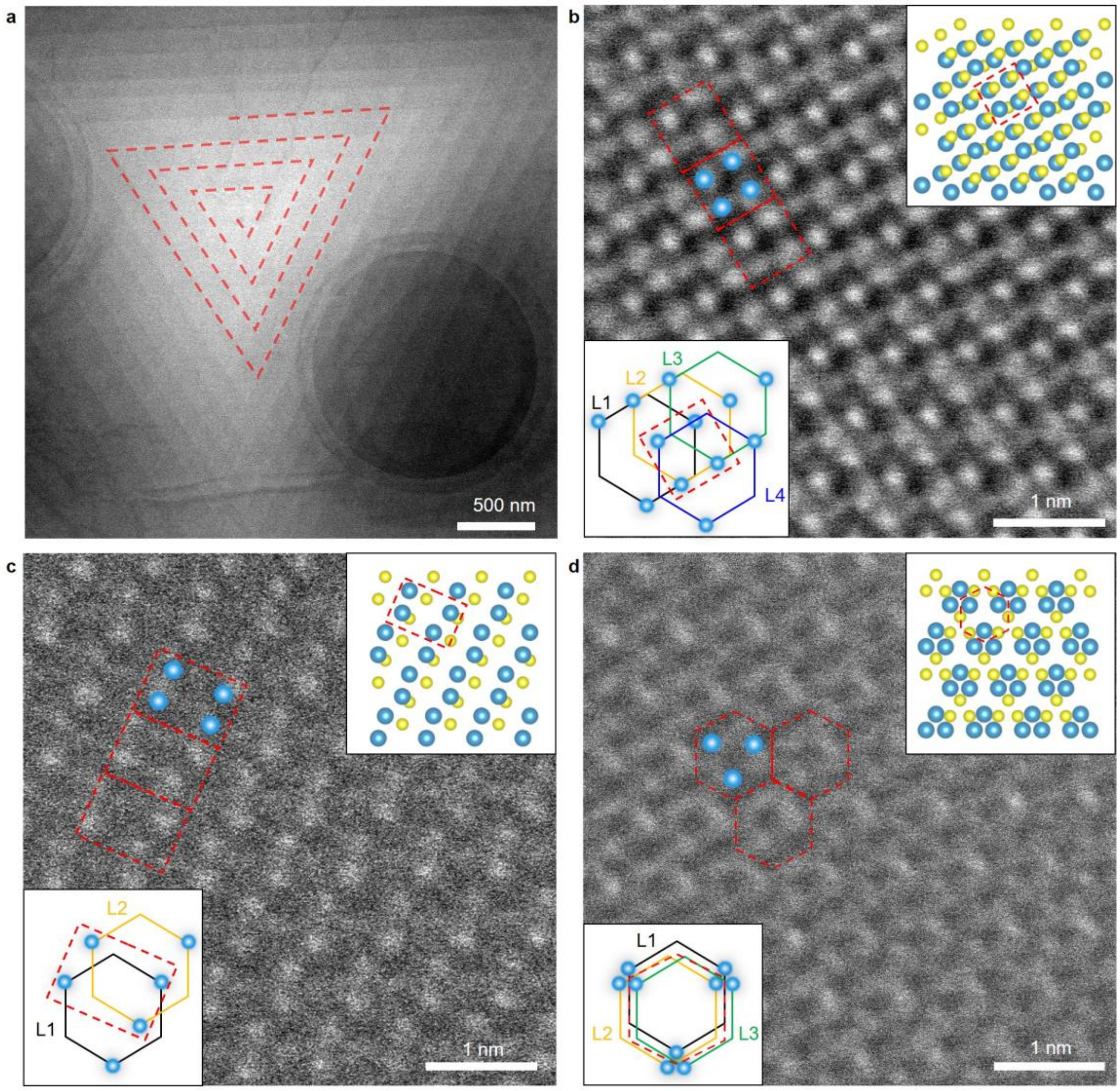


**Fig. R5 Plan-view HAADF-STEM evidence for spatially varying local registry in spiral $WS_2$. a**, Low-magnification plan-view STEM image of a multilayer spiral $WS_2$. Red dashed lines trace the spiral terraces. **b-d**, Atomic-resolution HAADF-STEM images acquired from different regions of the same spiral crystal, revealing distinct projected atomic motifs. Blue circles mark selected projected W-containing columns. The upper-right insets show projected W/S arrangements, and the lower-left insets show minimal illustrative models comprising four (**b**), two (**c**), and three (**d**) parallel-oriented $WS_2$ layers, respectively. Red dashed outlines identify corresponding local motifs in the experimental images and models. These models illustrate how nonuniform interlayer translations can qualitatively reproduce the observed projections and are not unique structural reconstructions. Scale bars, 500 nm in a and 1 nm in **b-d**.

Low-magnification TEM image of the overall spiral structure (Fig. R5a) shows that the edge atoms are well aligned, consistent with the AFM observations (Fig. 2d in the main text), indicating a parallel stacking relationship between layers. Unlike regular 3R stacking, the plan-view HAADF-STEM images of spiral $WS_2$ reveal spatially varying projected atomic configurations at different locations within the same multilayer spiral crystal (Fig. R5b-d).

These local motifs are inconsistent with a uniform ideal 3R sequence. Because this comparison is based on projected contrast, it illustrates translation-disordered registry but does not uniquely determine the translation vector at each interface. Combined with the cross-sectional STEM results (Fig. 2b in the main text), which establish a common in-plane orientation of adjacent layers, the plan-view observations provide independent, spatially resolved evidence for translation-disordered interlayer registry among parallel-oriented $WS_2$ layers (Fig. R5). This result supports the central structural picture of the manuscript: the spiral crystal retains parallel orientation but contains spatially nonuniform lateral sliding rather than a regular ground-state 3R stacking sequence.

To aid interpretation of these representative local motifs, we constructed minimal illustrative projected models comprising four, two, and three parallel $WS_2$ layers for the upper-right, lower-left, and lower-right HAADF-STEM panels (Fig. R5b-d), respectively. In each model, $L_1$, outlined in black, serves as the reference layer, whereas the colored hexagons denote later layers laterally translated relative to $L_1$. The lower-left inset in each panel shows the relative positions of the layer-specific W sublattices, and the upper-right inset shows the corresponding projected W/S arrangement. The red dashed outlines identify equivalent local motifs in the experimental HAADF-STEM images and in the projected structural models, while blue circles mark the projected W-containing columns used for comparison. These nonuniform in-plane translations qualitatively reproduce the distinct projected motifs observed at different locations. We emphasize that this model is intended as a visual illustration rather than a unique structural reconstruction. Different translation combinations or local strain induced by screw dislocation can in principle generate related projected motifs.

We further collected an atomic-resolution HAADF-STEM focal series from the same multilayer spiral $WS_2$ region at different probe defocus values (Fig. R6). Under the present imaging conditions of 300 kV and a 25 mrad convergence semi-angle, the axial response of the STEM probe extends over several nanometres, substantially exceeding the interlayer spacing of $WS_2$. We therefore do not interpret the focal series as direct imaging of individual layers. Rather, changing the probe focus modifies the relative contribution of distinct stacking configurations along the beam direction to the projected HAADF contrast. The resulting focus-dependent change in the apparent atomic arrangement provides additional real-space evidence consistent with depth-dependent interlayer registry and translational disorder.

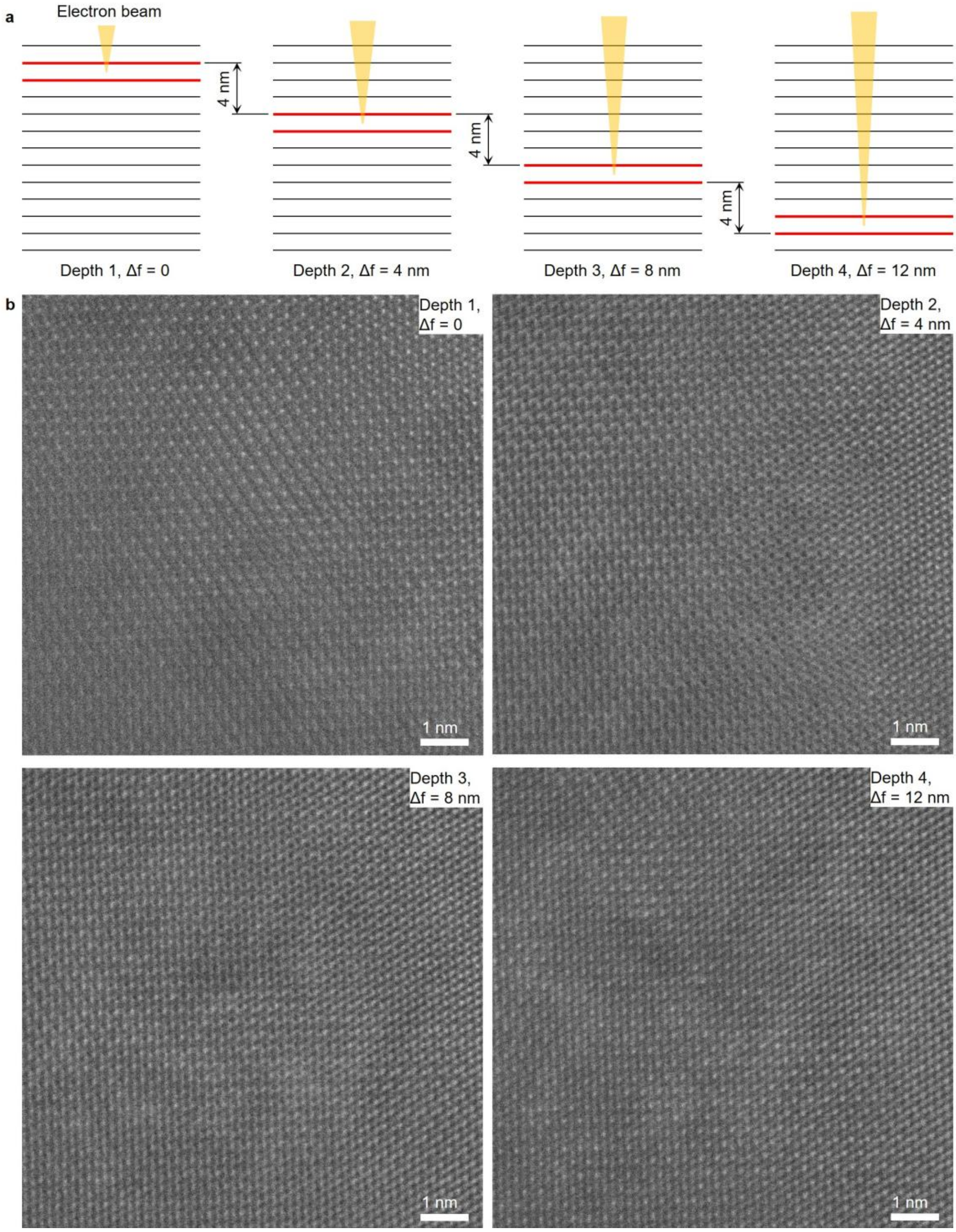


**Fig. R6 Focal-series HAADF-STEM of multilayer spiral $WS_2$. a**, Schematic of the nominal focal conditions used for the focal series. Red lines indicate the nominal depth ranges preferentially weighted at each defocus setting. **b**, Atomic-resolution HAADF-STEM images collected from the same region at $\Delta f$ = 0, 4, 8, and 12 nm. The focus-dependent changes in projected atomic contrast are consistent with depth-dependent interlayer registry and translational disorder. Scale bars, 1 nm.

Together, these plan-view (Fig. R5-6) and cross-sectional STEM (Fig. 2b in the main text) measurements support a parallel-oriented, translation-disordered, non-3R stacking structure in

spiral $WS_2$.

The main text has been revised based on the above discussion, and the corresponding results have been added to Figs. S9-S11 in the Supporting Information.

Q3: *In line 118, the authors claimed, "This motif is expected to balance a modest increase in interlayer vdW energy against a reduction in elastic strain energy…". Is this argument based on existing knowledge? Does it explain how the interlayer energy and elastic strain energy change in parallel stacking? Are there any references? Is model calculation necessary, and should it be compared with 3R to illustrate its equilibrium effect.*

Response:

We thank the reviewer for identifying that our original statement did not sufficiently distinguish the registry-dependent interlayer interaction energy from the elastic contribution associated with the screw-dislocation-induced strain field. We agree that the original wording was too qualitative.

Previous studies of reconstructed van der Waals interfaces have established that the local stacking registry determines both the interlayer interaction energy and the equilibrium interlayer spacing, while the reconstructed configuration is selected through competition with intralayer elastic energy[1,10-12]. To address this issue in the context of spiral $WS_2$, we performed calculations for bilayer $WS_2$, comparing ideal 3R stacking with representative slipped parallel registries as a function of applied uniaxial tensile strain.

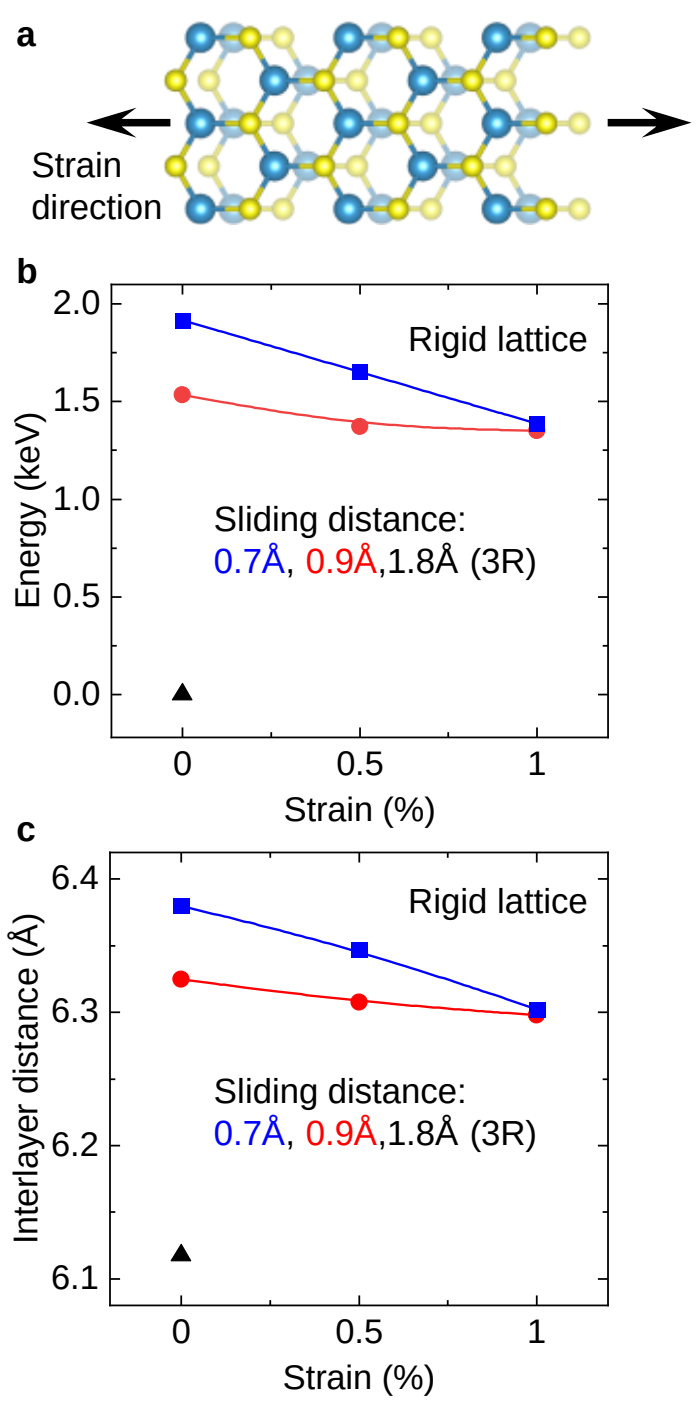


**Fig. R7 Strain-dependent energy and interlayer spacing of rigid slipped bilayer $WS_2$ configurations.** **a**, Schematic of the applied uniaxial in-plane strain along the direction indicated. **b-c**, Calculated total energy and corresponding equilibrium interlayer distances as a function of strain for bilayer $WS_2$ with fixed lateral sliding distances of 0.7 Å (blue) and 0.9 Å (red). The black triangle denotes the ideal 3R reference configuration, corresponding to a 1.8

Å lateral translation.

The calculations show that the slipped configurations have a less favourable registry-dependent interlayer interaction energy and a larger equilibrium spacing than ideal 3R stacking. Importantly, the uniaxial strain field lowers the total energy and interlayer distance of the slipped configurations relative to their unstrained counterparts. This allows screw-dislocation-induced strain to stabilize non-3R registries as energetically accessible metastable configurations despite their higher energy than ideal 3R stacking.

The above discussion has been incorporated into Supplementary Fig. 12.

**Q4.** *The SHG laser spot diameter, using the 50x lens, tested on the spiral $WS_2$ surface and its corresponding size in the AFM image should be given, since the central highest point of the spiral is much smaller than the laser spot.*

Response:

We thank the reviewer for raising this important spatial-resolution issue.

We calibrated the SHG excitation spot using a knife-edge scan under the same 50× objective and optical configuration employed for the pressure-dependent measurements, obtaining a spot diameter of 1.5-2 μm. We have added an AFM topography image overlaid with the calibrated spot size in Fig. R8. Because the highest central region is smaller than the excitation spot, the measured SHG signal represents an area-weighted response from the central region and neighbouring spiral terraces rather than an isolated response from the central apex.

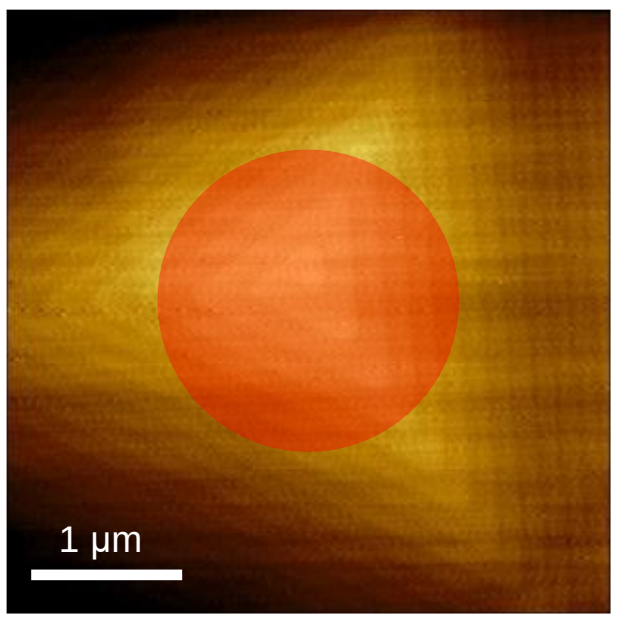


**Fig. R8 Spatial extent of the SHG excitation spot (shaded circle) relative to the spiral $WS_2$ core.** Scale bar, 1 μm.

The neighbouring terraces are contiguous parts of the same multilayer spiral crystal. Therefore, although spatial averaging prevents us from assigning the measured response to a single terrace, it does not preclude the observed pressure-induced SHG rotation from reflecting a collective compression-twist response of the probed spiral region.

The above discussion has been incorporated into the Methods section of the Supporting Information.

**Q5:** *In the SHG in-situ compression and decompression experiments in Fig. 3f, data points during the pressure reduction process also need to be provided to compare wheth1er 1 GPa is also a point of abrupt change in pressure reduction.*

Response:

We thank the reviewer for this important suggestion.

We have added SHG measurements acquired during the original pressure cycle at 1.2 GPa and 0.5 GPa upon decompression (Fig. R9) for the same sample shown in main-text Fig. 3, together with the measurement after full pressure release. The unloading data show a reduction of the SHG pattern rotation as pressure is released, and the initial orientation is recovered after decompression to ambient pressure, supporting a reversible compression-twist response.

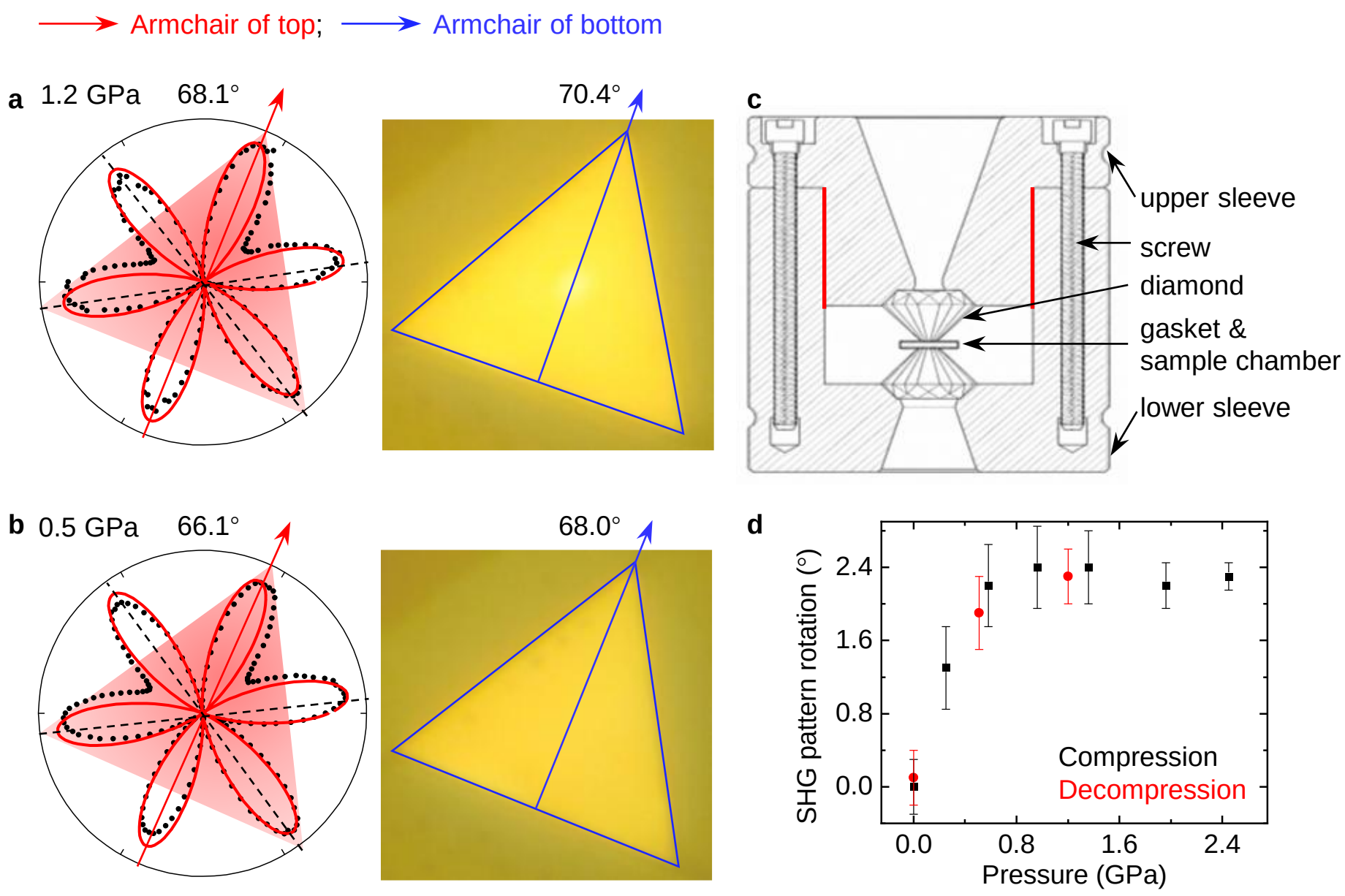


**Fig. R9 SHG response during decompression of spiral $WS_2$. a-b,** Polarization-resolved SHG patterns and corresponding optical images of the spiral $WS_2$ measured during decompression at 1.2 GPa and 0.5 GPa, respectively. Black symbols and red curves denote the measured SHG intensities and corresponding fits. Red arrows indicate the armchair orientation extracted for the top surface from the SHG fits, whereas blue arrows indicate the armchair orientation of the bottom layers inferred from the triangular spiral edges. **c**, Cross-sectional schematic of the diamond-anvil cell. Red lines mark the tightly assembled regions between the upper and lower sleeves, which maintain the alignment and parallelism of the diamond anvils during compression. **d**, SHG pattern rotation as a function of pressure during compression (black) and decompression (red).

We note that the present unloading data do not provide sufficient resolution to define a precise unloading threshold. To maintain the alignment and parallelism of the diamond anvils, the upper and lower sleeves of the diamond-anvil cell were tightly assembled, as marked by the red lines in Fig. R9c. During unloading, the sample pressure was not controlled continuously by screw displacement alone and could relax in discontinuous steps, which prevented reproducible acquisition at equally spaced intermediate pressure values. Nevertheless, within experimental uncertainty, the decompression data overlap with the compression trend at the accessible pressure points. In particular, the 1.2 GPa unloading point is consistent with the saturated response observed above about 1 GPa during compression (Fig. R9d).

*Q6: In line 177-178, the authors claimed, "...whereas the hexagonal sample exhibited no measurable SHG response, indicating centrosymmetric 2H stacking". This description lacks SHG signal response intensity data for the 2H region surrounding the spiral $WS_2$. Furthermore, the 2H stacked TMDs samples do not lack SHG signal; rather, the even-numbered layers cancel each other out, while the odd-numbered layers exhibit it. The authors are requested to provide a more rigorous description.*

Response:

We thank the reviewer for this clarification. We agree that our original wording was overly general, because finite odd-layer 2H TMD stacks can retain a nonzero SHG response, whereas even-layer stacks exhibit electric-dipole SHG cancellation.

To address this point, we have added the original SHG spectra acquired from the spiral and adjacent hexagonal regions under identical measurement conditions. The spiral region exhibits a pronounced SHG peak near 400 nm, whereas the hexagonal region shows no detectable peak above the background level (Fig. R10a-b). We have also added AFM characterization of a separate $WS_2$ flake with closely matched optical contrast (Fig. R10c-d). Its thickness is approximately 16 nm, corresponding to roughly 25 to 26 layers, confirming that the hexagonal reference lies in the thick-multilayer regime rather than the few-layer limit. Previous studies of 2H TMD multilayers have shown that, although odd-layer stacks retain a finite electric-dipole SHG response, the SHG intensity decreases substantially with increasing odd-layer number and becomes strongly suppressed in the bulk limit because of near cancellation between adjacent layers[13]. Given the approximately 16 nm thickness of the comparable hexagonal flake, the absence of a detectable SHG peak from the hexagonal region is therefore consistent with strong compensation of layer-resolved SH fields in a thick 2H-stacked multilayer.

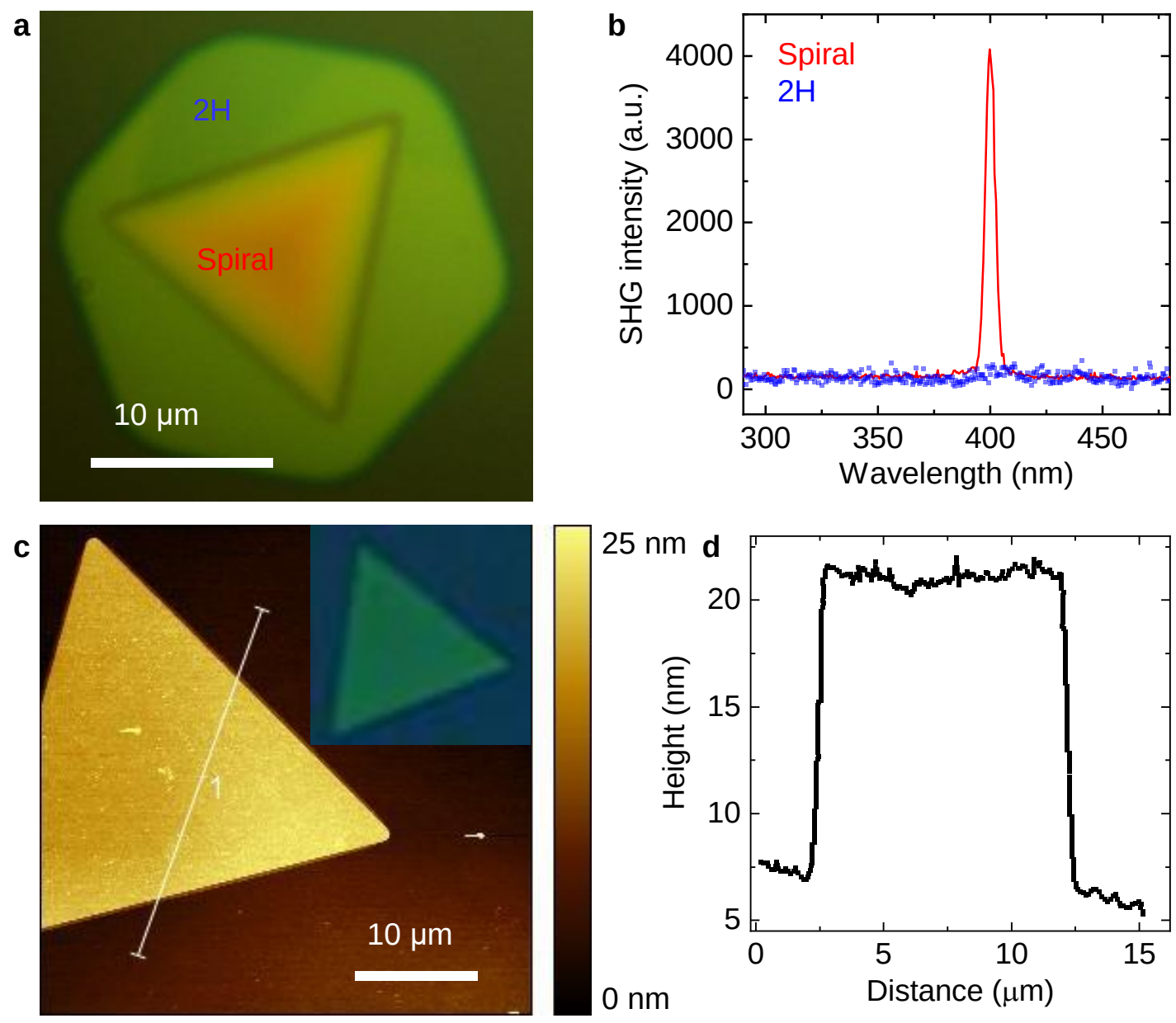


**Fig. R10 SHG response of the hexagonal 2H reference region and thickness characterization of a comparable multilayer flake. a**, Optical image of a spiral $WS_2$ region surrounded by a hexagonal 2H $WS_2$ region. **b**, Original SHG spectra acquired from the spiral

and adjacent hexagonal 2H regions under identical measurement conditions. The spiral region exhibits a pronounced SHG peak near 400 nm, whereas no detectable SHG peak is observed from the hexagonal region above the background level. **c**, AFM topography image of a separate $WS_2$ flake with optical contrast comparable to that of the hexagonal reference region in **a**. Inset, corresponding optical image. **d**, Height profile along the line marked in **c**, showing a flake thickness of approximately 15 nm. Scale bars, 10 μm in **a** and **c**.

The assignment of the hexagonal region as a 2H-stacked reference does not rely solely on the absence of detectable SHG. Previous structural studies have established that conventionally grown hexagonal CVD $WS_2$ flakes adopt the 2H stacking sequence[6,14]. This assignment is further consistent with the characteristic hexagonal morphology of the surrounding region and its negligible SHG response. Because the original hexagonal region was no longer available for AFM measurement, its exact layer number and parity cannot be determined retrospectively, whereas the absence of a detectable peak in the present thick hexagonal multilayer is consistent with strong compensation of layer-resolved SH fields in a 2H-stacked reference.

The above discussion has been incorporated into Supplementary Fig. 5.

**Q7:** *In line 189-192, the authors claimed, "At small twist angles (typically below 2°), large moiré supercells…(Fig. 5c)". However, Fig. 5c is 3°, not less than 2°. Supporting evidence of a value less than or equal to 2° is required.*

Response:

We thank the reviewer for pointing out this inconsistency. We agree that the original Fig. 5c, which illustrated a 3° configuration, did not directly support our statement concerning the small-angle regime below 2°.

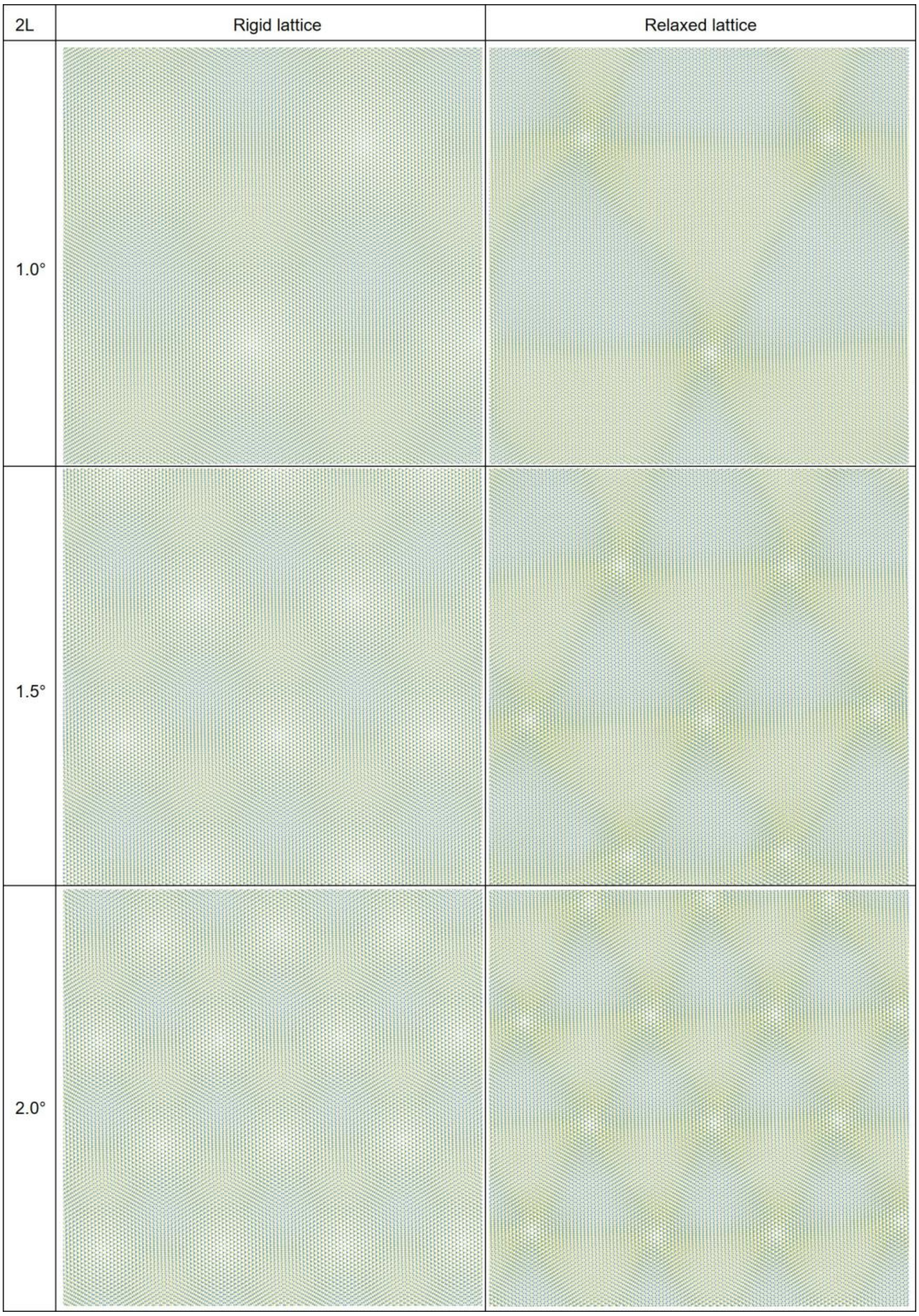


**Fig. R11 Moiré supercells of twisted bilayer $WS_2$ at small twist angles (θ = 1.0°, 1.5°, and 2.0°).** The moiré supercells are obtained using a newly constructed large-scale supercell containing 110,286 atoms, significantly extending the system size compared with the original model, in order to capture fully developed moiré reconstruction at small twist angles. The left and right columns show the rigid-lattice and relaxed-lattice atomistic configurations, respectively.

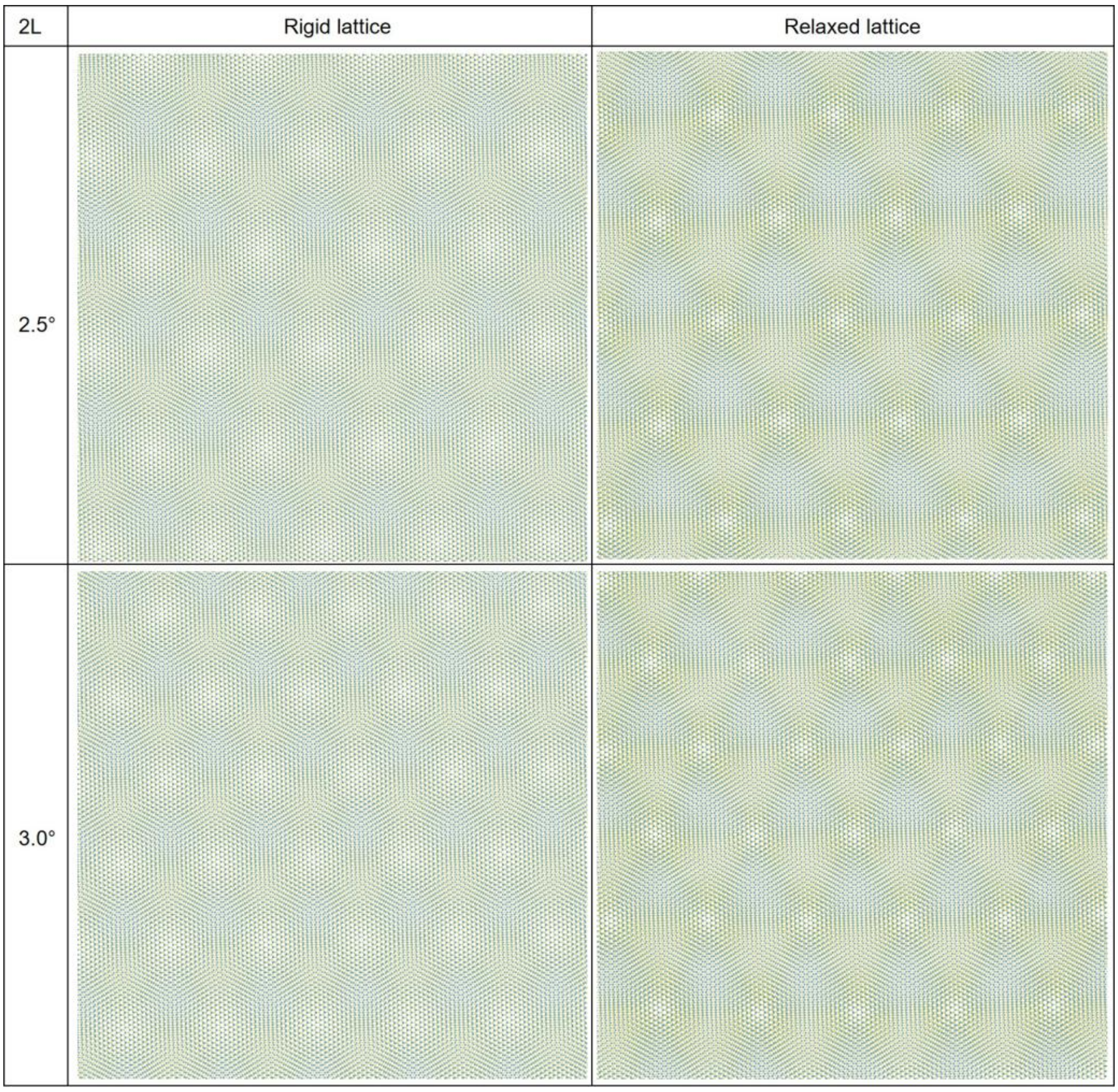


**Fig. R12 Moiré supercells of twisted bilayer $WS_2$ at intermediate twist angles (θ = 2.5° and 3.0°).**

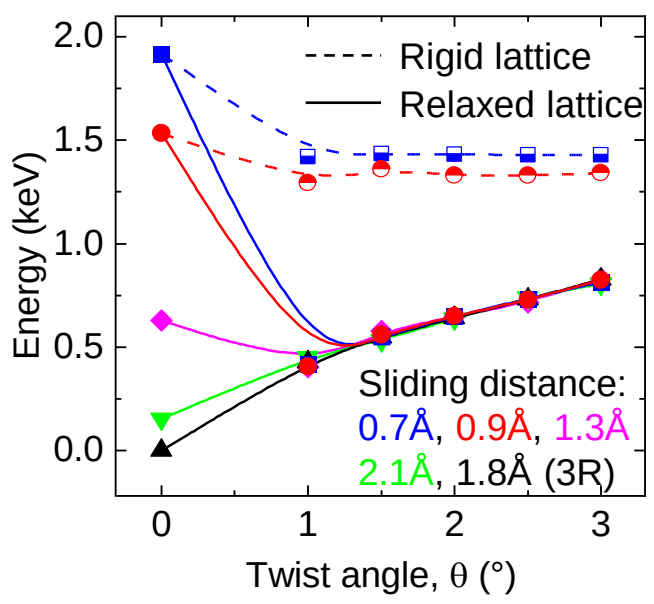


**Fig. R13 Twist-angle-dependent energy landscape of slipped bilayer $WS_2$, calculated using the enlarged supercell.** 0.7 Å, 0.9 Å, 0.7 Å, 0.9 Å, 1.3 Å, 1.8 Å, and 2.1 Å denote different in-plane sliding distances, where 1.8 Å corresponds to ideal 3R stacking. Symbols denote discrete calculated energies, and curves are B-spline guides to the eye.

To address this point, we expanded the atomistic supercell from approximately 7,500 atoms to 110,286 atoms, with lateral dimensions of approximately 490 Å × 490 Å, enabling calculations at smaller twist angles. We now provide rigid-lattice and fully relaxed bilayer $WS_2$ configurations at twist angles from 1.0° to 3.0° (Fig. R11-R12). These calculations reveal

pronounced atomic reconstruction in the relaxed structures within small-angle regime below 2°. The corresponding energy calculations show that interlayer twist can lower the energy of the representative slipped configurations (Fig. R13), consistent with the trend obtained in the original calculations.

The main text has been revised based on the above discussion, and the corresponding results have been added to Fig. 6b-c in the main text, the Methods section, and Figs. S13-S14 in the Supporting Information.

**Q8:** *The authors need to provide the goodness-of-fit values for Fig. 4b, especially for the Spiral data, showing the difference between the goodness-of-fit values for two-segment and full-segment fittings, and providing the confidence bands for the fit. This is because fitting each partition at around 1 Gpa is too subjective, and the data points are also suitable for full-segment fitting without partitioning.*

Response:

We thank the reviewer for this important comment. We agree that the original division of the Raman data at approximately 1 GPa required a more rigorous statistical assessment. We have therefore added full-range linear fits and two-segment fits for the $E_{2g}$ mode (Fig. R14), together with the corresponding R-square values, all of which are 0.99 or higher, and 95% confidence intervals for the fitted parameters.

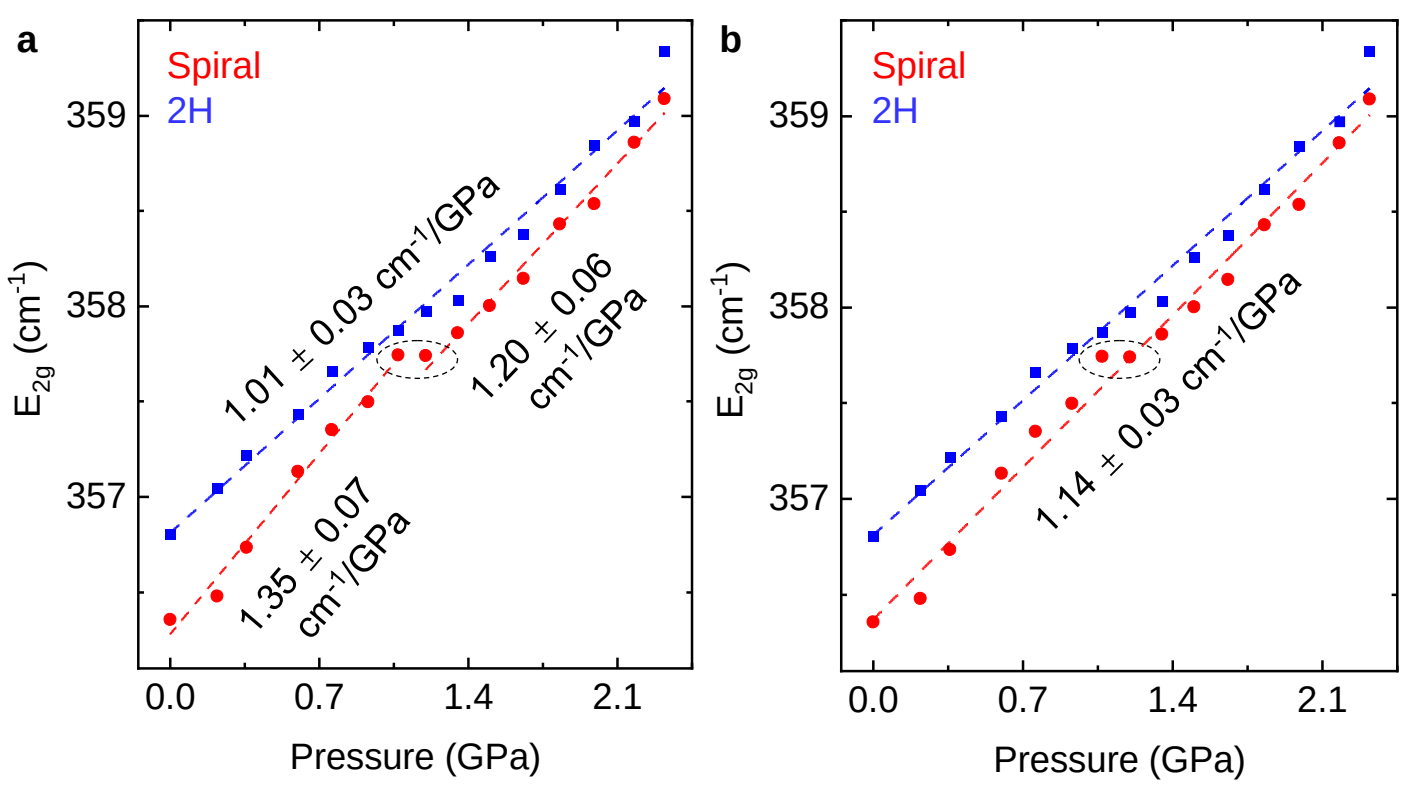


**Fig. R14 Goodness-of-fit comparison between two-segment and full-range linear fitting of the pressure-dependent Raman modes.** Pressure-dependent $E_{2g}$ frequencies of spiral and 2H $WS_2$ fitted using two-segment linear regressions with a division at 1 GPa (**a**) and full-range linear regressions over the entire measured pressure range (**b**). The pressure-dependent Raman results are the same data presented in main-text Fig. 4b. Symbols denote experimental data, and dashed lines denote linear fits. Black dashed circles highlight the local redshift of the spiral $E_{2g}$ mode near 1 GPa relative to the overall pressure-induced hardening trend. The fitted slopes are labelled together with their 95% confidence intervals. All fits yield R-square values of 0.99 or higher.

For the high-frequency Raman modes, the full-range linear fits provide better statistical goodness-of-fit than the two-segment fits (Fig. R14). We therefore do not interpret the two-segment fitting of the $E_{2g}$ data alone as statistical proof of a sharp transition at 1 GPa. Rather, the two-segment representation is included to visualize the local deviation from the overall

pressure-induced blueshift near 1 GPa, where the spiral $E_{2g}$ mode exhibits a transient redshift relative to the adjacent pressure points (black dashed circles in Fig. R14).

Importantly, the division near 1 GPa was not selected by optimizing the high-frequency Raman fits. This pressure range was independently identified by the saturation of the pressure-induced SHG pattern rotation (main-text Fig. 3f) and by a substantially more pronounced crossover in the shear-like ultralow-frequency Raman mode (Fig. R19 and main-text Fig. 5c), which is more directly sensitive to interlayer coupling. The coincidence of these independent observations supports the physical relevance of a crossover near 1 GPa, while the high-frequency Raman response provides a complementary, rather than standalone, signature.

**Q9:** *In line 197-199, the authors claimed, "Above 1 GPa, the interlayer twist saturates and the reconstruction no longer evolves, so the $E_{2g}$ shift mainly reflects conventional hydrostatic response (inset of Fig. 4b)…". However, exceeding 1 GPa, if only the traditional hydrostatic pressure response is considered, the $E_{2g}$ response slopes of Spira and 2H should be the same or similar, not still ~20% larger. Please connect this to the artificial fitting problem I raised above (No. 8).*

Response:

We thank the reviewer for this important comment. We agree that our original statement was overly simplified. In particular, saturation of the SHG-derived interlayer rotation near 1 GPa does not imply that all reconstruction-related lattice responses vanish or that spiral $WS_2$ should thereafter exhibit an $E_{2g}$ pressure coefficient identical to that of regular 2H $WS_2$.

As requested in Comment 8, we have re-evaluated the Raman fitting using both full-range and two-segment models (Fig. R14). Because the full-range fits provide better goodness-of-fit, we no longer use the two-segment $E_{2g}$ fitting as standalone statistical evidence for a distinct high-pressure regime. We have therefore removed the original statement that, above 1 GPa, the $E_{2g}$ shift mainly reflects a conventional hydrostatic response.

The $E_{2g}$ mode probes intralayer in-plane W-S vibrations and is therefore sensitive not only to pressure-induced lattice compression but also to the local in-plane strain landscape. Even after the average interlayer rotation no longer increases appreciably, spiral $WS_2$ retains screw-dislocation-induced strain, translation-disorder-induced strain heterogeneity, and local reconstruction-related strain associated with its non-3R stacking structure. Hydrostatic compression thus acts on a structurally distinct strain landscape, which can maintain an $E_{2g}$ response different from that of regular 2H $WS_2$. Accordingly, the high-pressure (> 1GPa) $E_{2g}$ behavior should not be interpreted as a return to an ideal 2H-like hydrostatic response.

Consistent with this interpretation, the pressure-dependent ultralow-frequency Raman data (Fig. R19) provide an independent view of the interfacial response. The shear-like interlayer mode of spiral $WS_2$ exhibits a pronounced crossover near 1 GPa, with its pressure coefficient remaining markedly different from that of the 2H reference above this pressure. This crossover occurs in the same pressure range as the saturation of the SHG pattern rotation (main-text Fig. 3f). Because the shear-like mode is sensitive to effective interlayer shear restoring forces that depend on local stacking registry, the persistence of a non-2H-like shear response indicates that the spiral stack remains dynamically distinct from regular 2H $WS_2$ at high pressure. It is

therefore not unexpected that the $E_{2g}$ mode also retains a pressure response different from that of the 2H reference.

We have revised the manuscript accordingly.

**Q10:** *In line 221-223, the author explains the principle of Cauchy linear elasticity. However, references should be included. Additionally, the author could add σij and εkl into Fig. 1a to improve the readability of the formula. Also, in line 223-229, the description is too theoretical. It would be better to add formulas and corresponding diagrams to SI to make it easier for readers to understand.*

Response:

We thank the reviewer for this valuable suggestion. We agree that the original discussion of Cauchy and micropolar elasticity was overly condensed and did not sufficiently define the relevant variables. We have therefore added references to the foundational literature on micropolar elasticity, together with a schematic comparison between the Cauchy and micropolar descriptions (Fig. R15).

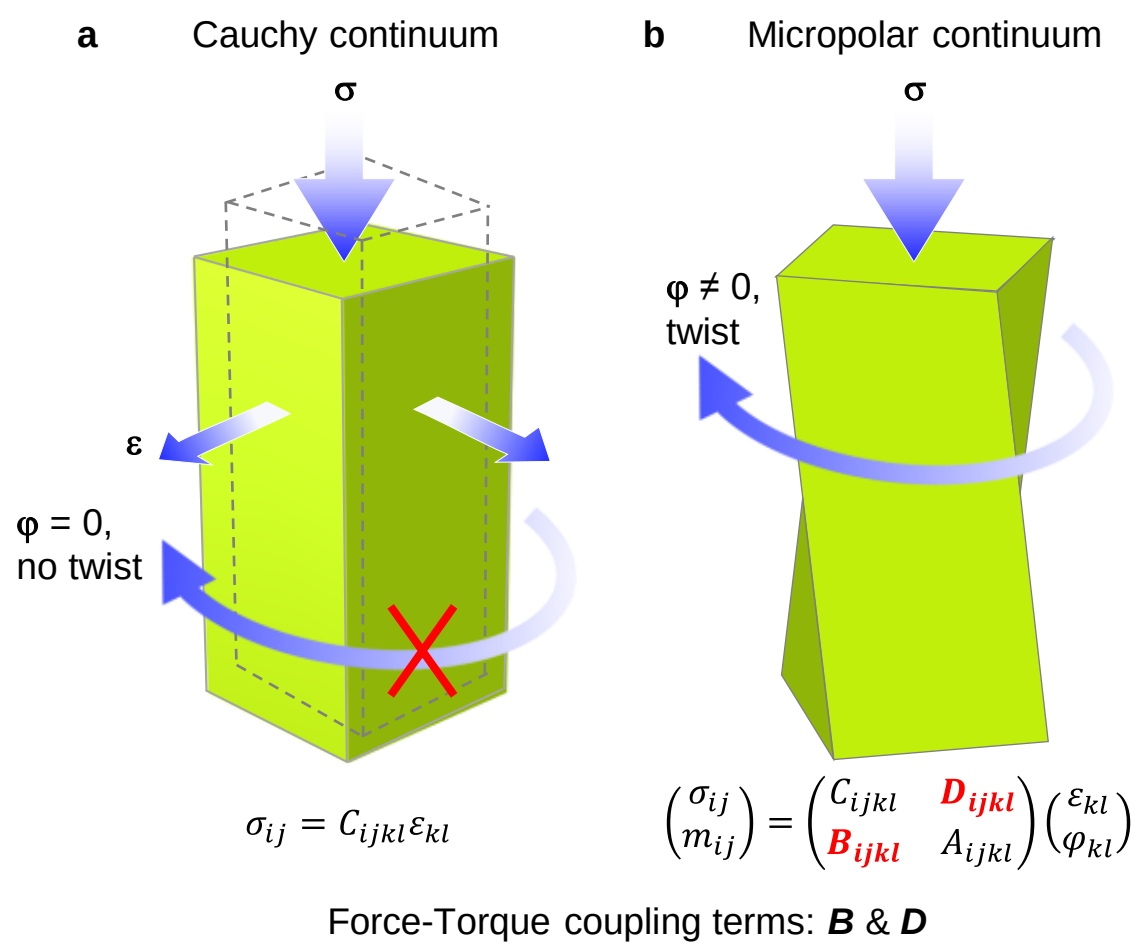


**Fig. R15 Schematic comparison between Cauchy and micropolar descriptions of compression-twist coupling. a**, under uniaxial stress (σ), a homogeneous Cauchy continuum develops conventional strain components (ε) but has no independent microrotation, φ= 0. **b**, a micropolar continuum additionally admits an internal microrotation φ. The cross-coupling tensors $B_{ijkl}$ and $D_{ijkl}$ enable coupling between axial deformation and rotational response[2].

In a homogeneous Cauchy linear elasticity, the force-stress tensor $\sigma_{ij}$ is related to the symmetric strain tensor $\varepsilon_{kl}$ through $\sigma_{ij} = C_{ijkl}\, \varepsilon_{kl}$. The rank-four elasticity tensor $C_{ijkl}$ is invariant under space inversion and therefore does not provide an intrinsic axial force-to-torque constitutive coupling[2]. Accordingly, a homogeneous Cauchy effective medium cannot exhibit a macroscopic twist response under pure axial loading (Fig. R15a).

A micropolar continuum extends this description by introducing an independent microrotation field $\theta_k$ and its associated curvature tensor $\varphi_{kl}= \partial\theta_k/\partial x_l$, together with the couple-stress tensor $m_{ij}$ [4]. The generalized constitutive relation can be written as $\begin{pmatrix}\sigma_{ij}\\ m_{ij}\end{pmatrix} = \begin{pmatrix}C_{ijkl} & D_{ijkl}\\ B_{ijkl} & A_{ijkl}\end{pmatrix}\begin{pmatrix}\varepsilon_{kl}\\ \varphi_{kl}\end{pmatrix}$

[2-4]. Here, $C_{ijkl}$ describe the conventional force-strain, whereas $A_{ijkl}$ describes the couple-stress response to microrotation gradients. The off-diagonal tensors $B_{ijkl}$ and $D_{ijkl}$ describe force-rotation coupling (Fig. R15b): strain-like deformation can generate couple stress through $B_{ijkl}$, whereas rotation gradients can contribute to force stress through $D_{ijkl}$. Such cross-coupling terms are symmetry-allowed in micropolar media and provide the continuum-mechanics basis for compression-twist conversion.

For spiral $WS_2$, we use this micropolar description as a conceptual effective-medium analogy rather than as a quantitative constitutive fit. The translation-disordered interfaces provide an internal structural coordinate, namely the local interlayer registry and rotation, that is absent from a homogeneous Cauchy description. Under compression, the reduction in interlayer spacing modifies the moiré-modulated stacking-energy landscape and can generate an interlayer rotational torque through atomic reconstruction. In this sense, the pressure-sensitive moiré interface acts as an atomic-scale analogue of a force-rotation-coupled micropolar layer. This framework connects the central observation of this work, namely pressure-driven interlayer twist in spiral $WS_2$, with the broader concept of compression-twist coupling in micropolar continuum mechanical systems.

**Q11:** *Why not use the parallel registry parameters measured by the STEM for moiré-based modeling (Fig. 5)? Furthermore, a triangular $WS_2$ bilayer structure is too simplistic compared to the complexity of a spiral structure. It should be expanded to at least three layers.*

Response:

We thank the reviewer for this important suggestion. We agree that the original bilayer model did not explicitly incorporate the translation disorder measured by STEM and did not capture the coupling between multiple interfaces in the multilayer spiral structure.

To address the first concern, we incorporated representative armchair-projected interlayer translations obtained from the cross-sectional STEM analysis into the bilayer model. We also expanded the atomistic bilayer model from the original approximately 7,500 atoms to 110,286 atoms in a supercell of approximately 490 Å × 490 Å, in order to resolve the small-angle moiré reconstruction more accurately. Specifically, the calculations considered lateral sliding distances of 0.7, 0.9, 1.3, and 2.1 Å along the armchair direction, while the 1.8 Å translation of ideal 3R stacking was included as a reference state, as shown in Fig. R11-R13. Because cross-sectional STEM resolves only the armchair-projected translation, these values are used as STEM-constrained initial registries rather than unique two-dimensional interface configurations. The updated bilayer calculations show that ideal 3R stacking retains its minimum energy at $\theta = 0°$ (Fig. R13). In contrast, several STEM-constrained slipped configurations lower their energy upon finite rotation and develop minima at small twist angles (Fig. R13). These results demonstrate that the translation disorder observed experimentally can reshape the rotational energy landscape and make finite interlayer rotation energetically accessible.

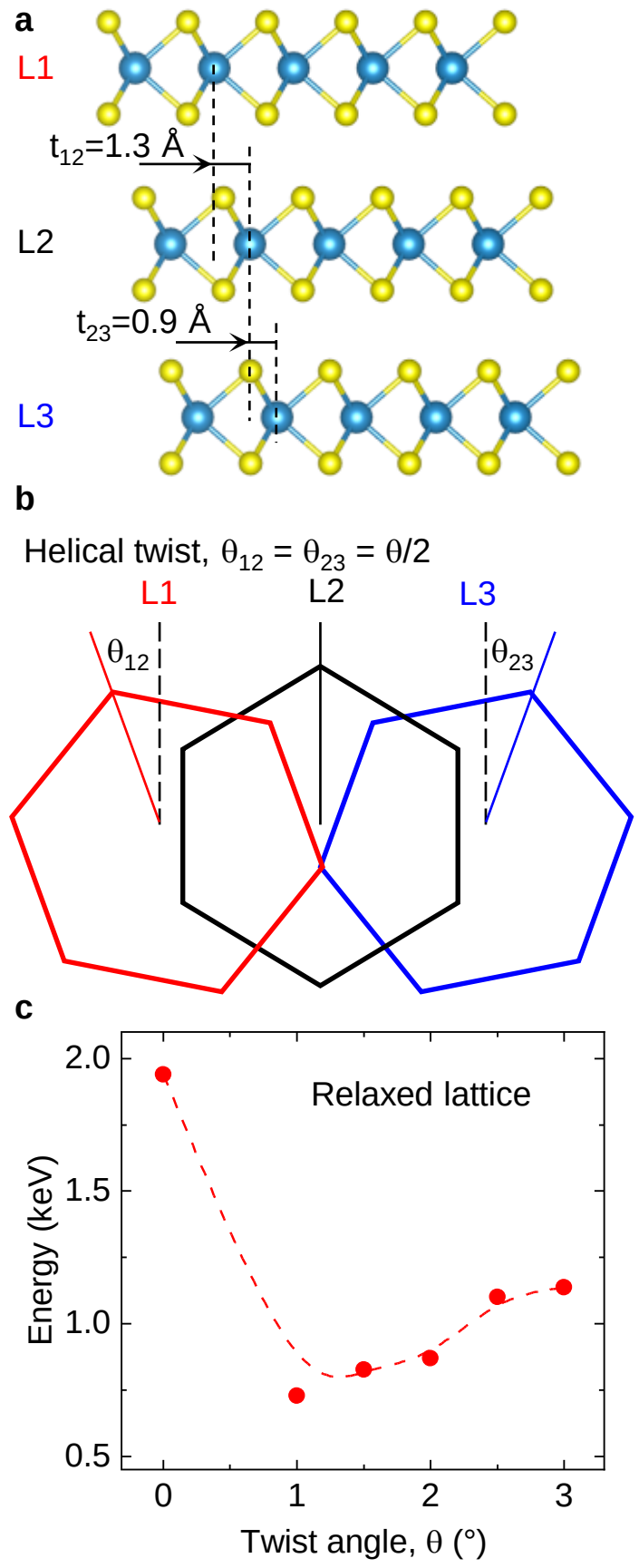


**Fig. R16 STEM-constrained helical trilayer model of spiral $WS_2$. a**, Side-view structural model of three parallel-oriented $WS_2$ layers. The armchair-projected translations between $L_1/L_2$ and $L_2/L_3$ are set to $t_{12}$ = 1.3 Å and $t_{23}$ = 0.9 Å, respectively, based on representative shifts resolved by cross-sectional STEM. Blue and yellow spheres denote W and S atoms, respectively. **b**, Definition of the helical twist configuration. $L_1$, $L_2$, and $L_3$ are rotated by -θ, 0, and +θ, respectively, giving $\theta_{12} = \theta_{23} = \theta/2$. **c**, Total energy of the STEM-constrained trilayer model as a function of twist angle θ.

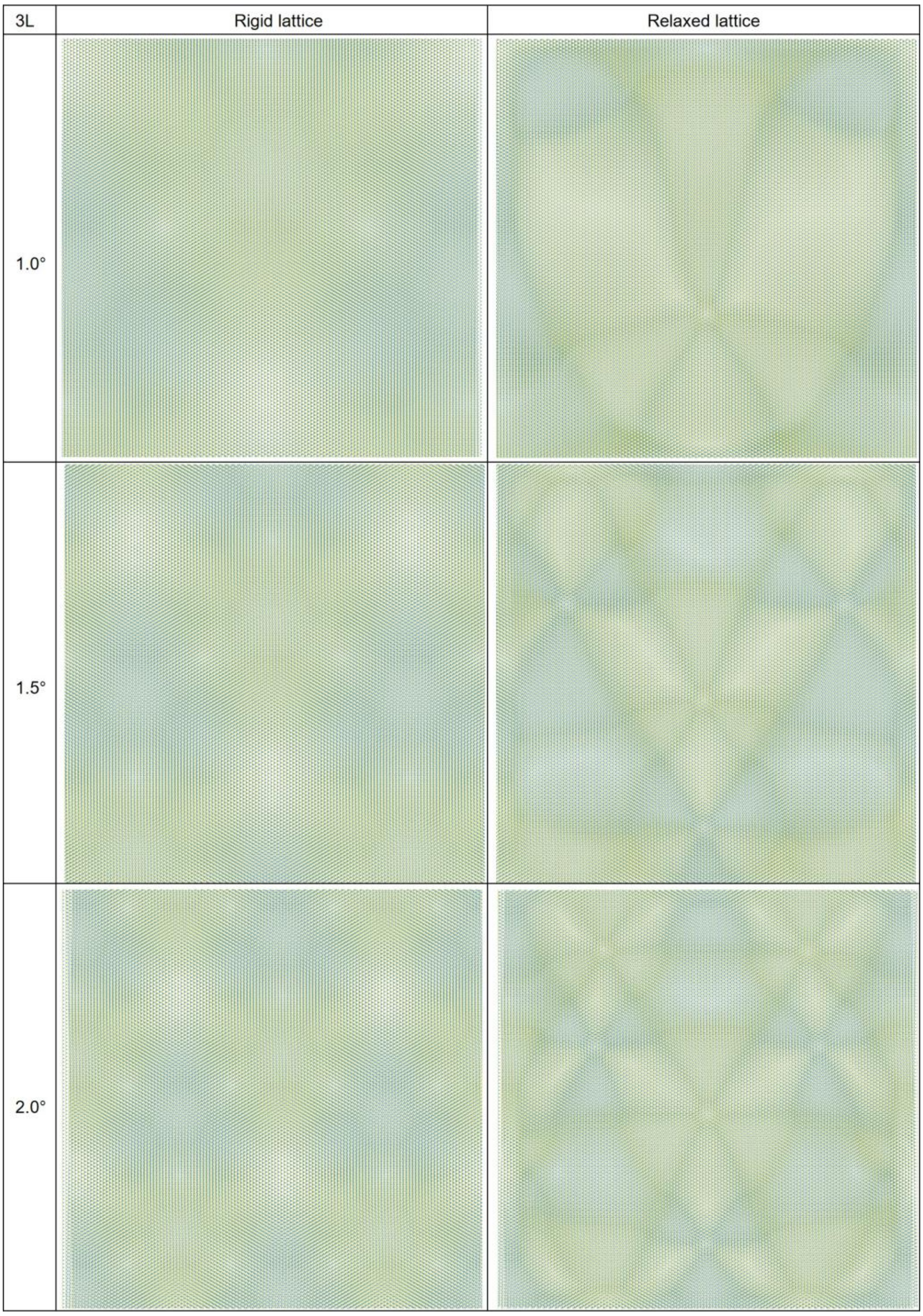


**Fig. R17 Moiré supercells of twisted trilayer $WS_2$ at small twist angles (θ = 1.0°, 1.5°, and 2.0°).** The moiré supercells contain160,065 atoms.

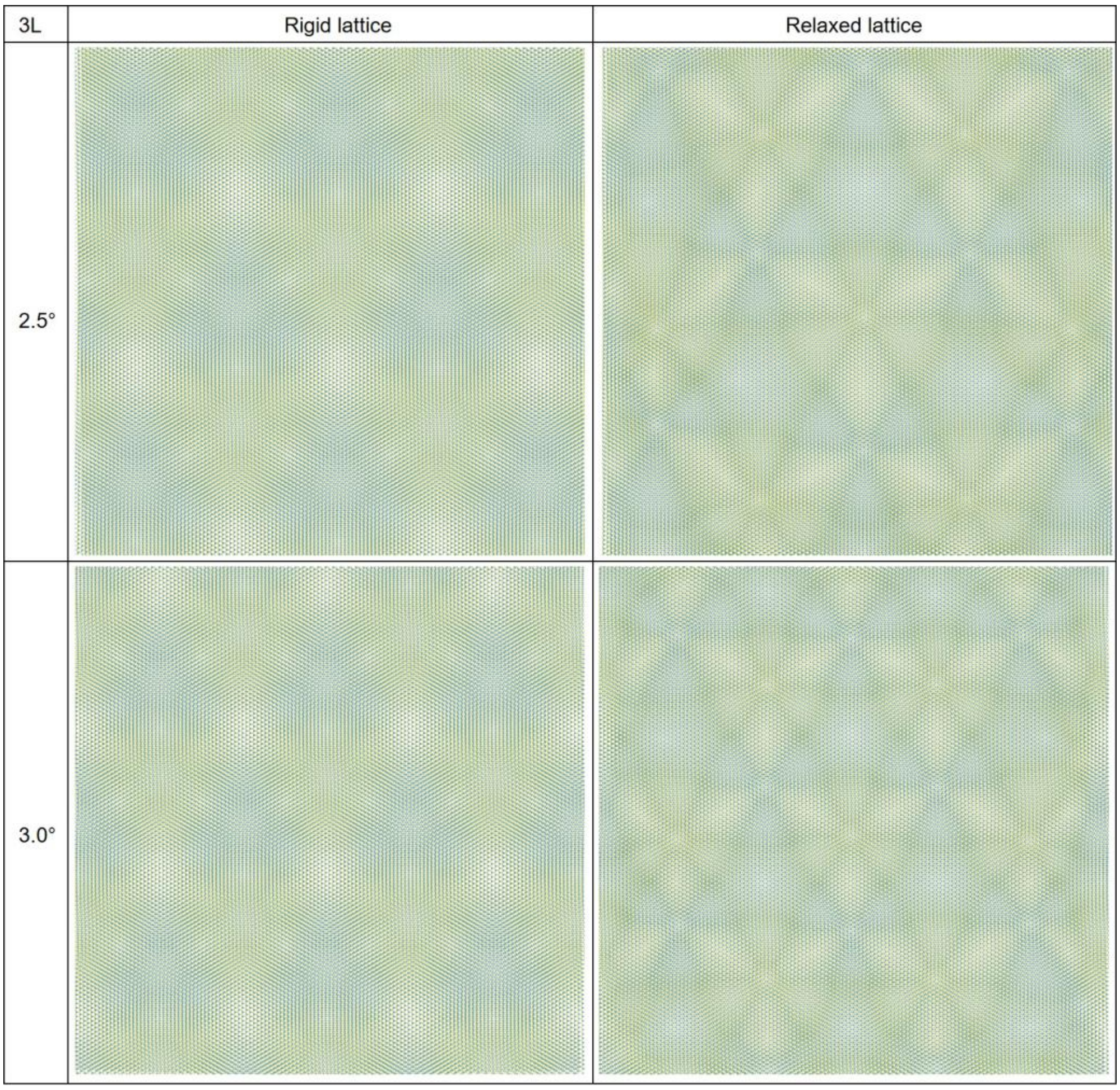


**Fig. R18 Moiré supercells of twisted trilayer $WS_2$ at small twist angles (θ = 2.5° and 3.0°).**

To address the second concern, we further constructed a trilayer $WS_2$ model containing 160,065 atoms in a supercell of approximately 490 Å × 490 Å (Fig. R16-R18). The successive interlayer translations were set to $t_{12}$ = 1.3 Å and $t_{23}$ = 0.9 Å (Fig. R16a), corresponding to representative projected shifts measured by STEM. A helical twist configuration was imposed with $\theta_{12} = \theta_{23} = \theta/2$, corresponding to layer orientations of -θ/2, 0, and +θ/2 for $L_1$, $L_2$, and $L_3$, respectively (Fig. R16b). This trilayer model contains two coupled moiré interfaces and allows the middle layer to respond simultaneously to the registry-dependent interactions from both neighbouring layers. The rigid and relaxed trilayer configurations reveal atomic reconstruction over the range θ = 1.0° to 3.0° (Fig. R17-R18). More importantly, the relaxed trilayer energy decreases substantially from θ = 0° and reaches a minimum near small non-zero twist angle, followed by an increase at larger twist angles (Fig. R16c). Thus, finite-angle stabilization persists in a minimal multilayer model containing two coupled interfaces and is consistent with the energy-lowering trend obtained for the STEM-constrained slipped bilayers.

We emphasize that the trilayer model is not intended to reproduce the exact registry distribution or layer-resolved twist profile of the full multilayer spiral crystal. Rather, it establishes that the reconstruction-mediated rotational energy landscape remains operative beyond a single bilayer interface, supporting the extension of the proposed compression-twist mechanism to the multilayer spiral structure.

The main text has been revised based on the above discussion, and the corresponding results have been added to Figs. S16-S18 in the Supporting Information.

**Minor issues:**

**Q1:** *The discission on the behavior and applications of 3D multilayer moiré superstructures (lines 45-49) lacks an aspect and citations regarding the conductive properties of spiral TMDs materials.*

Response:

We thank the reviewer for pointing out this missing aspect.

The study on vertically conductive $MoS_2$ spiral pyramids showed that a threading screw dislocation can provide a helical current pathway along the vertical direction[15]. This leads to enhanced cross-plane conductance compared with conventional layered TMDs, where interlayer transport is usually limited by weak van der Waals coupling[15]. The result highlights that screw-dislocation-driven spiral architectures are not only structurally distinct multilayer systems but can also introduce unconventional electrical transport channels.

We have therefore revised the Introduction to include this aspect. The relevant sentence now reads: "Extending to three-dimensional multilayer stacks ('3D twistronics') introduces hierarchical interactions among incommensurate moiré-of-moiré superstructures, which can accommodate additional flat bands and manifest exotic behaviors, including topological phase transitions, novel resonant states, the opto-twistronic Hall effect, and screw-dislocation-enabled helical current pathways."

**Q2:** *The authors should clearly indicate which figure and section in SI corresponds to "(Supporting Information)" in the whole manuscript. Please check the entire manuscript against this information and add specific details in parentheses if only SI is mentioned.*

Response:

We thank the reviewer for this helpful suggestion. We have carefully checked the entire manuscript and replaced generic "Supporting Information" references with specific section, note, figure, or table numbers wherever applicable.

**Q3:** *The current image resolution is not high enough and needs to be improved. Also, the overall aesthetics of the graphics need to be enhanced. For example, the broken axis in Fig. 4a is too tilted.*

Response:

We thank the reviewer for this helpful comment. We have improved the resolution and graphical quality of all main-text and supporting figures. We have also revised the broken axis in Fig. 4a to make it less tilted and visually cleaner.

**Q4:** *In line 209-211, the authors claimed, "Previous studies…(Fig. 5c)…". However, this part of the article mainly describes the Raman data in Fig. 4, so Fig. 5c should be the result of the research in the following. The author needs to improve the logical consistency of the writing.*

Response:

We thank the reviewer for pointing out this logical inconsistency.

We have revised this sentence to separate the literature evidence from our own calculation. Specifically, we now cite the previous work on twist-induced reconstruction and $E_{2g}$ splitting in reconstructed TMD moiré superlattices[11], and then state that our atomistic calculations show a consistent reconstruction tendency. We have also updated the figure reference: the revised main-text Fig. 6c now shows θ = 1.0° and 1.5° configurations, as suggested in the reviewer#2's comment 7, while the θ = 2.0° to 3.0° results are provided in the Supplementary Fig. 14.

We have therefore revised the main text to include this aspect. The relevant sentence now reads: "Previous studies have shown that twist angles of 2° to 6°increase the relative area of strain solitons, consistent with our calculation results in Supplementary Fig. 14, and can hence break the in-plane threefold rotational symmetry, splitting the doubly degenerate $E_{2g}$ mode[11]."

**Q5:** *In line 209-211, the authors claimed, "…a periodic moiré pattern with alternating high-energy (red) and low-energy (blue) domains…". However, in Fig. 5a, the low-energy domains should be described as purple, which is not blue. Also, Fig. 5a and Fig. S7b are the same. Please delete the redundant data.*

Response:

We thank the reviewer for pointing this out. We have corrected the color description from "blue" to "purple" so that it matches the low-energy domains shown in Fig. 6a. We have also checked the Supporting Information and removed the redundant Supplementary Fig. 7b.

**Q6:** *In SI, the authors need to supplement the type of scanning probe model and basic data in the section of AFM characterization.*

Response:

We thank the reviewer for this helpful suggestion. In the Methods section of Supporting Information, we had stated that the AFM measurements were performed using a Vistascope AFM system operated in tapping mode.

**Point-by-point response to Reviewer #3's comments:**

**Overall Comment:** *The authors applied longitudinal stress to the spiral $WS_2$ using DAC. Through polarization-dependent SHG and Raman spectroscopy, they observed reversible tuning of interlayer twist up to an average of 0.2° per layer at a pressure of approximately 1 GPa. However, I cannot recommend its publication in NC due to the following reasons, or a major revision is seriously needed for any further consideration.*

Response:

We thank the reviewer for the constructive assessment and insightful comments. In the revised manuscript, we have substantially strengthened the work by adding ultralow-frequency Raman measurements, high-pressure SHG control experiments, and expanded atomistic calculations. These revisions address the reviewer's major concerns and improve the physical foundation of the manuscript.

**Q1:** *There is no evidence from low-frequency Raman spectroscopy (shear modes and breathing modes), and the small changes in high-frequency Raman are insufficient to indicate variations in interlayer coupling upon interlayer rotation and compression.*

Response:

We thank the reviewer for this important comment. We agree that the high-frequency Raman results alone provide only indirect evidence for pressure-dependent interlayer structural evolution. We have therefore added ultralow-frequency Raman measurements that directly probe the interlayer shear response of spiral $WS_2$ and the 2H reference under pressure (Fig. R19).

The ultralow-frequency Raman spectra were measured in a backscattering configuration at 300 K using 442 nm excitation from a HeCd laser, corresponding to an excitation energy of 2.81 eV. This excitation wavelength was selected because it lies near the C-exciton resonance of $WS_2$, where resonance enhancement can increase the Raman cross section of otherwise weak interlayer phonon processes[16-18]. Plasma lines were removed using BragGrate bandpass filters, and three BragGrate notch filters enabled measurements down to ultralow wavenumber. The raw spectrum at ambient pressure, the fitted baseline, the baseline-subtracted spectrum, and the Lorentzian fitting procedure are now explicitly shown in Fig. R9a-b. The same baseline-subtraction and fitting procedure was applied consistently to the pressure-dependent spectra.

Under the present measurement conditions, we did not resolve a reproducible and spectrally distinct layer-breathing feature in the bulk-like spiral $WS_2$. This may partly reflect the softening of the lowest acoustic-like breathing branch with increasing layer number, while the Raman visibility of higher breathing branches can be limited by stacking-dependent selection rules and spectral overlap. Such a lack of a resolved layer-breathing feature is also consistent with previous low-frequency Raman studies of bulk 2H- and 3R-stacked TMDs[17-19]. We therefore do not use the non-observation of a resolved layer-breathing peak as evidence for the proposed mechanism. Instead, we focus on the shear-like response, which directly probes the effective lateral restoring force and local stacking registry.

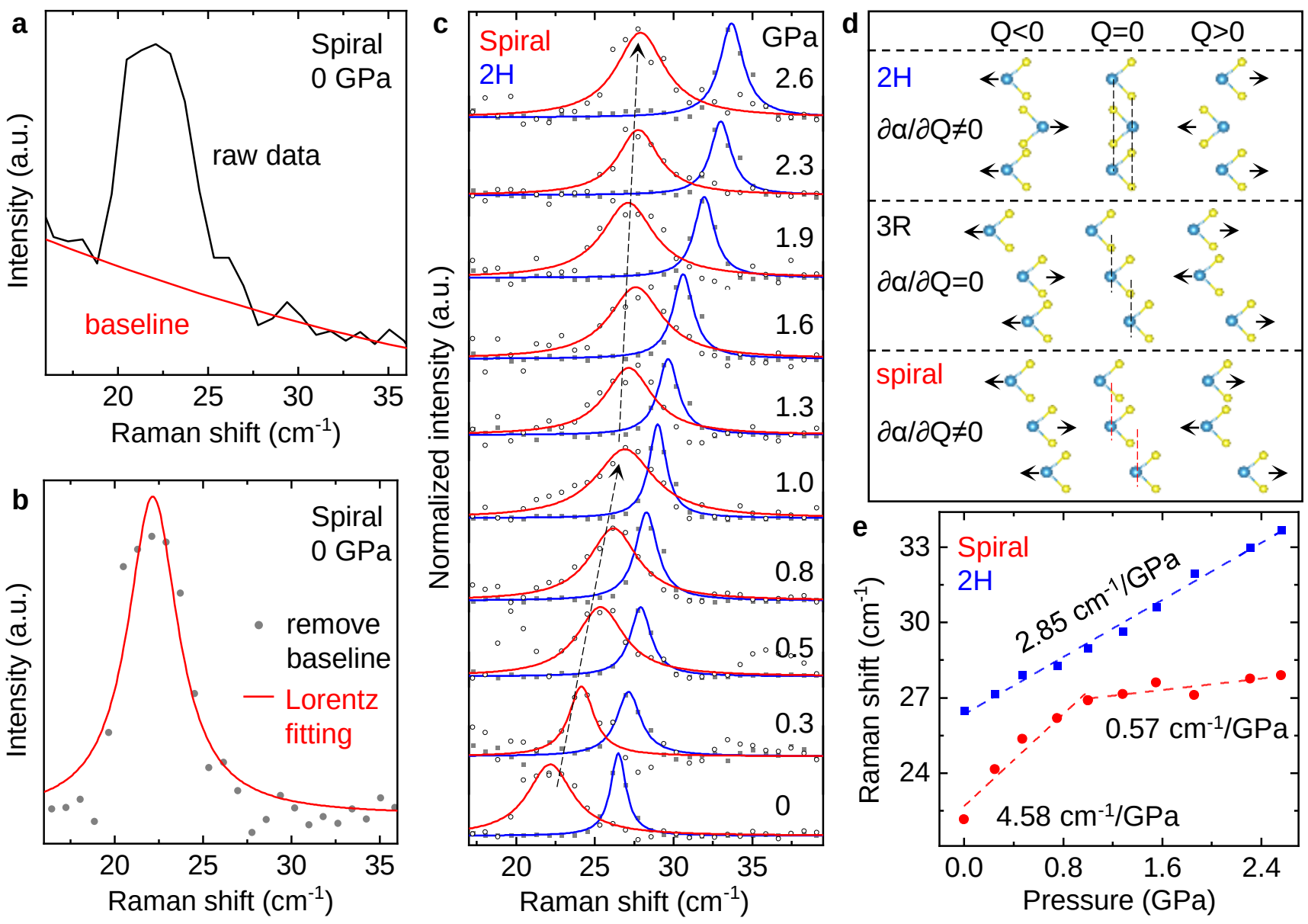


**Fig. R19 Ultralow-frequency Raman signatures of pressure-dependent interlayer shear coupling in spiral $WS_2$. a-b**, Raw ambient-pressure ultralow-frequency Raman spectrum of spiral $WS_2$, the fitted spectral baseline, and the baseline-subtracted spectrum fitted with a Lorentzian line shape. **c**, Pressure-dependent ultralow-frequency Raman spectra of spiral $WS_2$ (red) and 2H $WS_2$ (blue) recorded with 442 nm excitation. Symbols and solid curves denote the experimental spectra and fitted profiles, respectively. The dashed lines trace the pressure evolution of the shear-like features. **d**, Schematic Raman-tensor response of the high-frequency shear branch under the normal coordinate Q. Random interlayer translations in spiral $WS_2$ break the interfacial equivalence of ideal 3R stacking, yielding a finite polarizability derivative, $\partial\alpha/\partial Q \neq 0$, and hence a Raman-active shear-like response. **e**, Pressure dependence of the fitted shear-mode frequencies.

At ambient pressure, spiral $WS_2$ displays a weak and broad shear-like feature at 22.2 $cm^{-1}$, redshifted relative to the high-frequency shear mode of the 2H reference at 26.5 $cm^{-1}$ (Fig. R19c and R19e). We conservatively assign this feature to a disorder-activated high-frequency shear-like interlayer mode. In ideal 3R stacking, the equivalent interfaces yield nearly identical bond-polarizability derivatives, so that the interfacial contributions to the Raman tensor, $R \propto \partial\alpha/\partial Q$, cancel for the high-frequency shear branch (Fig. R19d). Here, α is the electronic polarizability tensor and Q is the normal coordinate. The opposite branch selectivity occurs in 2H stacking (Fig. R19d), where the alternating interface orientation permits coherent addition of the high-frequency shear mode contributions[17-19]. Spiral $WS_2$ lies between these limits: its layers remain parallel, but layer-to-layer translations fluctuate away from the regular 3R sequence (Fig. R19d), rendering the interfacial bond-polarizability derivatives inequivalent. The incomplete cancellation therefore leaves a finite Raman response for the high-frequency branch. Its detection is further facilitated by the 442 nm excitation, which lies near the C-exciton resonance of $WS_2$ and enhances the exciton-mediated Raman scattering amplitude[16,20]. Within the linear chain model, the bulk-like high-frequency shear branch obeys $\omega^2 = \alpha_{/\!/}/(\pi^2 c^2 \mu)$, where $\alpha_{/\!/}$ is the interlayer shear force constant and μ is the monolayer areal mass density[20,21]. Treating the spiral feature as an effective high-frequency shear-like mode gives $\alpha_{/\!/,\mathrm{spiral,eff}} = 2.09 \times 10^{19}$ N/m$^3$,

indicating a 30% reduction relative to 2H $WS_2$ in the effective lateral restoring force. This reduction can be attributed to the expanded interlayer spacing, which reduces interlayer electronic overlap and weakens the lateral corrugation, as well as to random sliding and screw-dislocation-induced strain, which disrupt the equivalence of interlayer registries and reduce the long-range coherent restoring response[17,22]. Related stacking-dependent shear-mode softening and a reduced interfacial shear force constant have been reported for AB′-stacked $MoSe_2$ bilayers and weakly coupled twisted multilayer graphene interfaces, respectively[20,22]. These interfacial shear-like phonon signatures provide a dynamical consequence of the slipped, metastable stacking that enables twist activation in spiral $WS_2$.

The pressure evolution of this mode further links interlayer engineering to the twist degree of freedom. Below 1 GPa, the spiral feature hardens at 4.58 $cm^{-1}$/GPa, compared with 2.85 $cm^{-1}$/GPa for 2H (Fig. R17e). This enhancement coincides with the SHG pattern rotation and anomalous $E_{2g}$ hardening (main-text Fig. 3f and 4b), consistent with direct compression acting together with rotation-driven reconstruction to increase the effective lateral restoring force and hence the shear-mode blueshift[21]. Once the SHG rotation saturates near 1 GPa, this reconstruction-driven contribution is diminished, and the pressure coefficient of shear-like mode falls to 0.57 $cm^{-1}$/GPa (Fig. R17e). The markedly reduced post-crossover pressure coefficient does not indicate that every local interface ceases to stiffen. Instead, the observed broad shear-like feature is more appropriately viewed as a Raman-selected collective response; it combines unresolved contributions from reconstructed interfacial regions with different registry, local lateral stiffness, and Raman-tensor activity. Upon further compression, the reconstructed interfacial landscape may become locally more uniform, increasing the fraction of regions that exhibit near-3R-like cancellation of the high-frequency shear Raman tensor[19]. This enhanced cancellation could suppress the Raman weight of relatively stiff and higher-frequency components. The residual signal may then become increasingly weighted toward soliton-like, or domain-boundary regions[11], where the cancellation remains incomplete and the local lateral response is comparatively compliant. A pressure-dependent redistribution among these unresolved components would partially limit the blueshift of the fitted peak centroid, even if local interlayer stiffness continues to increase throughout the multilayer. The Raman-selected shear-like feature can therefore harden much more slowly than the shear phonon in the laterally uniform 2H reference.

Taken together, the high- and ultralow-frequency Raman responses under pressure are consistent with compression-driven twist and interfacial reconstruction in the metastable spiral stacking, providing complementary vibrational support for the proposed twist degree-of-freedom mechanism.

We have added the above discussion to the "Ultralow-frequency shear signatures of compression-twist coupling" section in the main text and to Supplementary Fig. 19.

**Q2:** *The analysis of high-frequency Raman is insufficient; in particular, the pressure-dependent frequency difference between the spiral and 2H samples are not explained in detail.*

Response:

We thank the reviewer for this important comment. We agree that the original discussion did

not sufficiently explain the distinct pressure evolution of the high-frequency Raman modes in spiral and regular 2H $WS_2$. We have therefore expanded the analysis to explain the difference in the post-crossover $E_{2g}$ pressure coefficients and to clarify how the high-frequency Raman response complements the SHG and ultralow-frequency Raman results.

In the main text, we have expanded the high-frequency Raman analysis as follows. Below 1 GPa, the $E_{2g}$ mode of spiral $WS_2$ hardens about 35% faster than that of the 2H reference (main-text Fig. 4b), consistent with hydrostatic in-plane compression acting together with twist-driven moiré reconstruction. Above about 1 GPa, the SHG-derived rotation no longer increases appreciably (main-text Fig. 3f) and the $E_{2g}$ pressure coefficient decreases (main-text Fig. 4b), indicating a diminished reconstruction-related contribution. Meanwhile, the $A_{1g}$ slopes of spiral and 2H $WS_2$ remain similar (main-text Fig. 4c), arguing against extensive pressure-induced sliding toward a denser 3R registry. The absence of resolvable $E_{2g}$ splitting further confines the rotation to the small-angle regime (main-text Fig. 4a). Together, these observations support a small, reconstruction-mediated compression-twist response rather than large-scale interlayer translation.

Building on this analysis, and particularly in conjunction with the newly added ultralow-frequency Raman data (Fig. R19), we further analyze the distinct post-crossover $E_{2g}$ response (main-text Fig. 4b) and its physical relationship to the pressure-dependent interfacial shear response as follows:

To further address the post-crossover regime, we next consider why the $E_{2g}$ pressure coefficient of spiral $WS_2$ remains larger than that of the 2H reference even after the SHG-derived interlayer rotation no longer increases appreciably above 1 GPa (main-text Fig. 4b). The $E_{2g}$ mode probes intralayer in-plane W-S vibrations and is therefore sensitive not only to pressure-induced lattice compression but also to the local in-plane strain landscape. Above 1 GPa, saturation of SHG rotational response does not imply that the spiral stack becomes structurally equivalent to regular 2H $WS_2$. The underlying non-3R, translation-disordered stacking configuration associated with screw-dislocation-driven growth provides a local bonding and strain environment distinct from that of the 2H reference. Hydrostatic compression thus acts on a structurally distinct strain landscape, which can maintain an $E_{2g}$ response different from that of regular 2H $WS_2$.

Consistent with this interpretation, the pressure-dependent ultralow-frequency Raman data (Fig. R19) provide an independent probe of the interfacial response. The shear-like interlayer mode of spiral $WS_2$ exhibits a pronounced crossover near 1 GPa, with its pressure coefficient remaining markedly different from that of the 2H reference above this pressure. This crossover occurs in the same pressure range as the saturation of the SHG pattern rotation (main-text Fig. 3f). Because the shear-like mode is sensitive to effective interlayer shear restoring forces that depend on local stacking registry, the persistence of a non-2H-like shear response indicates that the spiral stack remains dynamically distinct from regular 2H $WS_2$ at high pressure. The shear-like mode and the $E_{2g}$ mode probe different components of lattice dynamics: the former is governed primarily by the registry-dependent lateral interlayer restoring force, whereas the latter reflects intralayer W-S bonding and in-plane strain. The ultralow-frequency result shows that the high-pressure spiral interface remains dynamically different from regular 2H $WS_2$,

making it physically reasonable that the $E_{2g}$ response also does not converge to that of the 2H reference.

**Q3:** *The preparation of the spiral $WS_2$ sample is not mentioned. In addition, this sample is not that representative in 2D-related studies.*

Response:

We thank the reviewer for this comment. We apologize that the synthesis procedure was not sufficiently visible in the original submission. We also agree that screw-dislocation-driven spiral $WS_2$ is not intended to represent conventional exfoliated or layer-by-layer-grown $WS_2$. Rather, it is a distinct class of multilayer van der Waals crystal deliberately selected here because its screw-dislocation-driven growth and translation-disordered interfaces provide an experimentally accessible platform for investigating an internal interlayer rotational degree of freedom.

The preparation of the spiral $WS_2$ samples is described in the Methods section of the Supporting Information. The relevant paragraph now reads: Spiral $WS_2$ nanostructures were synthesized using a previously reported water-vapor-assisted chemical vapor transport growth[23]. A custom-built reactor system consisted of a 1-inch outer diameter fused silica tube equipped with pressure and gas flow controls, a Lindberg Blue M three-zone tube furnace, and two heating tapes, was used as the reactor. 100 mg $WS_2$ precursor powder (Alfa Aesar) was placed in the first zone of the three-zone furnace, and the $SiO_2$/Si substrate was placed between the second and the third zone with the polished side facing up. 1 g $CaSO_4 \cdot 2H_2O$ powder (Sigma Aldrich) was placed upstream from the heating zone as the water vapor source and heated with two heating tapes. With 100 sccm argon flowing at 800 torr, the second zone was heated to 1200 °C at a rate of 20 °C/min, and simultaneously the third zone was heated to 700 °C at the same rate, while the $CaSO_4 \cdot 2H_2O$ was not heated. Once the furnace temperatures were reached, the $CaSO_4 \cdot 2H_2O$ was heated to around 90 °C to release the water vapor. After all the temperatures were stabilized, the $WS_2$ boat was pushed into the second zone by a magnet coupled positioner and a quartz rod to initiate the reaction. After 15 min reaction, the furnace was opened and cooled down rapidly.

Spiral layered crystals are scientifically relevant not as generic 2D materials, but as a structurally distinct route toward multilayer and three-dimensional twistronics. Structurally, screw-dislocation-driven growth can generate aligned or continuously twisted TMDs multilayers, as reported in our previous studies[5,7]. Optical studies have shown strong SHG and THG responses in spiral $WS_2$ arising from broken inversion symmetry, while subsequent work identified large SHG enhancement associated with excitonic resonance and interband Berry connection[24,25]. Supertwisted $WS_2$ spirals further exhibit moiré-magnified intrinsic heterostrain and broken rotational symmetry[26]. Most recently, a three-dimensional spiral $WS_2$ lattice was shown to exhibit an opto-twistronic Hall effect governed by structural chirality, moiré potential and coherence length[27]. A recent review has also summarized the broader opportunities of two-dimensional spirals in electrical, optoelectronic, thermal, mechanical and catalytic research[28].

Accordingly, the purpose of the present work is not to generalize compression-twist coupling to all 2D materials. Instead, it establishes spiral $WS_2$ as a specific screw-dislocation-engineered

vdW platform in which metastable interlayer registry can activate a pressure-tunable twist degree of freedom.

**Q4:** *There is no evidence provided for the existence of a moiré potential in the spiral sample.*

Response:

We thank the reviewer for raising this point. We clarify that this work does not claim direct experimental observation of an electronic moiré potential in spiral $WS_2$. The term "moiré potential" appears only in the Introduction as a general description of conventional twisted or lattice-mismatched van der Waals superlattices, where spatially varying registry can modulate electronic and excitonic energy landscapes.

In the present spiral $WS_2$ system, our conclusions do not rely on the existence or direct measurement of an electronic moiré potential. Instead, the relevant quantity is the registry-dependent interlayer stacking-energy landscape, which is evaluated in the atomistic calculations as a function of interlayer translation, twist angle, structural relaxation, and interlayer spacing. The calculated energy landscape provides the basis for the discussion of rotational torque and finite-angle stabilization.

**Q5:** *The separation arrangement of main text and figures in the manuscript makes it particularly uncomfortable for reviewers to read.*

Response:

We thank the reviewer for this helpful suggestion. We have reorganized the manuscript layout so that each main-text figure is placed close to its first relevant discussion.

**Q6:** *The authors should discuss the effect from the high-pressure medium (such as compressed diamond, pressure-transmitting medium, hBN, etc.) on the polarization-dependent SHG signal.*

Response:

We thank the reviewer for raising this important concern. We agree that possible polarization-dependent optical effects arising from the diamond anvil cell environment, including compressed diamond, silicone oil, and hBN encapsulation, must be excluded before attributing the pressure-dependent SHG pattern rotation to spiral $WS_2$.

The high-pressure measurements were performed in a diamond anvil cell composed of two diamond anvils with 800 μm culets and a 304 stainless-steel gasket of approximately 30 μm thickness. Silicone oil was used as the pressure-transmitting medium, and pressure was calibrated using the standard ruby-luminescence method. To assess the combined optical effects of the diamond anvils, pressure medium, hBN encapsulation, and polarization-resolved SHG measurement geometry, we performed control measurements on hBN-encapsulated 2H and 3R $MoS_2$ flakes (prepared by mechanical exfoliation) under the same DAC and optical conditions as those used for the spiral sample (Fig. R20a).

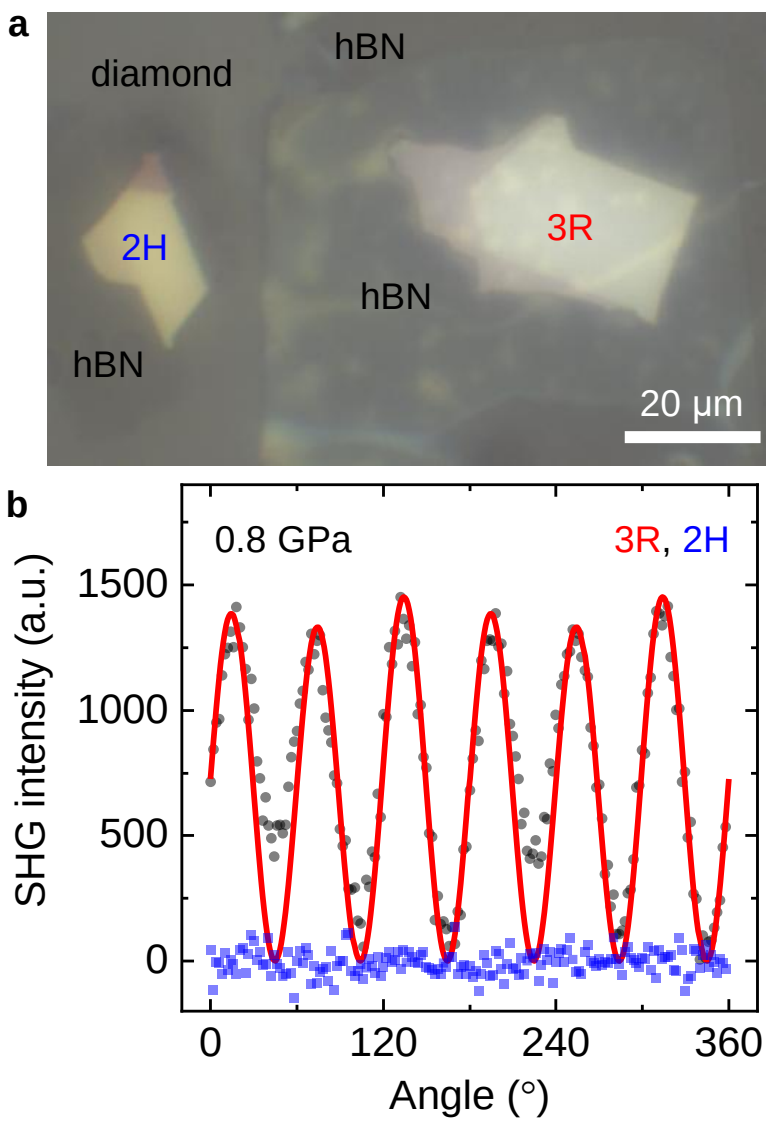


**Fig. R20 High-pressure SHG control of hBN-encapsulated 2H and 3R $MoS_2$. a**, optical image of the 2H and 3R $MoS_2$ regions encapsulated by hBN and loaded in the diamond-anvil cell at 0.8 GPa. **b**, polarization-resolved SHG intensities of the 3R and 2H regions measured under the same DAC and optical conditions as those used for spiral $WS_2$, including 800 nm excitation, hBN encapsulation, silicone-oil pressure medium, and diamond anvils. Grey circles and red curve denote the measured SHG intensity and fit for the 3R region, respectively. Blue squares denote the signal from the 2H region.

At 0.8 GPa, the 3R reference exhibits a pronounced sixfold SHG pattern, whereas the adjacent 2H region shows no detectable SHG signal above the background level (Fig. R20b). This comparison confirms that the hBN encapsulation, compressed silicone oil, and diamond optical path do not generate an artificial sixfold SHG response under the present measurement conditions.

More importantly, we measured the pressure-dependent polarization-resolved SHG response of the 3R reference from ambient pressure to 2.1 GPa (Fig. R21). Within experimental uncertainty, the combined high-pressure optical environment does not induce a systematic pressure-dependent rotation of the SHG pattern relative to the crystallographic edge reference. Further details are provided in our response to Reviewer#3's Comment 7 (Fig. R21).

These controls directly rule out, within the experimental uncertainty, the relevant artifact of a systematic rotation of the polarization-dependent SHG pattern caused by compressed diamond, silicone oil, or hBN encapsulation. The pressure-dependent relative rotation observed in spiral $WS_2$ is therefore attributed to an intrinsic structural response of the spiral stack rather than to the DAC optical environment.

The above discussion has been incorporated into the Methods section and Supplementary Fig. 21.

**Q7:** *The authors should also provide a comparative study of the polarization-dependent SHG study for 3R stacking under high pressure.*

We thank the reviewer for this important suggestion. We have performed pressure-dependent

polarization-resolved SHG measurements on a mechanically exfoliated 3R $MoS_2$ crystal as a control for a regular parallel-stacked TMD (Fig. R21). The measurements were conducted under the same DAC and optical conditions used for spiral $WS_2$, including hBN encapsulation, silicone-oil pressure medium, diamond anvils with 800 μm culets, and the same polarization-resolved SHG configuration.

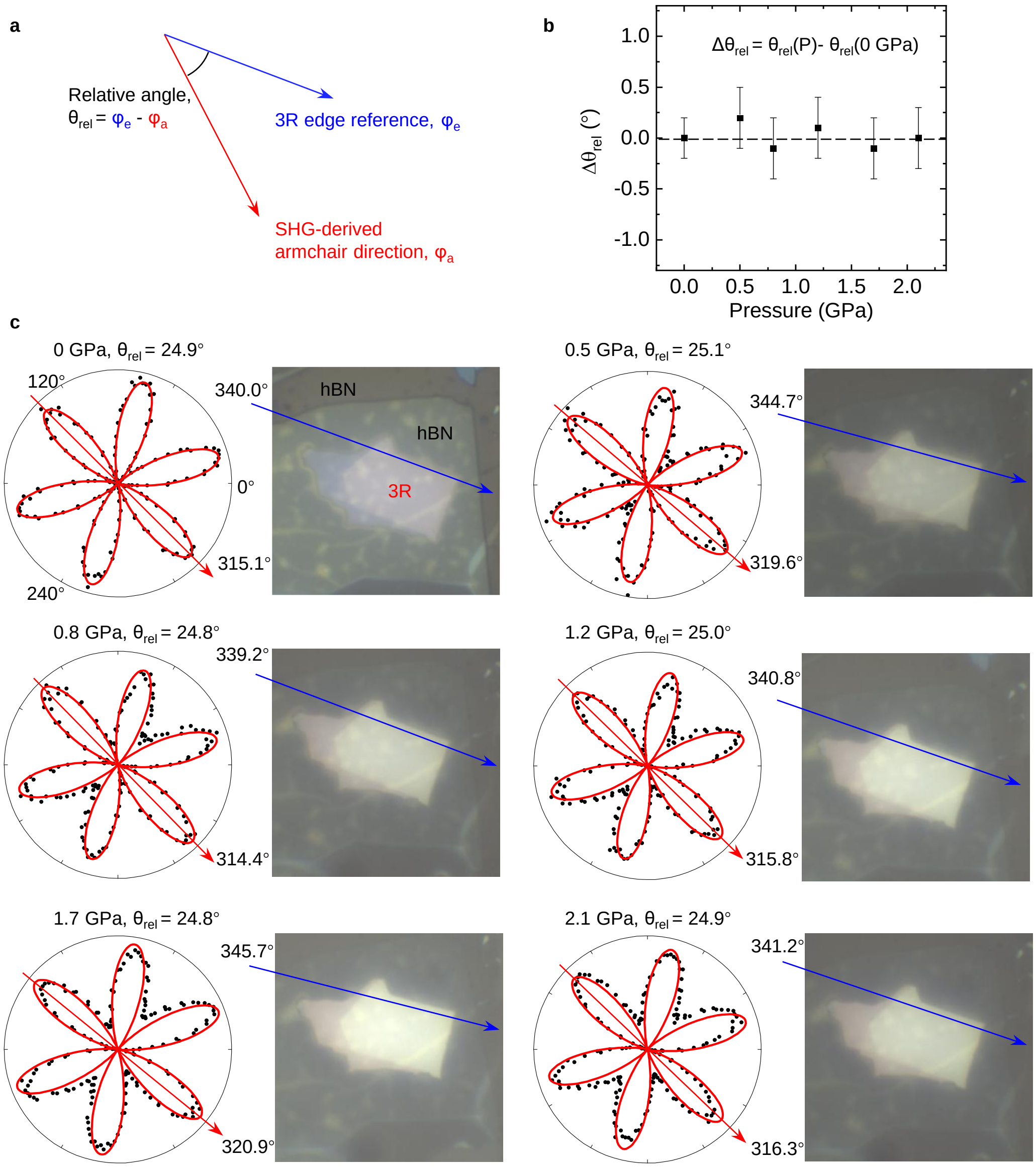


**Fig. R21 Pressure-dependent polarization-resolved SHG response of a regular 3R-stacked TMD reference. a**, Definition of the relative angle, $\theta_{rel} = \varphi_e - \varphi_a$, where $\varphi_e$ is the direction of a straight crystalline edge and $\varphi_a$ is the armchair direction extracted from the fitted SHG pattern. **b**, Pressure dependence of $\Delta\theta_{rel} = \theta_{rel}(P) - \theta_{rel}(0\ GPa)$ for mechanically exfoliated 3R $MoS_2$ measured under the same DAC and optical conditions as spiral $WS_2$. No systematic pressure-dependent rotation is observed within experimental uncertainty. **c**, Polarization-resolved SHG patterns and corresponding optical images of the 3R $MoS_2$ crystal measured at 0, 0.5, 0.8, 1.2, 1.7, and 2.1 GPa, respectively. Black symbols denote measured SHG intensities and red curves denote the corresponding fits. Blue arrows indicate the edge reference direction, whereas red

arrows indicate the SHG-derived armchair direction.

The 3R reference exhibits a well-defined sixfold SHG pattern throughout the pressure range from ambient pressure to 2.1 GPa (Fig. R21). To quantify any pressure-induced reorientation, we defined the relative angle as $\theta_{rel} = \varphi_e - \varphi_a$, where $\varphi_e$ is the direction of a straight crystalline edge and $\varphi_a$ is the armchair direction extracted from the fitted SHG pattern. We then evaluated $\Delta\theta_{rel} = \theta_{rel}(P) - \theta_{rel}(0\ \text{GPa})$. Within experimental uncertainty, $\Delta\theta_{rel}$ remains centred around zero over the entire pressure range. Thus, regular 3R stacking does not show a systematic pressure-dependent rotation of the SHG pattern relative to the crystallographic edge reference under the present experimental conditions.

This control has two implications. First, it confirms that the combined effects of compressed diamond, silicone oil, hBN encapsulation, and the polarization-resolved optical path do not produce an artificial rotation of the SHG pattern. Second, it shows that hydrostatic compression alone does not induce an observable reorientation in a regular 3R-stacked TMD. The pressure-dependent SHG rotation observed in spiral $WS_2$ is therefore attributed to its translation-disordered, metastable interlayer registry rather than to a generic response of parallel-stacked TMDs under pressure.

The above discussion has been incorporated into Supplementary Fig. 20.

1 Qiu, W., Zhang, B., Sun, Y., He, L. & Ni, Y. Atomic reconstruction enabled coupling between interlayer distance and twist in van der Waals bilayers. *Extreme Mechanics Letters* **69**, 102159, doi:https://doi.org/10.1016/j.eml.2024.102159 (2024).

2 Frenzel, T., Kadic, M. & Wegener, M. Three-dimensional mechanical metamaterials with a twist. *Science* **358**, 1072-1074, doi:doi:10.1126/science.aao4640 (2017).

3 Montazeri, A. & Mahnama, M. Twisting chiral mechanical metamaterials: A review. *Materials Today Communications* **44**, 112070, doi:https://doi.org/10.1016/j.mtcomm.2025.112070 (2025).

4 Fernandez-Corbaton, I. *et al.* New Twists of 3D Chiral Metamaterials. *Advanced Materials* **31**, 1807742, doi:https://doi.org/10.1002/adma.201807742 (2019).

5 Zhao, Y. *et al.* Supertwisted spirals of layered materials enabled by growth on non-Euclidean surfaces. *Science* **370**, 442-445, doi:doi:10.1126/science.abc4284 (2020).

6 Shearer, M. J. *et al.* Complex and Noncentrosymmetric Stacking of Layered Metal Dichalcogenide Materials Created by Screw Dislocations. *Journal of the American Chemical Society* **139**, 3496-3504, doi:10.1021/jacs.6b12559 (2017).

7 Zhao, Y. & Jin, S. Stacking and Twisting of Layered Materials Enabled by Screw Dislocations and Non-Euclidean Surfaces. *Accounts of Materials Research* **3**, 369-378, doi:10.1021/accountsmr.1c00245 (2022).

8 Shi, J. *et al.* 3R MoS2 with Broken Inversion Symmetry: A Promising Ultrathin Nonlinear Optical Device. *Advanced Materials* **29**, 1701486, doi:https://doi.org/10.1002/adma.201701486 (2017).

9 Ci, P. *et al.* Quantifying van der Waals Interactions in Layered Transition Metal Dichalcogenides from Pressure-Enhanced Valence Band Splitting. *Nano Letters* **17**, 4982-4988, doi:10.1021/acs.nanolett.7b02159 (2017).

10 Weston, A. *et al.* Atomic reconstruction in twisted bilayers of transition metal dichalcogenides. *Nature Nanotechnology* **15**, 592-597, doi:10.1038/s41565-020-0682-9 (2020).

11 Quan, J. *et al.* Phonon renormalization in reconstructed MoS2 moiré superlattices. *Nature Materials* **20**, 1100-1105, doi:10.1038/s41563-021-00960-1 (2021).

12 Woods, C. R. *et al.* Macroscopic self-reorientation of interacting two-dimensional crystals. *Nature Communications* **7**, 10800, doi:10.1038/ncomms10800 (2016).

13 Li, Y. *et al.* Probing Symmetry Properties of Few-Layer MoS2 and h-BN by Optical Second-Harmonic Generation. *Nano Letters* **13**, 3329-3333, doi:10.1021/nl401561r (2013).

14 Jung, C. *et al.* Highly Crystalline CVD-grown Multilayer MoSe2 Thin Film Transistor for Fast Photodetector. *Scientific Reports* **5**, 15313, doi:10.1038/srep15313 (2015).

15 Ly, T. H. *et al.* Vertically Conductive MoS2 Spiral Pyramid. *Advanced Materials* **28**, 7723-7728, doi:https://doi.org/10.1002/adma.201602328 (2016).

16 Yang, J., Lee, J.-U. & Cheong, H. Excitation energy dependence of Raman spectra of few-layer WS2. *FlatChem* **3**, 64-70, doi:https://doi.org/10.1016/j.flatc.2017.06.001 (2017).

17 Liang, L. *et al.* Low-Frequency Shear and Layer-Breathing Modes in Raman Scattering of Two-Dimensional Materials. *ACS Nano* **11**, 11777-11802, doi:10.1021/acsnano.7b06551 (2017).

18 Pizzi, G., Milana, S., Ferrari, A. C., Marzari, N. & Gibertini, M. Shear and Breathing Modes of Layered Materials. *ACS Nano* **15**, 12509-12534, doi:10.1021/acsnano.0c10672 (2021).

19 van Baren, J. *et al.* Stacking-dependent interlayer phonons in 3R and 2H MoS2. *2D Materials* **6**, 025022, doi:10.1088/2053-1583/ab0196 (2019).

20 Wu, J.-B. *et al.* Resonant Raman spectroscopy of twisted multilayer graphene. *Nature Communications* **5**, 5309, doi:10.1038/ncomms6309 (2014).

21 Du, G. *et al.* Interlayer engineering of lattice dynamics and elastic constants of 2D layered nanomaterials under pressure. *Nature Communications* **16**, 4901, doi:10.1038/s41467-025-60211-8 (2025).
22 Puretzky, A. A. *et al.* Twisted MoSe2 Bilayers with Variable Local Stacking and Interlayer Coupling Revealed by Low-Frequency Raman Spectroscopy. *ACS Nano* **10**, 2736-2744, doi:10.1021/acsnano.5b07807 (2016).
23 Zhao, Y. & Jin, S. Controllable Water Vapor Assisted Chemical Vapor Transport Synthesis of WS2–MoS2 Heterostructure. *ACS Materials Letters* **2**, 42-48, doi:10.1021/acsmaterialslett.9b00415 (2020).
24 Fan, X. *et al.* Broken Symmetry Induced Strong Nonlinear Optical Effects in Spiral WS2 Nanosheets. *ACS Nano* **11**, 4892-4898, doi:10.1021/acsnano.7b01457 (2017).
25 Fan, X. *et al.* Mechanism of Extreme Optical Nonlinearities in Spiral WS2 above the Bandgap. *Nano Letters* **20**, 2667-2673, doi:10.1021/acs.nanolett.0c00305 (2020).
26 Ci, P. *et al.* Breaking Rotational Symmetry in Supertwisted WS2 Spirals via Moiré Magnification of Intrinsic Heterostrain. *Nano Letters* **22**, 9027-9035, doi:10.1021/acs.nanolett.2c03347 (2022).
27 Ji, Z. *et al.* Opto-twistronic Hall effect in a three-dimensional spiral lattice. *Nature* **634**, 69-73, doi:10.1038/s41586-024-07949-1 (2024).
28 Wang, Q., Wang, X., Lou, Q., Jiang, Y. & Fan, X. Two-Dimensional Spiral: A Promising Moiré Superlattice. *Laser & Photonics Reviews* **19**, 2401368, doi:https://doi.org/10.1002/lpor.202401368 (2025).

**Point-by-point response to Reviewer #3's comments:**

**Q1:** *The supplementary data provided by the authors largely answered my question. However, I would also like to know the influence of diamond (DAC) on the polarization results during the measurement process regarding the SHG data.*

Response:

We thank the reviewer for this helpful comment. To evaluate whether the diamond anvils in the DAC influence the polarization-resolved SHG measurements, we performed additional diamond-background control measurements under the same DAC optical configuration.

During the previous response round, we measured hBN-encapsulated bulk 2H-stacked and 3R-stacked TMD samples in the DAC (Fig. R1a and b). To directly assess the diamond contribution, we further measured the SHG response from the diamond background at different pressures. The 0 GPa diamond-background data are plotted together with the 3R and bulk 2H data in Fig. R1b on the same intensity scale, showing that the diamond contribution is negligible compared with the 3R SHG response. The diamond signal also remains close to the noise level at 0.7, 1.6, and 2.4 GPa and shows no resolvable angular modulation (Fig. R1c).

Therefore, over the pressure range relevant to this work, the diamond anvils do not introduce a detectable SHG background or pressure-dependent polarization artifact in our measurements.

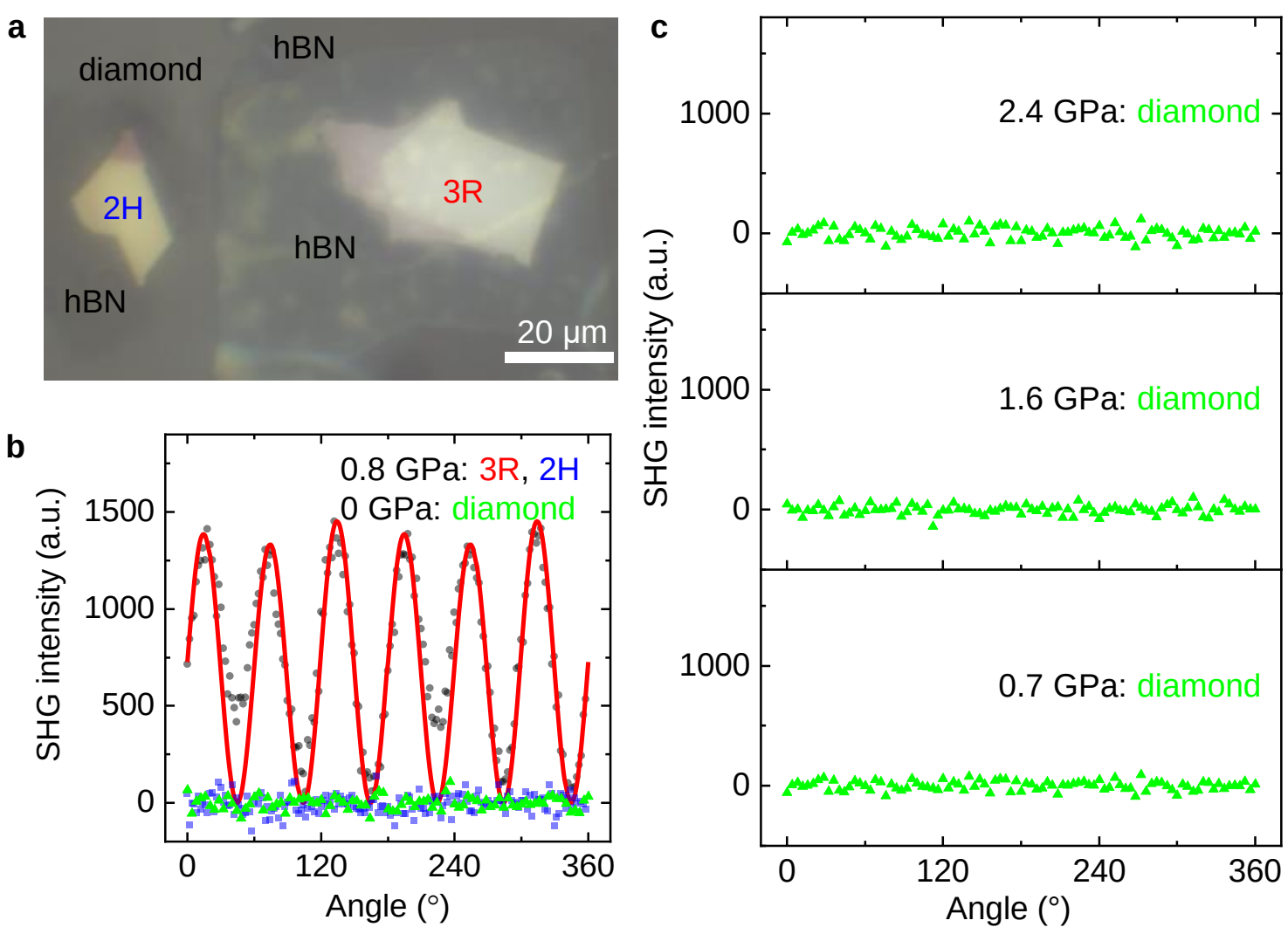


**Fig. R1 Control measurements of the diamond contribution to polarization-resolved SHG under DAC conditions. a**, Optical image of hBN-encapsulated bulk 2H-stacked and 3R-stacked TMD samples loaded in the DAC. Scale bar, 20 μm. **b**, Polarization-resolved SHG intensities measured from the 3R and bulk 2H regions at 0.8 GPa, together with a representative diamond-background signal measured at 0 GPa. **c**, Pressure-dependent diamond-background SHG signals measured at 0.7, 1.6, and 2.4 GPa using the same DAC optical configuration. The green diamond-background data in **b** and **c** were acquired from the same diamond-control loading batch, whereas the 3R and 2H data in **b** were acquired during the previous response round.
